# Biocatalytic Transamination of Aldolase‐Derived 3‐Hydroxy Ketones

**DOI:** 10.1002/adsc.202300201

**Published:** 2023-04-26

**Authors:** Mathias Pickl, Markus Ebner, Samantha Gittings, Pere Clapés, Wolfgang Kroutil

**Affiliations:** ^1^ Department of Chemical Biology Instituto de Química Avanzada de Cataluña (IQAC-CSIC) Jordi Girona 18-26 08034 Barcelona Spain; ^2^ Institute of Chemistry University of Graz Heinrichstrasse 28 8010 Graz Austria; ^3^ Prozomix Ltd. West End Industrial Estate Haltwhistle Northumberland NE49 9HA U.K

**Keywords:** Biocatalysis, Aldolase, Transaminase

## Abstract

Although optical pure amino alcohols are in high demand due to their widespread applicability, they still remain challenging to synthesize, since commonly elaborated protection strategies are required. Here, a multi‐enzymatic methodology is presented that circumvents this obstacle furnishing enantioenriched 1,3‐amino alcohols out of commodity chemicals. A Type I aldolase forged the carbon backbone with an enantioenriched aldol motif, which was subsequently subjected to enzymatic transamination. A panel of 194 TAs was tested on diverse nine aldol products prepared through different nucleophiles and electrophiles. Due to the availability of (*R*)‐ and (*S*)‐selective TAs, both diastereomers of the 1,3‐amino alcohol motif were accessible. A two‐step process enabled the synthesis of the desired amino alcohols with up to three chiral centers with *de* up to >97 in the final products.

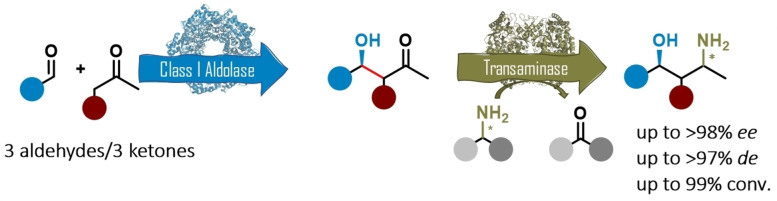

## Introduction

The combination of multiple stereoselective biocatalysts in a reaction sequence enables the production of complex organic molecules with multiple chiral centers out of simple achiral commodity chemicals.[^1^, ^2^, ^3^, ^4^, ^5^, ^6^, ^7^] In many synthetic routes involving biocatalysis, standard chemical methods are responsible for building up the carbon framework while the use of enzymes is limited to a functional group interconversion.[^4^, ^5^, ^6^, ^7^, ^8^] This strategy can be expanded when an aldolase is used for the C−C bond formation, providing a carbonyl group which enables a multitude of follow‐up chemistry performed by redox enzymes.[^9^, ^10^] Lyases or transferases suitable for C−C bond formation are fairly diverse, among them thiamine‐dependent carboligases,^[11]^ PLP‐dependent aldolases,^[12]^ metal‐dependent carboligases^[13]^ and Type I and Type II aldolases in which the nucleophile component is activated via Schiff base intermediate that evolves to an enamine, or employing a transition metal as a Lewis‐acid cofactor to give the enolate intermediate, respectively.^[14]^ A prominent example of a potent carboligase is the Type I aldolase d‐fructose‐6‐phosphate aldolase from *E. coli* (*Ec*FSA), which in its supposedly natural reaction cleaves reversibly in a retro‐aldol fashion phosphate sugars into dihydroxyketone and d‐glyceraldehyde‐3‐phosphate.^[14]^ Substrate probing and protein engineering enabled a large scope for both, phosphorylated and unphosphorylated electrophiles and nucleophiles.^[15]^ Hence, the wild‐type *Ec*FSA and variants thereof are perfectly suitable in multistep routes towards the synthesis of functionalized carbon backbones. The resulting 3‐hydroxycarbonyl compounds are ideal intermediates to access chiral 1,3‐amino alcohols, a motif found widespread among bioactive molecules.^[16]^ Among others, IREDs[^17^, ^18^], amine dehydrogenases,^[19]^ and transaminases (TAs) are suited for such a transformation.[^20^, ^21^, ^22^] A plethora of synthetic routes were developed to access chiral 1,3‐amino alcohols fueled by the high utility of these structural motives present in many compounds of pharmaceutical and biological interest.[^23^, ^24^, ^25^, ^26^, ^27^] Besides purely synthetic one's such as the reduction of asymmetric 3‐hydroxy‐imines or 3‐amino‐ketones,[^28^, ^29^, ^30^] but also e. g. hydroamination of allylic alcohols turned out to be successful.[^31^, ^32^] Biocatalytic counterparts rely either on a chemically synthesized diketone starting material,^[33]^ do not give a 1,3‐substitution pattern,^[34]^ or a transketolases was used to build the carbon framework which requires hydroxypyruvate, an expensive reagent only available in situ through an additional multistep cascade or hydroxyamino acids were obtained.^[33,35–41^

**Scheme 1:**
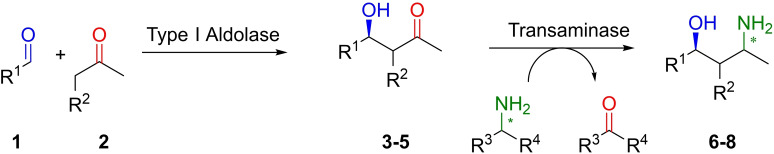
Biocatalytic aldol‐amination sequence to access 1,3‐amino alcohols out of achiral starting material mediated by a Type I aldolase and a transaminase.

In this contribution we established a biocatalytic synthetic route towards chiral 1,3‐amino alcohols, starting from achiral molecules (Scheme 1). The approach is demonstrated on the synthesis of eleven amino alcohols and allows accessing two diastereomers based on the TA stereoselectivity.

## Results and Discussion

**Scheme 2:**
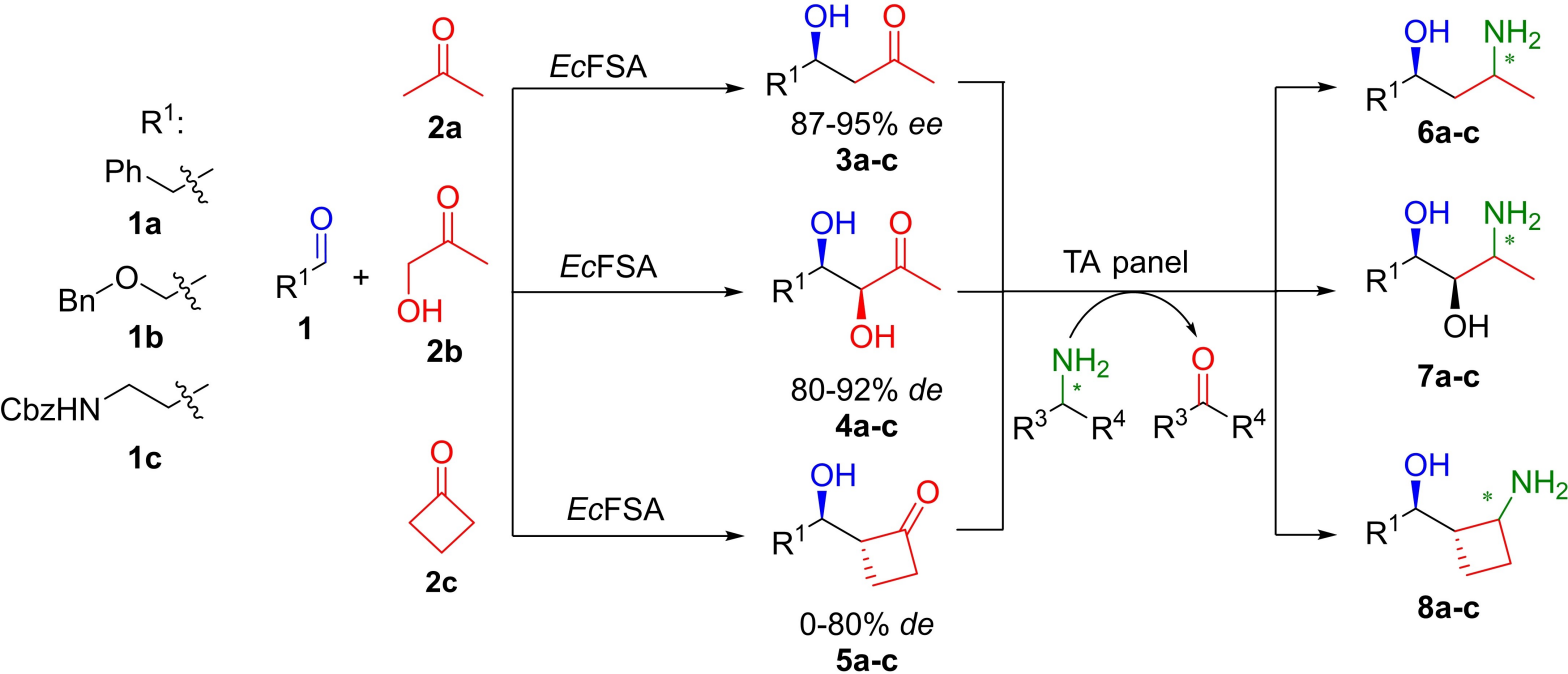
Investigated aldol product scope (3–5) furnished by wild type *Ec*FSA or *Ec*FSA D6 N variant catalysis (see experimental section) and potential 1,3‐amino alcohol (6–8) products after the enzymatic transamination reaction; ee and de determined on chiral stationary phase HPLC or NMR, respectively.

The aldol intermediates **3**–**5** were biocatalytically synthesized according to literature described protocols (Scheme 2).[^15^, ^42^, ^43^] Three aldehydes **1 a**–**c** were combined with three donors **2 a**–**c** to demonstrate the flexibility in nucleophile and electrophile scope of *Ec*FSA. Aliphatic aldehydes were omitted, however, would also be in scope for *Ec*FSA variants.^[44]^ Depending on the nucleophile, either wild type *Ec*FSA or *Ec*FSA D6 N variant^[15]^ was employed. The isolated yields were between 15 and 70% depending on the applied nucleophile. To ensure that no racemization takes place, *ee* was monitored over time (See SI, Figure S1), but the *ee* remained constant. The provided nine aldol products were then subjected to a TA panel to find a suitable candidate to furnish the desired 1,3‐amino alcohols.

A commercial library containing 192 TAs provided by Prozomix Ltd. as lyophilized cell free extracts (CFEs) was initially screened with the aldol products (*R*)‐**3 a**, (*S*)‐**3 b** and (*R*)‐**3 c** (25 mM). The reactions were stopped after a defined time (24 h) using d/l‐alanine as amino donor in excess (15 eq, 375 mM) to drive the equilibrium towards product formation, a strategy which was successfully applied in a previous study.^[37]^ The screening, monitored by HPLC‐UV, identified 12 TAs, which were then included in a minimal panel (See SI, Table S1).

**Scheme 3:**
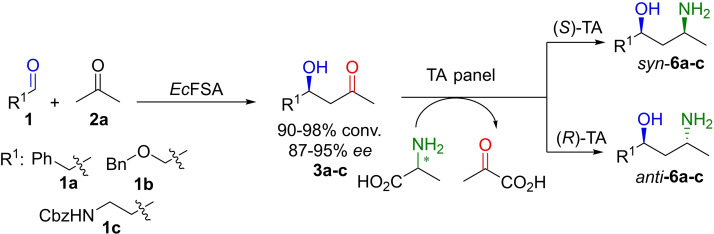
Transamination of **3 a**–**c** with (*R*)‐ or (*S*)‐ selective TAs with the formation of the possible diastereomers according to the enantiopreference of the TAs.

Based on the retention time/elution order of the transamination product **6 b** on GC‐MS (Supplementary Figure S3) and on previously described stereopreference of the active enzymes we assumed that the TAs from the kit showing activity on the aldol panel were exclusively (*S*)‐selective for the newly created center in the diastereomers.^[37]^ This was also confirmed by NMR after product isolation (vide infra).^[37]^ Thus, the *syn*‐configured 1,3‐amino alcohol **6 b** was found (Scheme 3). The TAs from *Aspergillus terreus* (*At*TA) and from *Arthrobacter sp*. (*Arthr*TA) were suspected to provide the stereocomplementary (*R*)‐amine.[^45^, ^46^] Indeed, due to the (*R*)‐configuration of the hydroxy group in the aldol motif of **3 a** and **3 c**, the *anti*‐product was found after transamination possessing an (*R*,*R*)‐configuration. In case of aldol intermediate **3 b**, the switch of the CIP priority led to the (*S*)‐configuration of the hydroxy group^[42]^ giving the (*R,S*)‐1,3‐amino alcohol (Scheme 3).

Encouraged by these results, a panel of six additional aldol products **4 a**–**c** and **5 a**–**c** being sterically more demanding was synthesized. The previously described panel of TAs derived from the initial screening was expanded by *At*TA and *Arthr*TA. The aldol products **4** and **5** were subsequently subjected to this TA minimal panel. The dihydroxyketone substrates **4** were overall better accepted than the substrates **5** containing the bulkier cyclobutanone moiety (Table 1).

**Table 1:** Heatmap of endpoint conversion for transamination of aldol panel 3–5 with (R)‐selective in‐house TAs and previously identified minimal panel of the commercial TA library. Amine product: light green: conv. <20%, green: conv. 20–60% dark green: conv. >60%. Conditions: lyophilized whole cells in case of *Arthr*TA or *At*TA (4 mg) or CFE (5 mg), 3–5 (25 mM), d‐ or l‐alanine (375 mM), PLP (1 mM), Kp_i_ buffer (50 mM, pH 7), total volume 200 μL, rt, horizontal shaking, 24 h.

	Amino donor	**3 a**	**3 b**	**3 c**	**4 a**	**4 b**	**4 c**	**5 a**	**5 b**	**5 c**
ArthrTA	d‐alanine									
AtTA	d ‐alanine									
TA26	l‐alanine	42,739								
TA31	l‐alanine	39,633								
TA33	l‐alanine	5,033								
TA81	l‐alanine	41,361								
TA93	l‐alanine	49,994								
TA166	l‐alanine	38,621								
TA169	l‐alanine	37,283								
TA187	l‐alanine	4,061								
TA188	l‐alanine	15,66								
TA189	l‐alanine	65,981								
TA191	l‐alanine	53,75								
TA192	l‐alanine	18,849								

Thereby the *At*TA appeared to be slightly more active than *Arthr*TA on the selected aldol products. Nevertheless, the minimal enzyme panel from the commercial kit was of interest. An enzyme which displayed activity for **3** and **4** was TA166. Particularly, TA192 was found to be very active towards dihydroxy ketone **4**, and also for **3 b** and **3 c** bearing BnO‐ and CbzNH‐ moieties on the main chain, respectively. Furthermore, TA31, and TA169 were among the most active candidates (Table 1).

**Figure 1:**
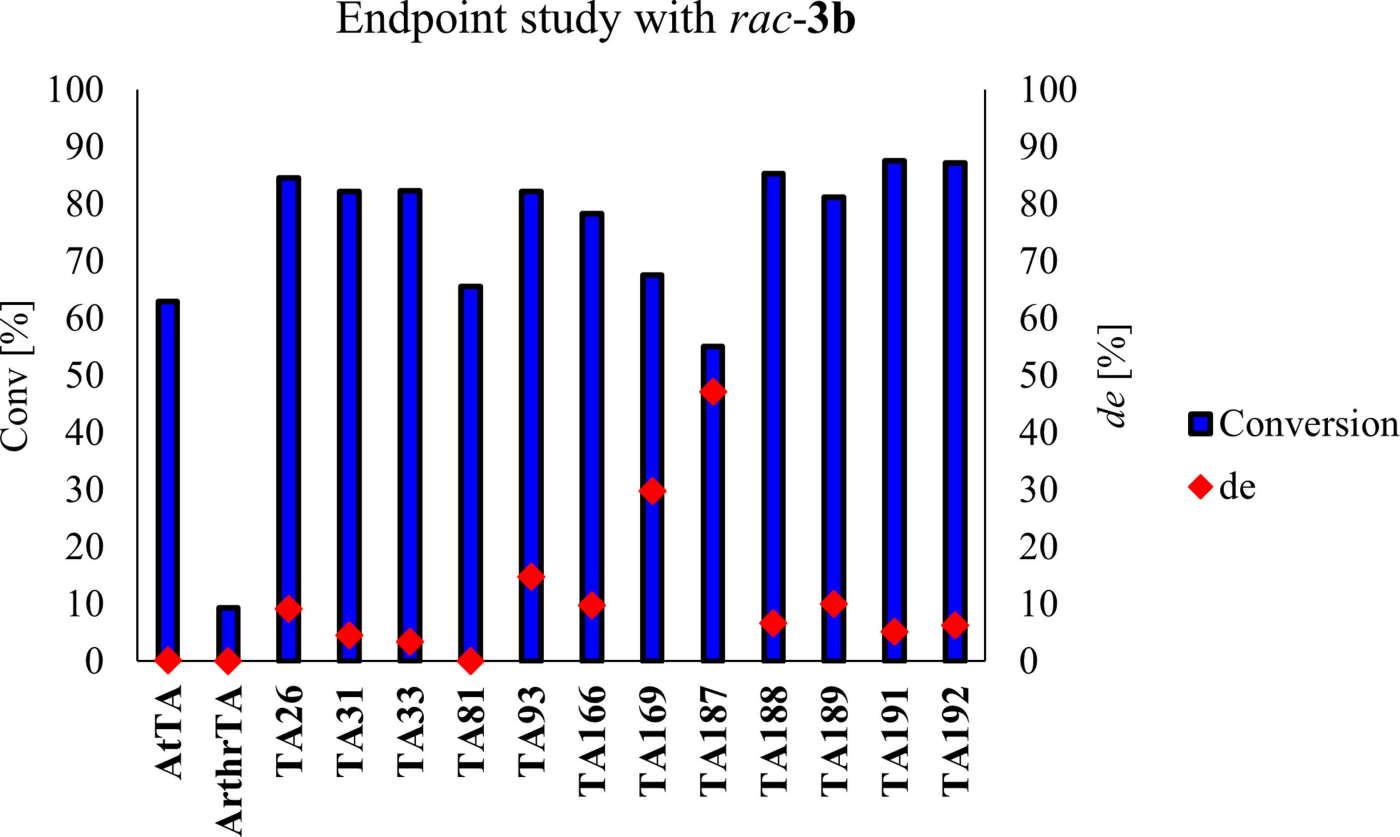
Diastereomeric excess at specific conversions of rac‐3b with minimal panel of TAs. Conditions: lyophilized whole cells in case of *At*TA and *Arthr*TA (4 mg) or CFE for TA‐Prozomix (5 mg), rac‐3b (25 mM), d‐ or l‐alanine (375 mM), PLP (1 mM), Kp_i_ buffer (50 mM, pH 7), total volume 200 μL, rt, horizontal shaking, 24 h.

Concerning the enantiopreference, most of the TAs revealed only minimal discrimination for one of the two enantiomers of **3 b** (*rac*‐**3 b** was prepared by chemical methodology, see SI) (Figure 1). Only TA169 with a *de* of 30% and TA187 with a *de* of 47% showed a certain enantioselectivity.

**Figure 2:**
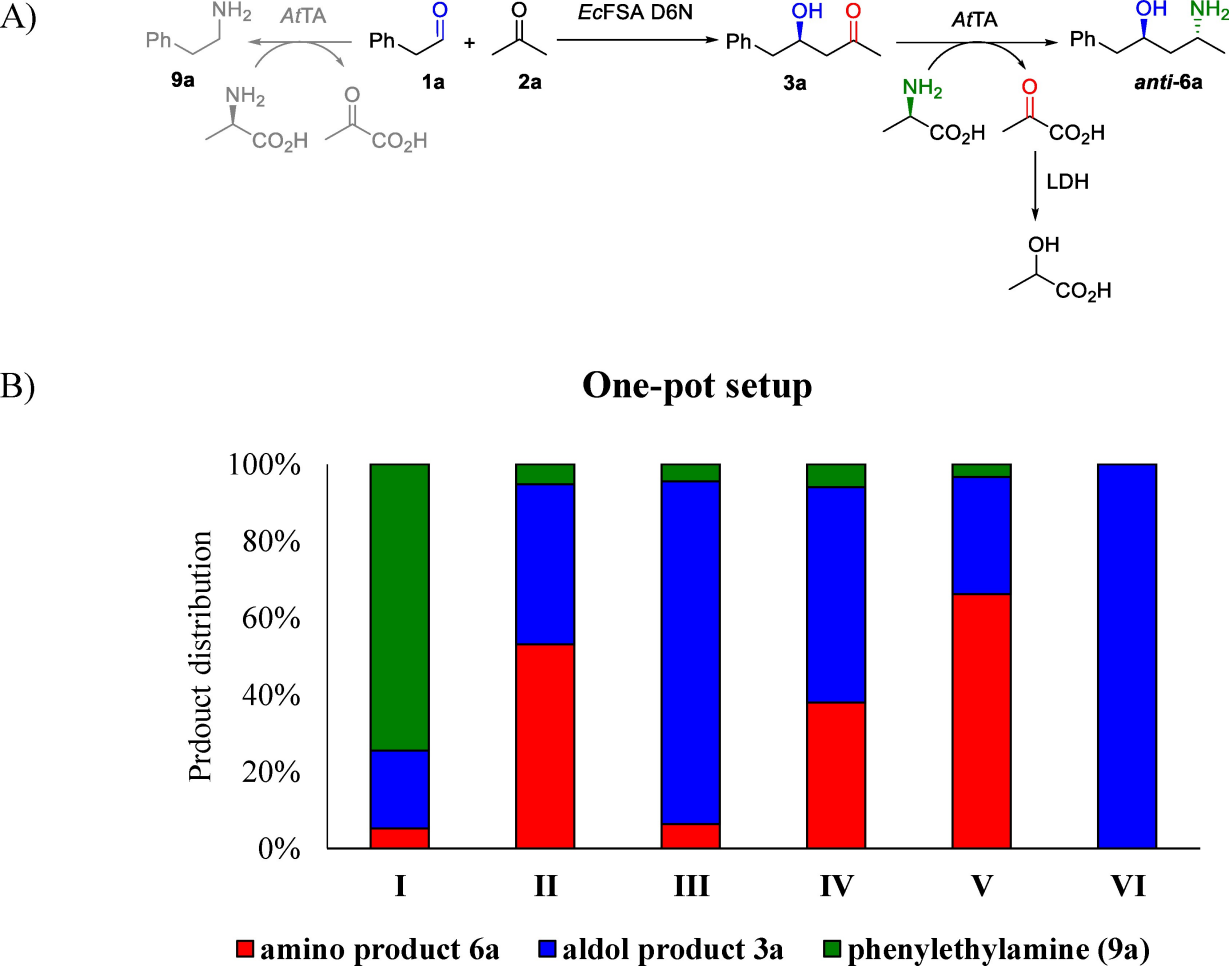
Aldol addition of acetone (2a) to phenylacetaldehyde (1a) catalyzed by *Ec*FSA D6 N and subsequent transamination reaction catalyzed by *At*TA in the presence of d‐Ala. A) One pot reaction of aldol addition and subsequent transamination of 1a and 2a with *At*TA mediated side reaction to 9a. B) Product distribution of the aldol reaction and the subsequent transamination in a one‐pot setup with following conditions: (I) One‐pot concurrent cascade; (II) One‐pot, two‐step cascade, *Ec*FSA removed via heat‐shock after completion of aldol addition and then *At*TA added; (III) One‐pot, two‐step cascade, 15 eq d‐alanine instead of pyruvate removal with LDH and no *Ec*FSA elimination (IV) One‐pot, two‐step cascade in the presence of *Ec*FSA; (V) One‐pot, two‐step cascade, *Ec*FSA removed by heat‐shock and acetone purged by airflow; (VI) no *At*TA added; i. e. control experiment. Reaction conditions: Triethanolamine (TEoA) buffer (50 mM, pH 8), horizontal shaking, 25 °C, 80 mM substrate (initial, 50 mM after *At*TA addition), lactate dehydrogenase (LDH) to eliminate the pyruvate formed after the transamination reaction.

The potential combination of enzymes in a one‐pot fashion in a cascade set‐up is a key benefit of biocatalysis towards conventional chemistry.^[1]^ Hence, to perform the reaction one‐pot with the in‐situ transamination of the aldol product is indeed very attractive. After testing *At*TA at the adapted conditions suitable for the cascade (i. e., change of buffer, slightly more basic pH, results not shown), a screening was performed to investigate the compatibility of the two reaction steps in one‐pot (Figure 2) with substrate **1 a** and **2 a** as example.

A one‐pot procedure performing both reaction steps simultaneously provided mainly 2‐phenylethylamine (**9 a)** indicating that *At*TA prefers **1 a** over aldol product **3 a** as substrate (Figure 2B, experiment **I**). In the two‐step procedure, performing the two steps in a subsequent fashion, the aldol product was the main product **(**Figure 2B, experiments **III** and **IV**). In selected cases, experiment **II** and **V**, the aldolase was precipitated by heat‐shock and then removed via centrifugation which improved the formation of **6 a**. The highest product formation (66%) was achieved with additional purging of the acetone via airstream **(**Figure 2B, experiment **V**). Overall, employing LDH for pyruvate removal led to better results than using excess of alanine to shift the equilibrium towards amine formation (6%) (Figure 2B, **III**).

**Scheme 4:**
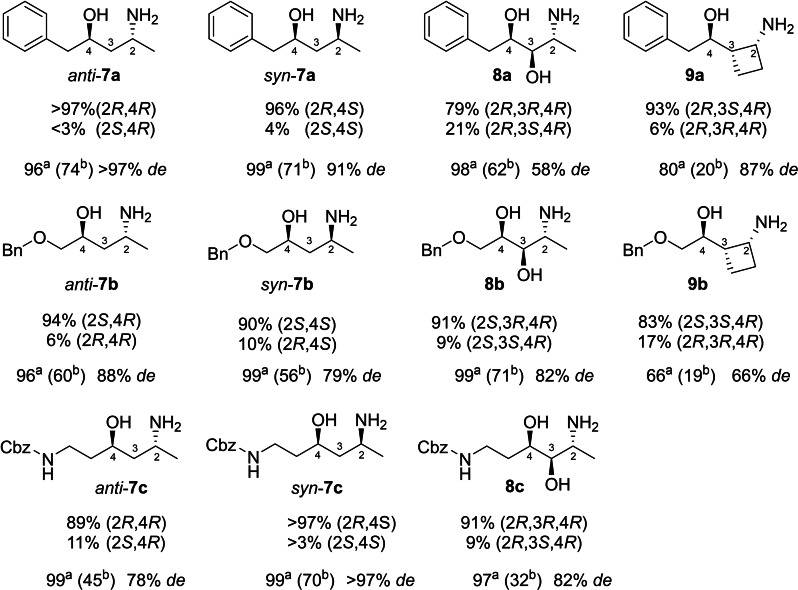
1,3‐Amino alcohols synthesized at laboratory scale by the two‐step procedure with co‐substrate removal/recycling. ^a^Conversion determined by HPLC‐UV. ^b^Isolated yield. de of the isolated products were determined by NMR.

To characterize the desired 1,3‐amino alcohols **6**–**8**, the transamination was performed at preparative scale (Scheme 4). To obtain the anti‐diastereomer *At*TA was employed, for the syn‐diastereomer TA166. TA166 was found in a genomic library and a BLAST search identified that it is closely related to TA from *Burkholderia pyrrocinia*. The equilibrium of the reactions was shifted to the side of the product either by recycling/removal of pyruvate using an alanine dehydrogenase (AlaDH) or with LDH.^[47]^ The obtained conversions reached almost completion for products **6 a**–**c** (96–99% conv.) with moderate to good isolated yields. The *de* found for products **7 a**–**c** which were isolated with moderate yields (32–71%). In case of products **8 a**–**b**, the conversion showed a significant drop to 66–80%, with meager isolated yields (19–20%). It is assumed that this is due to a reduced efficiency during the work‐up operations due to low extraction efficiency. The increased *de* for **8 a**–**b** confirms that the TA can discriminate between the two diastereomers of the aldol product. The diastereoselectivity of the products were determined via NMR and for product *anti*‐**7 a** and *syn*‐**7 c** perfect *de* was found. Indicating, that the TA again provides steric discrimination.

## Conclusions

In summary, a bienzymatic route is described in which enantioenriched 1,3‐amino alcohols were produced starting from achiral compounds. The key aldol addition exploited the expansion of the nucleophilic scope of wild type *Ec*FSA and variants, while the transamination of the resulted ketone group mediated by stereocomplementary TAs furnished both diastereomers of the amine. The initial screening of a TA panel revealed that only a rather small subset of enzymes was capable to aminate the aldol products. A key finding was that the two transformations can take place in the same vessel, however only sequentially. The selection of the TA for the transamination turns out to be crucial for the overall de of the final product as in some cases a preference towards one of the enantiomers of the aldol products was observed. The results successfully demonstrated that the designed enzymatic route could serve as a tool to access demanded chiral 1,3‐amino alcohols directly from racemic starting material. This protocol offers a benign alternative to established methods, providing both diastereomers in good yields.

## Experimental Section

### General

All chemicals were ordered from Sigma Aldrich (Steinheim, Germany) and all solvents from Acros (Geel, Belgium). Prozomix (Haltwhistle, UK) provided the commercial TA kit. GDH was provided from DSM (7 U/mg), FDH from evocatal (Evo 1.1.230, 1.4 U/mg). TLC analysis was performed on pre‐coated silica gel 60F_254_ and compounds were visualized by UV, ninhydrin, ceric ammonium molybdate or anisaldehyde staining. Flash column chromatography was carried out using Merck silica gel 60 (particle size 0.040 –0.063 mm). Optical rotation was measured at 20 °C on an Anton Paar MCP 5100 Polarimeter against the sodium D‐line. All NMR spectra were recorded using a Bruker NMR at 300 or 500 (1H) and 75 (13 C) MHz in CDCl_3_ or d_4_‐MeOH using the residual solvent peak as reference. Chemical shifts are reported in ppm and coupling constants (*J*) are given in Hz. High resolution MS measurements (HR‐MS) were carried out on an Agilent 6230 TOF LC/MS system in ESI^+^ mode. Purified recombinant l‐alanine dehydrogenase was prepared as described previously (11 U/mg).^[47]^

### Analytical methods

*HPLC reaction monitoring*. Measurements were carried out with the Agilent 1260 infinity System with a C18 (2) 100 Å Luna Phenomenex column (250 mm×4.6 mm×5 μm) and a DAD (G4212B) for the UV detection at 210 or 254 nm. The flow rate was 1 mL/min at 30 °C for 30 min with a gradient of the solvent B of 10%–100%, solvent system: 0.1%% (v/v) TFA in H_2_O and solvent B: 0.1% (v/v) TFA in CH_3_CN/H_2_O 4:1. The amount of product and substrates was quantified from the peak areas using an external standard methodology and calibration curves.

*Chiral HPLC measurements*. Enantiomeric excesses (*ee*) were determined using HPLC analysis on chiral stationary phase (CHIRALPAK® IC 4.6×250 mm column, 5 μm, flow rate 1.0 mL min^−1^ at 20 °C and UV detection (210 nm), isocratic elution hexane/*i*PrOH 75:25.

*GC‐MS derivatization and reaction monitoring*. All GC‐MS measurements were carried out with an Agilent 1909 1S‐433:30 GC system, equipped with a HP‐5MS, 5% phenyl methyl silox column (30 m×0.25 mm×0.25 μm) and an Agilent 5975c mass detector (electron impact: 70 eV). The carrier gas was helium at a flow rate of 0.7 mL/min and the temperature program was 100 °C–300 °C with a gradient of 10 °C/min.

*GC‐MS sample derivatization*. 150 μL of the reaction mixture was quenched by 15 μL 10 M NaOH. The product was extracted with 150 μL ethyl acetate, the organic phase dried Na_2_SO_4_ and *N*‐methyl‐bis(trifluoroacetamide) (MBTFA) (45 μL) was added. The reaction was carried out at 60 °C for 45 min with horizontal shaking.

### Enzyme Production and Activity Tests

*Production of FSA variants*. The expression and purification of FSA variants was performed as described before with small adoptions.^[48]^

A freshly transformed single colony of *E. coli* M‐15 [pREP‐4] (QIAGEN) was used to inoculate 10 mL LB/Kan/Amp for an overnight culture (ONC) and shaken overnight at 37 °C and 120 rpm in an orbital shaker. The 1 L main culture (LB/Amp/Kan) was inoculated with 10 mL ONC and cultivated (37 °C, 150 rpm) to an OD_600_ of 0.7. The expression was induced by IPTG (1 mM) and the culture was shaken at 30 °C and 150 rpm for 18 h. The cells were harvested by centrifugation (20 min, 12000×g, 4 °C) and the pellet resuspended in Gly‐Gly buffer (35 mL, 100 mM, pH 8.5, 1 mM DTT). The cells were lysed using the Branson Digital Sonifier SFX 250 (30% amplitude, 30 s pulse, 30 s pause, 7.5 min) and centrifuged to clear the lysate (45 min, 20000×g, 4 °C). The clear supernatant was treated by heat shock (70 °C, 30 min), then centrifuged again (45 min, 20000×g, 4 °C) The supernatant was dialyzed against GlyGly buffer (5 mm, pH 8.5) that contained DTT (0.1 mm) and finally lyophilized. ∼600 mg of the purified variants was obtained with 0.6 μg protein per μg lyophilized powder.

*Production of TAs whole cells. At*TA and *Arthr*TA were prepared as described previously.^[47]^

*Determination of activity of TAs from the Prozomix kit*. The activity of TA from Prozomix was determined in duplicates. 25 mg of the TA, as cell free extract preparations, was rehydrated in potassium phosphate buffer (50 mM, pH 7), PLP (1 mM), NAD^+^ (1 mM). Then alanine (125 μL, 125 mM, 5 eq.), glucose (150 μL, 150 mM), LDH (90 U) and glucose dehydrogenase (30 U) were added.

Substrate *rac*‐**3 b** (25 mM) was added, mixed, and put into the thermoshaker (25 °C, horizontal shaking). After 5, 10, 20, 40 and 60 minutes an aliquot of 150 μL of the reaction mixture was withdrawn and immediately quenched with 15 μL 10 M NaOH and extracted with EtOAc (150 μL). The sample was then further derivatized for a GC‐MS measurement.

*Determination of activity of TAs as cell free extracts (CFE) preparations*. The activity of the (*R*)‐selective TA was determined in the same way as the (*S*)‐selective TA from Prozomix with the difference that 20 mg of the TA was used. Additionally, the enzyme was rehydrated by shaking for 30 min and 120 rpm at 30 °C in an orbital shaker.

*Endpoint screening of TA screening with AlaDH recycling system*. The procedure was adapted from Mutti et al.^[47]^

Lyophilized cells of *E. coli* BL21(DE3) containing overexpressed TA (4 mg) were rehydrated in an microcentrifuge tube (1.5 mL) in phosphate buffer (100 μL, 50 mM, pH 7, 1 mM PLP, 1 mM NAD^+^) for 30 min at 30 °C and 120 rpm on an orbital shaker. Alternatively, lyophilized cell‐free extract of TA‐Prozomix (5 mg) were added to phosphate buffer 500 μL 50 mM, pH 7, 1 mM PLP, 1 mM NAD^+^), then ammonium formate (30 μL, 150 mM), d‐alanine (125 mM, 25 μL), L‐alanine dehydrogenase (2.4 U), formate dehydrogenase (2.0 U) and ketone 1 (25 mM) were added and the volume was adjusted to 1 mL with water. The mixture was shaken at 25 °C and 1000 rpm. The reaction was stopped after 24 h and analyzed via HPLC or GC‐MS after derivatization.

*Endpoint screening of TA with LDH pyruvate removal system*. The procedure was adapted from Mutti et al.^[47]^

Lyophilized cells of *E. coli* BL21(DE3) containing overexpressed TA (20 mg, 120 mU) were rehydrated in an microcentrifuge tube (1.5 mL) in phosphate buffer (500 μL, 50 mM, pH 7, 1 mM PLP, 1 mM NAD^+^) for 30 min at 30 °C and 120 rpm on an orbital shaker. Alternatively, TA‐Prozomix CFE (25 mg, 160 mU) were added to phosphate buffer 500 μL 50 mM, pH 7, 1 mM PLP, 1 mM NAD^+^), then D/L‐alanine (125 mM, 125 μL), d‐glucose (150 mM, 150 μL), GDH (43 μL, 30 U) LDH (90 μL, 90 U) and ketone **1** (25 mM) were added. The mixture was shaken at 25 °C and 1000 rpm. The reaction was quenched after 24 h by the addition of aqueous 1 N NaOH (200 μL) and the reaction mixture was extracted with ethyl acetate (2×500 μL). The combined organic phases were dried (Na_2_SO_4_) and the conversion was measured by GC‐MS after derivatization.

*Synthesis of Aldol products with nucleophile* **2 a**. The procedure was adapted from Roldán et al.^[42]^

The reaction was conducted in a 50 mL screw capped conical‐bottom polypropylene tubes. To an FSA D6 N solution (60 mg, 2.7 kU) in plain water (16 mL), Triethanolamine (TEoA) buffer pH 8 (1 mL) was added. To this solution, aldehyde **1** (1.6 mmol, 80 mM final concentration) dissolved in **2 a** (3 mL, 15% v/v) was added. Total reaction volume was 20 mL. Reaction mixtures were shaken (700 rpm) at 25 °C until completion was detected on TLC. After that, the reaction was stopped with MeOH (30 mL) to precipitate the enzyme. Then, the mixture was filtered through Celite^®^, the MeOH was evaporated, and the aqueous residue was lyophilized, purified, and characterized. Purification was conducted by silica gel column chromatography and eluted with a step gradient of hexane:EtOAc.

Alternatively, after MeOH was evaporated, the aqueous phase was saturated with NaCl. The mixture was then extracted with dichloromethane (3x50 mL). The organic phase was dried over anhydrous Na_2_SO_4_ and the solvent was evaporated. The product was purified by silica gel column chromatography and eluted with a step gradient of hexane:EtOAc.

*Synthesis of Aldol products with nucleophile* **2 b**. The procedure was adapted from Garrabou et al.^[49]^

The reaction was conducted in a 50 mL screw capped conical‐bottom polypropylene tubes. To an FSA wild type solution (60 mg, 2.7 kU) in plain water (19.3 mL), TEoA buffer pH 8 (1 mL) was added. The aldehyde **1** (1 mmol, 50 mM final concentration) was dissolved in DMF (0.4 mL, 2% v/v) and **2 b** (1 mmol, 50 mM final concentration) was then added. Reaction mixtures were shaken (700 rpm) at 25 °C. Reaction mixtures were shaken (700 rpm) at 25 °C until completion was detected on TLC. After that, the reaction was stopped with MeOH (30 mL) to precipitate the enzyme. Then, the mixture was filtered through Celite, the MeOH was evaporated, and the aqueous residue was lyophilized, purified, and characterized. The product was purified by silica gel column chromatography and eluted with a step gradient of hexane:EtOAc.

Alternatively, after MeOH was evaporated, the aqueous phase was saturated with NaCl and brine (20 mL) were added. The mixture was then extracted with dichloromethane (3x50 mL). The organic phase was dried over anhydrous Na_2_SO_4_ and the solvent was evaporated. The product was purified by silica gel column chromatography and eluted with a step gradient of hexane:EtOAc.

*Synthesis of Aldol products with nucleophile **2 c***.

The reaction was conducted in a 50 mL screw capped conical‐bottom polypropylene tubes. To an FSA D6 N solution (60 mg, 2.7 kU) in plain water (18 mL), TEoA buffer pH 8 (1 mL) was added. To this solution, aldehyde **1** (1.6 mmol, 80 mM final concentration) dissolved in **2 c** (1 mL, 5% v/v) was added. Total reaction volume was 20 mL. Reaction mixtures were shaken (700 rpm) at 25 °C until completion was detected on TLC after 96 h. After that, the reaction was stopped with MeOH (30 mL) to precipitate the enzyme. Then, the mixture was filtered through Celite, the MeOH was evaporated, and the aqueous residue was lyophilized, purified, and characterized. The product was purified by silica gel column chromatography and eluted with a step gradient of hexane:EtOAc.

Alternatively, after MeOH was evaporated, the aqueous phase was saturated with brine. The mixture was then extracted with dichloromethane (3x50 mL). The organic phase was dried over anhydrous Na_2_SO_4_ and the solvent was evaporated. The product was purified by silica gel column chromatography and eluted with a step gradient of hexane:EtOAc.

*Representative preparative transformation of anti‐**6** and **7**–**8** employing AtTA*. The procedure was adapted from Mutti et al.^[5]^

Lyophilized cells of *E. coli* BL21(DE3) containing overexpressed *At*TA (400 mg, 2.4 U) were rehydrated in a 50 mL screw capped conical‐bottom polypropylene tubes in phosphate buffer (50 mM, pH 7, 1 mM PLP, 1 mM NAD^+^) for 30 min at 30 °C and 120 rpm on an orbital shaker. Then, d‐alanine (125 mM), ammonium formate (150 mM), AlaDH (110 U), FDH (220 U) and ketone **3**–**5** (0.5 mmol, 25 mM) dissolved in DMSO or MTBE (5% v/v) were added. The mixture was shaken at 25 °C and 700 rpm for 24 h. After completion, the reaction mixture was saturated with NaCl, and the pH was adjusted to 3.7. Then, EtOAc (20 mL) and 2 spoons of celite was added the mixture was shaken for 15 min at 700 rpm. After a filtration through a water wetted pad of celite the phases were separated, and the aqueous phase was adjusted to pH 12. The product was extracted with EtOAc (5×20 mL). The combined organic phases were dried over Na_2_SO_4_ and the solvent evaporated until dryness.

*Representative preparative transformation of syn‐**6** employing TA166*. The procedure was adapted from Mutti et al.^[5]^

Lyophilized CFE of TA166 (250 mg, 1.2 U) were added to phosphate buffer (50 mM, pH 7, 1 mM PLP, 1 mM NAD^+^). Then, l‐alanine (125 mM), d‐glucose (150 mM), LDH (900 U) GDH (300 U) and ketone **3** (0.5 mmol, 25 mM) dissolved in DMSO or MTBE (5% v/v) were added for 24 h. The mixture was shaken at 25 °C and 700 rpm. After completion, the reaction mixture was saturated with NaCl, and the pH was adjusted to 3.7. Then, EtOAc (20 mL) and 2 spoons of celite was added the mixture was shaken for 15 min at 700 rpm. After a filtration through a water wetted pad of celite the phases were separated, and the aqueous phase was adjusted to pH 12. The product was extracted with EtOAc (5×20 mL). The combined organic phases were dried over Na_2_SO_4_ and the solvent evaporated.

**(*R*)‐4‐Hydroxy‐5‐phenylpentan‐2‐one (3 a)** 137 mg isolated yield (48%), 95% *ee*, colorless oil. **Chiral HPLC** t_R_ (*S*)=15.8 min; t_R_ (*R*)=17.0 min. ^**1**^ **H NMR** (300 MHz, d‐CHCl_3_): δ 7.28–7.14 (m, 5H), 4.28–4.20 (m, 1H), 2.82 (dd, *J*=13.5, 7.1 Hz, 1H), 2.75 (dd, *J*=13.5, 7.1 Hz, 2H), 2.70–2.51 (m, 2H), 2.10 (s, 3H). In accordance with literature.^[13]^

**[(*S*)‐5‐(Benzyloxy)‐4‐hydroxypentan‐2‐one] (3 b)** 207 mg isolated yield (62%), 87% *ee*, colorless oil. **Chiral HPLC** t_R_ (*R*)=16.2 min; t_R_ (*S*)=19.5 min. ^**1**^ **H NMR** (300 MHz, d_4_‐MeOH): δ 7.38–7.25 (m, 5H), 4.55 (d, *J*=1.9 Hz, 2H), 4.28–4.23 (m, 1H), 3.50–3.40 (m, 2H), 2.72–2.58 (m, 2H), 2.17 (s, 3H). In accordance with literature.^[42]^

**[Benzyl (*R*)‐(3‐hydroxy‐5‐oxohexyl)carbamate] (3 c)** 297 mg isolated yield (70%), 93% *ee*, slightly yellow oil. **Chiral HPLC** t_R_ (*R*)=20.6 min; t_R_ (*S*)=24.7 min. ^**1**^ **H‐NMR** (300 MHz, d_4_‐MeOH) δ 7.38–7.28 (m, 5H), 5.08 (s, 2H), 4.14–4.06 (m, 1H), 3.27 –3.22 (m, 2H), 2.60 (d, *J*=5.9 Hz, 2H), 2.17 (s, 3H), 1.73–1.53 (m, 2H). In accordance with literature.^[15]^

**(3*S*,4*R*)‐3,4‐Dihydroxy‐5‐phenylpentan‐2‐one (4 a)** 84 mg isolated yield (27%), 80% *de*, white solid. ^**1**^ **H NMR** (300 MHz, d_4_‐MeOH): δ 7.29–7.18 (m, 5H), 4.18–4.14 (m, 1H), 3.93 (d, *J*=1.9 Hz, 1H), 2.96 (dd, *J*=13.3, 7.2 Hz, 1H), 2.85 (dd, *J*=13.3, 7.2 Hz), 2.18 (s, 3H). In accordance with literature.^[43]^

**(3*S*,4*R*)‐5‐(Benzyloxy)‐3,4‐dihydroxypentan‐2‐one (4 b)** 169 mg isolated yield (47%), 90% *de*, colorless liquid. ^**1**^ **H NMR** (300 MHz, d‐CHCl_3_): δ 7.37–7.30 (m, 5H), 4.57 (s, 2H), 4.25–4.23 (m, 1H), 4.21–4.19 (m, 1H), 3.64–3.62 (m, 2H), 2.28 (s, 3H). In accordance with literature.^[43]^

**Benzyl ((3*R*,4*S*)‐3,4‐dihydroxy‐5‐oxohexyl)carbamate] (4 c)** 43 mg isolated yield (15%), 92% *de*, colorless liquid. . ^**1**^ **H NMR** (300 MHz, d_4_‐MeOH): δ 7.37–7.27 (m, 5H), 5.07 (s, 2H), 4.05–3.97 (m, 2H), 3.26 (td, *J*=6.8, 4.4 Hz, 2H), 2.20 (s, 3H), 1.77 (q, *J*=6.8 Hz, 2H). In accordance with literature.^[50]^

**(*R*)‐2‐((*R*)‐1‐Hydroxy‐2‐phenylethyl)cyclobutan‐1‐one (5 a)** 47 mg isolated yield (15%), 80% *de*, colorless liquid. ^**1**^ **H NMR** (300 MHz, d‐CHCl_3_): Major: δ 7.34–7.21 (m, 5H), 4.02 (q, *J*=6.5 Hz, 1H), 3.43–3.35 (m, 1H), 3.04–2.94 (m, 2H), 2.88 (d, *J*=6.5 Hz, 2H), 2.10–2.07 (m, 2H), 1.90–1.86 (m, 1H). Minor: δ 7.34–7.21 (m, 5H), 4.21 (dt, *J*=8.0, 5.1 Hz, 1H), 3.70–3.62 (m, 1H), 310–3.06 (m, 2H), 2.88 (d, *J*=6.5 Hz, 2H), 2.10–2.07 (m, 2H), 1.90–1.86 (m, 1H). ^**13**^ **C NMR** (75 MHz, d‐CHCl_3_): Major: 211.3, 137.6, 129.6, 128.7, 126.8, 72.4, 64.7, 45.5, 41.7, 14.1. Minor: δ 211.4, 137.6, 129.5, 128.8, 126.9, 70.2, 65.3, 45.3, 33.9, 12.3. **HR‐MS** [M+H] 191.106807 calc. 191.106656.

**(*R*)‐2‐((*S*)‐2‐(Benzyloxy)‐1‐hydroxyethyl)cyclobutan‐1‐one (5 b)** 76 mg isolated yield (21%), diasteromeric ratio 50:50, colorless liquid. ^**1**^ **H NMR** (300 MHz, d_4_‐MeOH): Major: δ 7.34–7.26 (m, 5H), 4.53 (s, 2H), 3.92 (dt, *J*=6.5, 5.0 Hz, 1H), 3.57 (dd, *J*=9.8, 6.6 Hz, 1H), 3.55–3.53 (m, 2H), 2.95 (ddd, *J*=10.1, 7.7, 2.7 Hz, 1H), 2.89 (ddd, *J*=9.8, 6.2, 2.5 Hz, 1H), 2.09–2.02 (m, 2H). Minor: δ 7.34–7.26 (m, 5H), 4.53 (s, 2H), 4.05 (dt, *J*=6.3, 5.0 Hz, 1H), 3.47 (dd, *J*=9.0, 5.2, 1H), 3.40 (dd, *J*=9.8, 6.3, 1H), 3.05–2.81 (m, 2H), 2.15–1.96 (m, 2H). In accordance with literature.^[42]^

**Benzyl ((*R*)‐3‐hydroxy‐3‐((*R*)‐2‐oxocyclobutyl)propyl)carbamate (5 c)** 21 mg isolated yield (27%), 60% *de*, colorless liquid. ^**1**^ **H NMR** (300 MHz, d_4_‐MeOH): Major: δ 7.37–7.27 (m, 5H), 5.06 (s, 2H), 3.73 (dt, *J*=9.0, 4.5 Hz, 1H), 3.45–3.35 (m, 1H), 3.22 (td, *J*=6.8, 2.8 Hz, 2H), 2.98–2.81 (m, 2H), 2.07 (qd, *J*=10.1, 6.2 Hz, 1H), 2.02–1.90 (m, 1H), 1.86–1.64 (m, 2H). Minor: δ 7.37–7.37 (m, 5H), 5.06 (s, 2H), 3.93–3.87 (m, 1H), 3.45–3.35 (m, 1H), 3.25–3.18 (m, 2H), 3.02–2.95 (m, 2H), 2.11–1.93 (m, 2H), 1.81–1.58 (m, 2H). In accordance with literature.^[15]^

***anti*** **‐4‐Amino‐1‐phenylpentan‐2‐ol (** *anti*‐**6 a)** 64 mg isolated yield (74%), >97% *de*, yellow liquid. ^**1**^ **H NMR** (300 MHz, d‐CHCl_3_): δ 7.31–7.17 (m, 5H), 4.17–4.13 (m, 1H), 3.38 (tt, *J*=6.6, 3.6 Hz, 1H), 2.84 (dd, *J*=13.4, 7.1 Hz, 1H), 2.70 (dd, *J*=13.4, 6.2 Hz, 1H), 1.50 (qdd, *J*=14.3, 7.6, 3.3 Hz, 2H), 1.13 (d, *J*=6.5 Hz, 3H). δ ^**13**^ **C NMR** (75 MHz, d‐CHCl_3_): δ 139.1, 129.5, 128.5, 126.3, 70.4, 44.7, 44.4, 42.0, 23.4. In accordance with literature.^[13]^ **HR‐MS**: [M+H] 180.138429 calc. 180.138291. [α]_D_^20^=–7.9 (c=1.0, CHCl_3_).

***syn*** **‐4‐Amino‐1‐phenylpentan‐2‐ol (** *syn*‐**6 a)** 36 mg isolated yield (71%), 91% *de*, yellow liquid. ^**1**^ **H NMR** (300 MHz, d_4_‐MeOH): Major: δ 7.30–7.15 (m, 5H), 3.91 (tt, *J*=6.5 Hz, 3.6 Hz, 1H), 3.10 (qt, *J*=6.5 Hz, 3.5, 1H), 2.80–2.66 (m, 2H), 1.47 (t, *J*=6.4 Hz, 2H), 1.07 (d, *J*=6.4 Hz, 3H). Minor: δ 7.30–7.15 (m, 5H), 3.77–3.69 (m, 1H), 3.21–3.15 (m, 1H), 2.90–2.82 (m, 2H), 1.68–1.52 (m 2H), 1.10 (d, *J*=6.6, 3H). ^**13**^ **C NMR** (75 MHz, d_4_‐MeOH): Major: δ 140.0, 130.6, 129.3, 127.2, 72.8, 46.8, 45.9, 45.3, 22.8. Minor: δ 140.0, 130.7, 129.4, 127.2, 72.8, 46.7, 45.9, 45.3, 22.7. δ [α]_D_^20^=+3.4 (c=1.0, CHCl_3_)

***anti*** **‐4‐Amino‐1‐(benzyloxy)pentan‐2‐ol** (*anti*‐**6 b)** 63 mg isolated yield (60%), 88% *de*, reddish‐brown liquid ^**1**^ **H NMR** (300 MHz, d‐CHCl_3_): Major: δ 7.37–7.27 (m, 5H), 4.56 (s, 2H), 4.12–4.05 (m, 1H), 3.45–3.43 (m, 2H), 3.30 (d, *J*=3.5 Hz, 1H), 3.02 (s, 2H), 1.64 (ddd, *J*=14.3, 8.7, 3.5 Hz, 1H), 1.44 (ddd, *J*=14.3, 7.7, 3.2 Hz, 1H), 1.15 (d, *J*=6.5 Hz, 3H). Minor: δ ^**13**^ **C NMR** (75 MHz, d‐CHCl_3_): Major: δ 138.2, 128.4, 127.7, 127.7, 74.5, 73.3, 67.9, 44.3, 39.7, 23.6. Minor: δ 138.2, 128.4, 127.7, 127.7, 74.5, 73.3, 67.9, 46.6, 40.1, 23.6. **HR‐MS**: [M+H] 210.149227 calc. 210.148855. [α]_D_^20^=−17.9 (c=1.2, CHCl_3_).

***syn*** **‐Amino‐1‐(benzyloxy)pentan‐2‐ol (** *syn‐* **6 b)** 30 mg isolated yield (56%), 79% *de*, yellow liquid ^**1**^ **H NMR** (300 MHz, d_4_‐MeOH): Major: δ 7.37–7.25 (m, 5H), 4.54 (s, 2H), 3.88 (ddd, *J*=9.6, 5.5, 4.4 Hz, 1H), 3.41 (d, *J*=5.4 Hz, 2H), 3.21 (q, *J*=6.5 Hz, 1H), 1.62–1.53 (m, 2H), 1.17 (d, *J*=6.5 Hz, 3H). Minor: δ 7.37–7.25 (m, 5H), 4.52 (s, 2H), 3.83–3.73 (m, 2H), 2.94–2.82 (m, 1H), 1.54–1.48 (m, 1H), 1.38–1.29 (m, 1H), 1.11 (d, *J*=6.5 Hz, 3H). ^**13**^ **C NMR** (75 MHz, d_4_‐MeOH): Major: δ 139.6, 129.4, 128.9, 128.7, 75.9, 74.4, 70.3, 46.9, 41.8, 22.1. Minor: δ 139.6, 129.4, 128.9, 128.8, 76.3, 74.4, 70.3, 46.6, 41.7, 24.6. **HR‐MS**: [M+H] 210.1512 calc. 210.1494. [α]_D_^20^=–2.8 (c=1.0, CHCl_3_)

***anti*** **‐Benzyl‐5‐amino‐3‐hydroxyhexyl)carbamate (** *anti*‐**6 c)** 60 mg isolated yield (45%), 78% *de*, yellow liquid. ^**1**^ **H NMR** (300 MHz, d‐CHCl_3_): Major: δ 7.35–7.31 (m, 5H), 5.60–5.58 (m, 1H), 5.08 (s, 2H), 4.01–3.98 (m, 1H), 3.45–3.22 (m, 7H), 1.63–1.53 (m, 3H), 1.45 (ddd, *J*=14.5, 7.0, 3.0 Hz, 1H), 1.17 (d, *J*=6.5 Hz, 3H). Minor: δ 7.25–7.31 (m, 5H), 5.44 (m, 1H), 5.11(s, 2H), 3.45–3.22 (m, 7H), 4.48–4.40 (m, 1H), 1.63–1.53 (m, 3H), 1.26–1.21 (m, 1H), 1.06 (d, *J*=6.7 Hz, 3H). ^**13**^ **C NMR** (75 MHz, d‐CHCl_3_): Major: δ 156.9, 136.9, 128.6, 128.2, 128.1, 67.7, 66.7, 44.9, 42.5, 38.8, 37.1, 23.0. Minor: δ 156.9, 137.1, 128.5, 128.2, 128.1, 67.1, 66.4, 44.8, 42.3, 40.1, 38.8, 37.0, 22.8. **HR‐MS**: [M+H] 267.170755 calc. 267.170319. [α]_D_^20^=−1.5 (c=1.1 g/100 mL, CHCl_3_)

***syn*** **‐Benzyl‐5‐amino‐3‐hydroxyhexyl)carbamate** (*syn*‐**6 c)** 47 mg isolated yield (70%), >97% *de*, yellow liquid. ^**1**^ **H NMR** (300 MHz, d_4_‐MeOH): δ 7.35–7.26 (m, 5H), 5.07 (s, 2H), 3.73 (tt, *J*=8.4, 4.5 Hz, 1H), 3.23 (t, *J*=7.0 Hz, 2H), 3.09 (qt, *J*=6.5, 5.8 Hz, 1H), 1.70–1.54 (m, 2H), 1.52–1.45 (m, 2H), 1.11 (d, *J*=6.4 Hz, 3H). ^**13**^ **C NMR** (75 MHz, d_4_‐MeOH): δ 159.0, 138.5, 129.5, 129.0, 128.8, 69.1, 67.4, 46.7, 46.3, 39.1, 38.6, 23.1. **HR‐MS**: [M+H] 267.1736 calc. 267.1709. [α]_D_^20^=+17.8 (c=4.2, CHCl_3_).

**(2*R*,3*R*,4*R*)‐4‐Amino‐1‐phenylpentane‐2,3‐diol** (**7 a)** 31 mg isolated yield (62%), 58% *de*. yellow liquid. ^**1**^ **H NMR** (300 MHz, d_4_‐MeOH): Major: δ 7.30–7.15 (m, 5H), 3.94 (ddd, *J*=8.3, 6.2, 2.6 Hz, 1H), 3.16 (dd, *J*=6.4, 2.6 Hz, 1H), 3.04 (q, *J*=6.5 Hz, 1H), 2.91–2.76 (m, 2H), 1.13 (d, *J*=6.5 Hz, 3H). Minor: δ 7.30–7.15 (m, 5H), 3.62 (td, *J*=8.9, 2.6 Hz, 1H), 3.21–3.19 (m, 1H), 3.04 (q, *J*=6.5 Hz, 1H), 2.61–2.54 (m, 2H), 1.11 (d, *J*=6.5 Hz, 3H). ^**13**^ **C NMR** (75 MHz, d_4_‐MeOH) Major: δ 140.4, 130.5, 129.3, 127.1, 76.9, 73.5, 50.2, 41.2, 18.8. Minor: δ 140.7, 130.7, 129.1, 127.0, 78.0, 75.2, 41.6, 16.4. **HR‐MS**: [M+H] 196.133385 calc. 196.133205. [α]_D_^20^=−11.4 (c=2.8, CHCl_3_).

**(2*R*,3*R*,4*R*)‐4‐Amino‐1‐(benzyloxy)pentane‐2,3‐diol** (**7 b)** 80 mg isolated yield (71%), 82% *de*, yellow liquid. ^**1**^ **H NMR** (300 MHz, d_4_‐MeOH): Major: δ 7.38–7.27 (m, 5H), 4.55 (s, 2H), 3.91 (ddd, *J*=6.2, 5.3, 3.1 Hz, 1H), 3.63–3.52 (m, 2H), 3.40 (dd, *J*=6.0, 3.1 Hz, 1H), 3.11–3.01 (m, 1H), 1.16 (d, *J*=6.6 Hz, 3H). Minor: δ 7.31–7.24 (m, 5H), 4.54 (s, 2H), 4.03–3.98 (m, 2H), 3.75–3.65 (m, 1H), 3.22–3.16 (m, 1H), 1.21 (d, *J*=6.5 Hz, 3H). ^**13**^ **C NMR** (75 MHz, d_4_‐MeOH): Major: δ 139.6, 129.4, 128.9, 128.7, 75.6, 74.4, 73.1, 71.1, 50.1, 18.3. Minor: δ 140.8, 129.4, 129.0, 128.7, 75.7, 74.4, 73.1, 71.1, 50.1, 18.3. **HR‐MS**: [M+H] 226.144023 calc. 226.14377. [α]_D_^20^=−2.3 (c=4.4, CHCl_3_)

**Benzyl ((3*R*,4*R*,5*R*)‐5‐amino‐3,4‐dihydroxyhexyl)carbamate** (**7 c)** 45 mg isolated yield (32%), 82% *de*, yellow liquid. ^**1**^ **H NMR** (300 MHz, d_4_‐MeOH): Major: δ 7.35–7.28 (m, 5H), 5.07 (s, 2H), 3.72 (ddd, *J*=8.3, 5.4, 3.4 Hz, 1H), 3.26 (t, *J*=7.0 Hz, 2H), 3.17 (dd, *J*=6.1, 3.3 Hz, 1H), 3.00 (p, *J*=6.4 Hz, 1H), 1.73–1.65 (m, 2H), 1.12 (d, *J*=6.7 Hz, 3H). Minor: 7.35–7.28 (m, 5H), 5.09 (s, 2H), 3.92–3.75 (m, 1H), 3.63–3.54 (m, 1H), 3.50–3.44 (m, 1H), 2.74–2.65 (m, 1H), 2.15–1.98 (m, 2H), 1.09 (d, *J*=6.7 Hz, 3H). δ ^**13**^ **C NMR** (75 MHz, d_4_‐MeOH): Major: δ 159.0, 138.5, 129.5, 129.0, 128.8, 78.1, 69.9, 67.4, 49.8, 38.8, 34.7, 18.5. Minor: δ 159.0, 138.5, 129.5, 129.0, 128.8, 78.1, 69.9, 67.4, 49.8, 38.8, 34.7, 18.5. **HR‐MS**: [M+H] 283.165831 calc. 283.165234. [α]_D_^20^=−4.0 (c=1.7, CHCl_3_).

**(*S*)‐1‐((1*R*,2*R*)‐2‐Aminocyclobutyl)‐2‐phenylethan‐1‐ol** (**8 a)** 19 mg isolated yield (20%), 87% *de*, yellow liquid. ^**1**^ **H NMR** (300 MHz, d_4_‐MeOH): Major: δ 7.30–7.16 (m, 5H), 4.06 (ddd, *J*=9.8, 7.8, 4.1 Hz, 1H), 3.66 (tdd, *J*=7.8, 4.1, 1.2 Hz, 1H), 2.79 (dd, *J*=13.8, 4.1 Hz, 1H), 2.58 (dd, *J*=13.8, 7.8 Hz, 1H), 2.43–2.26 (m, 2H), 1.93–1.84 (m, 2H), 1.82–1.76 (m, 1H). Minor: δ 7.22–7.15 (m, 5H), 3.98–3.95 (m 1H), 3.81–3.77 (m, 1H), 2.73–2.71 (m, 1H), 2.69–2.68 (m, 1H), 2.57–2.52 (m, 1H), 2.04–2.00 (m, 2H) 1.87–1.85 (m, 1H). ^**13**^ **C NMR** (75 MHz, d_4_‐MeOH): Major: δ 140.1, 130.7, 129.2, 127.1, 73.7, 49.8, 45.1, 42.3, 27.7, 21.0. Minor: 140.1, 130.8. 129, 2 127.1, 73.7, 50.9, 44.8, 42.6, 26.9, 20.4. δ **HR‐MS**: [M+H] 192.13838 calc. 192.138291. [α]_D_^20^=+1.4 (c=1.0, CHCl_3_)

**(*S*)‐1‐((1*S*,2*R*)‐2‐Aminocyclobutyl)‐2‐(benzyloxy)ethan‐1‐ol (8 b)** 22 mg isolated yield (19%), 66% *de*, yellow liquid. ^**1**^ **H NMR** (300 MHz, d_4_‐MeOH): δ 1H NMR (300 MHz, d_4_‐MeOH) δ 7.36–7.27 (m, 5H), 4.55–4.51 (m, 2H), 4.06–4.00 (m, 1H), 3.68 (ddd, *J*=8.5, 6.5, 5.2 Hz, 1H), 3.52–3.37 (m, 2H), 2.53–2.47 (m, 1H), 2.29–2.25 (m, 1H), 1.80 (ddd, *J*=10.3, 4.7, 2.1 Hz, 2H). Minor: δ 7.31–7.24 (m, 5H), 4.50–4.46 (m, 2H), 4.24–4.11 (m, 1H), 3.97–3.91 (m, 1H), 3.87–3.78 (m, 2H), 2.61–2.58 (m, 1H), 2.15–2.01 (m, 1H), 1.94–1.85 (m, 2H). ^**13**^ **C NMR** (75 MHz, d_4_‐MeOH): δ 139.6, 129.4, 128.9, 128.7, 74.3, 74.1, 71.9, 49.9, 42.3, 28.1, 20.4. Minor: 139.6, 129.3, 128.8, 128.6, 74.4, 74.1, 71.9, 50.3, 40.3, 28.1, 20.5. δ **HR‐MS**: [M+H] 222.149103 calc. 222.148855. [α]_D_^20^=+5.8 (c=1.0, CHCl_3_)

## Supporting information

As a service to our authors and readers, this journal provides supporting information supplied by the authors. Such materials are peer reviewed and may be re‐organized for online delivery, but are not copy‐edited or typeset. Technical support issues arising from supporting information (other than missing files) should be addressed to the authors.

Supporting Information
