# Supplementary material for: Biocatalytic Transamination of Aldolase‐Derived 3‐Hydroxy Ketones
Source: Adv Synth Catal. 2023 Apr 26;365(9):1485–95. doi: 10.1002/adsc.202300201 (PMC10952931; doi:10.1002/adsc.202300201)
Supplement: Supplementary file 1 — Supporting Information [file ADSC-365-1485-s001.pdf]

Advanced Synthesis & Catalysis

Supporting Information

Biocatalytic Transamination of Aldolase-Derived 3-Hydroxy Ketones

Mathias Pickl, Markus Ebner, Samantha Gittings, Pere Clapés,* and Wolfgang Kroutil*© 2023 The Authors. Advanced Synthesis & Catalysis published by Wiley-VCH GmbH. This is an open access article under the terms of the Creative Commons Attribution License, which permits use, distribution and reproduction in any medium, provided the original work is properly cited.

Supporting Information
Biocatalytic trans-amination of aldolase derived 3-hydroxy ketones

Mathias Pickl†‡, Markus Ebner‡, Samantha Gittings#, Pere Clapés†*, Wolfgang Kroutil‡*

†Department of Chemical Biology. Instituto de Química Avanzada de Cataluña (IQAC−CSIC), Jordi Girona 18-26, 08034 Barcelona, Spain.

‡Institute of Chemistry, University of Graz, Heinrichstrasse 28, 8010, Graz, Austria.

#Prozomix Ltd. West End Industrial Estate, Haltwhistle, Northumberland NE49 9HA, U.K.

Corresponding author: pere.clapes@iqac.csic.es and wolfgang.kroutil@uni-graz.at

Table of Content

1. Biotransformations **3**

1.1. TA Screening for aldol product 3a-c **3**

1.2. Time course of aldol addition and yields **5**

1.3. Determination of transaminase activity **6**

1.4. One pot setup of aldol-transamination **6**

1.5. Synthesis of racemic 3a-c **8**

2. Analytics **9**

2.1. Derivatization of GC-MS samples **9**

2.2. GC-MS chromatograms **9**

2.3. CSP-HPLC chromatograms **10**

2.4. Reaction monitoring HPLC chromatograms **12**

2.5. HPLC-UV Calibration **14**

3. NMR **15**

3.1. Identification of relative configurations **12**

4. References **48**

1. Biotransformations

1.1. TA Screening for aldol product 3a-c

Table S1. Screening of TA-Kit for the transamination of model 1,3-hydroxyketones **3a-c** measured by HPLC-UV. Conversion to aldol adduct (%) at 24 h was determined by HPLC analysis using the external standard methodology. Conditions: CFE (5 mg), **3-5** (25 mM), D- or L-alanine (375 mM), PLP (1 mM), Kp_i buffer (50 mM, pH 7), total volume 200 μL, rt, horizontal shaking, 24h. Amine product: light green: conv. <20%, green: conv. 20-60% dark green: conv. >60%.

3a

1	2	3	4	5	6	7	8	9	10	11	12
13	14	15	16	17	18	19	20	21	22	23	24
25	26	27	28	29	30	31	32	33	34	35	36
37	38	39	40	41	42	43	44	45	46	47	48
49	50	51	52	53	54	55	56	57	58	59	60
61	62	63	64	65	66	67	68	69	70	71	72
73	74	75	76	77	78	79	80	81	82	83	84
85	86	87	88	89	90	91	92	93	94	95	96
97	98	99	100	101	102	103	104	105	106	107	108
109	110	111	112	113	114	115	116	117	118	119	120
121	122	123	124	125	126	127	128	129	130	131	132
133	134	135	136	137	138	139	140	141	142	143	144
145	146	147	148	149	150	151	152	153	154	155	156
157	158	159	160	161	162	163	164	165	166	167	168
169	170	171	172	173	174	175	176	177	178	179	180
181	182	183	184	185	186	187	188	189	190	191	192

3b

1	2	3	4	5	6	7	8	9	10	11	12
13	14	15	16	17	18	19	20	21	22	23	24
25	26	27	28	29	30	31	32	33	34	35	36
37	38	39	40	41	42	43	44	45	46	47	48
49	50	51	52	53	54	55	56	57	58	59	60
61	62	63	64	65	66	67	68	69	70	71	72
73	74	75	76	77	78	79	80	81	82	83	84
85	86	87	88	89	90	91	92	93	94	95	96
97	98	99	100	101	102	103	104	105	106	107	108
109	110	111	112	113	114	115	116	117	118	119	120

121	122	123	124	125	126	127	128	129	130	131	132
133	134	135	136	137	138	139	140	141	142	143	144
145	146	147	148	149	150	151	152	153	154	155	156
157	158	159	160	161	162	163	164	165	166	167	168
169	170	171	172	173	174	175	176	177	178	179	180
181	182	183	184	185	186	187	188	189	190	191	192

3c

1	2	3	4	5	6	7	8	9	10	11	12
13	14	15	16	17	18	19	20	21	22	23	24
25	26	27	28	29	30	31	32	33	34	35	36
37	38	39	40	41	42	43	44	45	46	47	48
49	50	51	52	53	54	55	56	57	58	59	60
61	62	63	64	65	66	67	68	69	70	71	72
73	74	75	76	77	78	79	80	81	82	83	84
85	86	87	88	89	90	91	92	93	94	95	96
97	98	99	100	101	102	103	104	105	106	107	108
109	110	111	112	113	114	115	116	117	118	119	120
121	122	123	124	125	126	127	128	129	130	131	132
133	134	135	136	137	138	139	140	141	142	143	144
145	146	147	148	149	150	151	152	153	154	155	156
157	158	159	160	161	162	163	164	165	166	167	168
169	170	171	172	173	174	175	176	177	178	179	180
181	182	183	184	185	186	187	188	189	190	191	192

1.2. Time course experiment of aldol addition and yields from preparative scale experiments

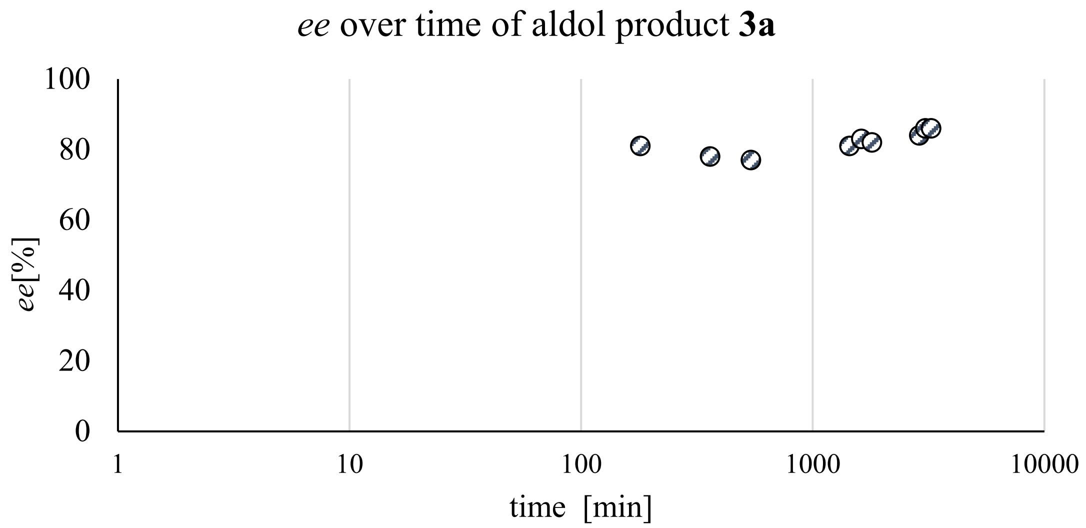

Figure S1. Time course experiments of change in *ee* in the formation of aldol product **3a**.

Table S2. Synthesis of aldol products using *Ec*FSA variants.

Aldol intermediate	Catalyst	Yield
**3a**	FSA D6N	137 mg (48%), 95% *ee*
**3b**	FSA D6N	207 mg (62%), 87% *ee*
**3c**	FSA D6N	297 mg (70 %), 93% *ee*
**4a**	Wild type FSA	84 mg (27%) 80% *de*
**4b**	Wild type FSA	169 mg (47%) 90% *de*
**4c**	Wild type FSA	43 mg (15 %) 92% *de*
**5a**	FSA D6N	47 mg (15 %) 80% *de*
**5b**	FSA D6N	76 mg (21%) ratio 50:50
**5c**	FSA D6N	21 mg (27 %) 60% *de*

1.3. Determination of transaminase activity

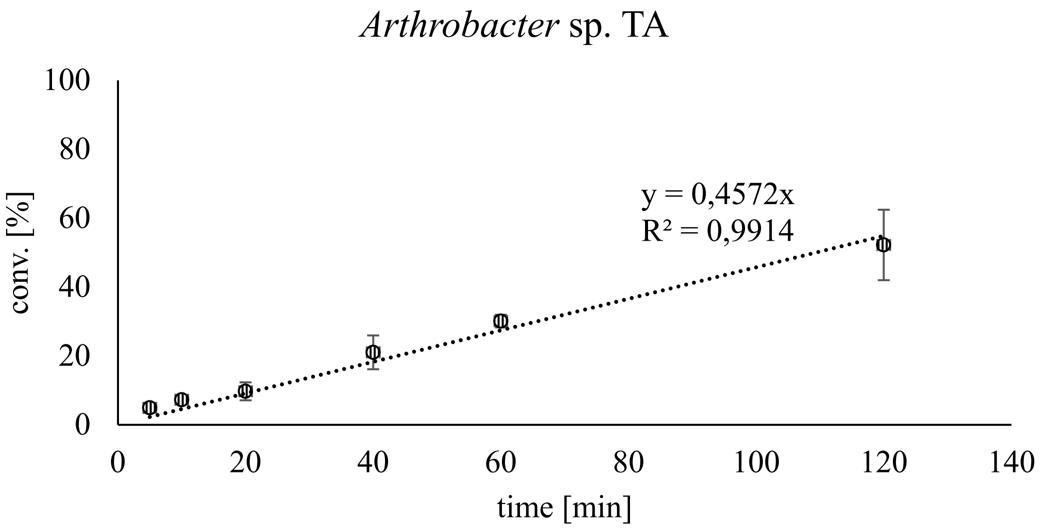

Figure S2. Expemplary initial rate plot of *Arthrobacter* sp. TA lyophilized whole cell preparation with model substrate **3a**.

1.4. One pot setup of aldol-transamination

Lyophilized cells of *E. coli* BL21 (DE3) containing overexpressed TA (20 mg) were rehydrated in an microcentrifuge tube (1.5 mL) in TEoA buffer (300 µL total, 50 mM, pH 8, 1 mM PLP, 1 mM NAD^+) for 30 min at 30°C and 120 rpm on an orbital shaker. **I)** The rehydrated cells were added to *Ec*FSA D6N (3 mg, 135 U) in TEoA buffer (1 mL total, 50 mM, pH 8). To this solution, aldehyde **1a** (50 mM final concentration) dissolved in **2a** (45 µL) was added. Then, L-alanine (125 mM), D-glucose (150 mM), LDH (10 U), and GDH (30 U) were added. The mixture was shaken at 25°C and 700 rpm. The reaction was stopped after 24 h and analyzed GC-MS after derivatization. **II)** *Ec*FSA (3 mg, 135 U) was in TEoA buffer (300 µL total, 50 mM, pH 8). To this solution, aldehyde **1a** (50 mM final concentration) dissolved in **2a** (45 µL) was added. The reaction was stopped after 24 h, *Ec*FSA D6N via heatshock (95°C, 5 min) removed by centrifugation. Then, the rehydrated cells, L-alanine (125 mM), D-glucose (150 mM), LDH (10 U) and GDH (30 U) were added and the volume adjusted with TEoA buffer (1 mL total, 50 mM, pH 8). The mixture was shaken at 25°C and 700 rpm. The reaction was

stopped after additional 24 h and analyzed GC-MS after derivatization. **III)** *Ec*FSA (3 mg, 135 U) was in TEoA buffer (300 µL total, 50 mM, pH 8). To this solution, aldehyde **1a** (50 mM final concentration) dissolved in **2a** (45 µL) was added. Then, after 24 h shaking at 25 °C with 700 rpm the rehydrated cells were added. Then, L-alanine (375 mM) was added and the volume adjusted with TEoA buffer (1 mL total, 50 mM, pH 8). The mixture was shaken at 25°C and 700 rpm. The reaction was stopped after additional 24 h and analyzed GC-MS after derivatization. **IV)** *Ec*FSA (3 mg, 135 U) was in TEoA buffer (300 µL total, 50 mM, pH 8). To this solution, aldehyde **1a** (50 mM final concentration) dissolved in **2a** (45 µL) was added. The reaction was stopped after 24 h. Then, the rehydrated cells were added. Then, L-alanine (125 mM), D-glucose (150 mM), LDH (10 U), and GDH (30 U) were added and the volume adjusted with TEoA buffer (1 mL total, 50 mM, pH 8). The mixture was shaken at 25°C and 700 rpm. The reaction was stopped after additional 24 h and analyzed GC-MS after derivatization. **V)** *Ec*FSA (3 mg, 135 U) was in TEoA buffer (300 µL total, 50 mM, pH 8). To this solution, aldehyde **1a** (50 mM final concentration) dissolved in **2a** (45 µL) was added. The reaction was stopped after 24 h, *Ec*FSA D6N via heatshock (95°C, 5 min) und subsequent cooling removed by centrifugation. **2a** was removed via airflow and 45 µL plain water were added. Then, the rehydrated cells were added. Then, L-alanine (125 mM), D-glucose (150 mM), LDH (10 U), and GDH (30 U) were added and the volume adjusted with TEoA buffer (1 mL total, 50 mM, pH 8). The mixture was shaken at 25°C and 700 rpm. The reaction was stopped after additional 24 h and analyzed GC-MS after derivatization. **IV)** *Ec*FSA (3 mg, 135 U) was in TEoA buffer (300 µL total, 50 mM, pH 8). To this solution, aldehyde **1a** (50 mM final concentration) dissolved in **2a** (45 µL) was added. The reaction was stopped after 24 h, *Ec*FSA D6N via heatshock (95°C, 5 min) and subsequent cooling removed by centrifugation. Then, L-alanine (125 mM), D-glucose (150 mM), LDH (10 U), and GDH (30 U) were added and the volume adjusted with TEoA buffer (1 mL total, 50 mM, pH 8). The mixture was shaken at 25°C and

700 rpm. The reaction was stopped after additional 24 h and analyzed GC-MS after derivatization.

1.5. Synthesis of racemic 3a-c

The procedure was adapted from Roldán et al.[1]

Lithium diisopropylamide (LDA) (1400 µL of a 1.0 M stock solution in hexane) was added to anhydrous THF (8 mL) in a heat dried Schlenk tube and the solution was cooled down to – 78 ºC. Then acetone (100 µL, 2 mmol) was added and stirred for 20 minutes. After that, the electrophile (**1a-c**) (150 µL, 1 mmol) dissolved in anhydrous THF (2 mL) was added. The reaction was stirred for 2 hours at – 78°C. After that the reaction was left to warm up at room temperature and $NaHCO_3$ (20 mL of a saturated solution) was added. The product was extracted with EtOAc (3 x 15 mL), the organic phases were pooled, dried with anhydrous Na_2SO_4 and evaporated to dryness. Conversion: 60%. The products were used without purification as standards for CSP-HPLC analysis.

2. Analytics

2.1. Derivatization of GC-MS samples

Aqueous samples (150 µl) were withdrawn and NaOH (10% v/v, 10 M in water) was added followed by extraction with ethyl acetate (150 µl). The organic phase was dried over anhydrous Na_2SO_4, centrifuged and subjected to derivatization in glass vials. For this, *N*-Methyl-bis(trifluoroacetamide) (30% v/v, MBTFA) was added and the sample was agitated for 45 min at 60°C prior to injection.

2.2. GC-MS chromatograms

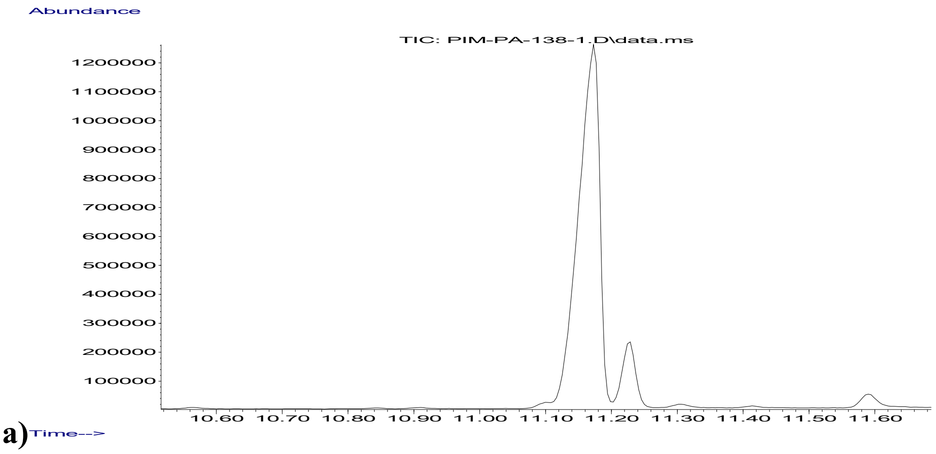

a)

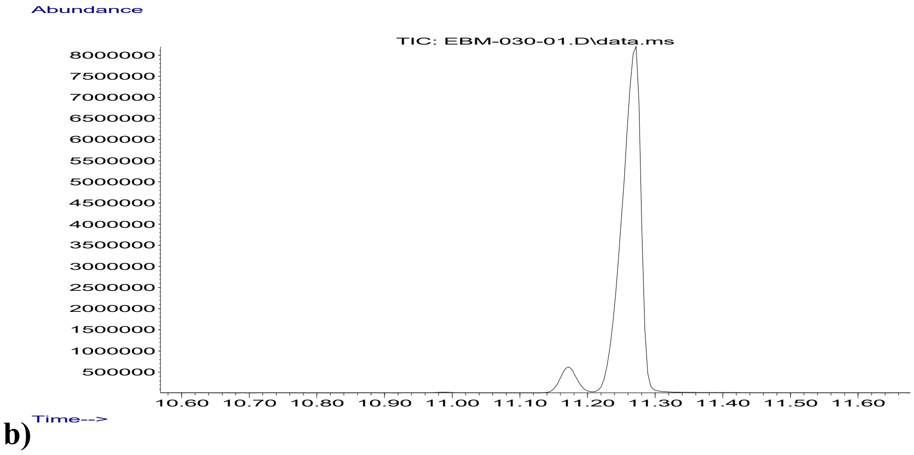

b)

Figure S3. Products obtained by transamination of **3b** with an (*S*)- or (*R*)-TA. In case of (**a**) TA-166 was used, in case of (**b**) *At*TA. In case of (**a**) *syn*- and (**b**) *anti*-diastereomer of derivatized 1,3-amino alcohol **6b**.

2.3. CSP-HPLC chromatograms

3a

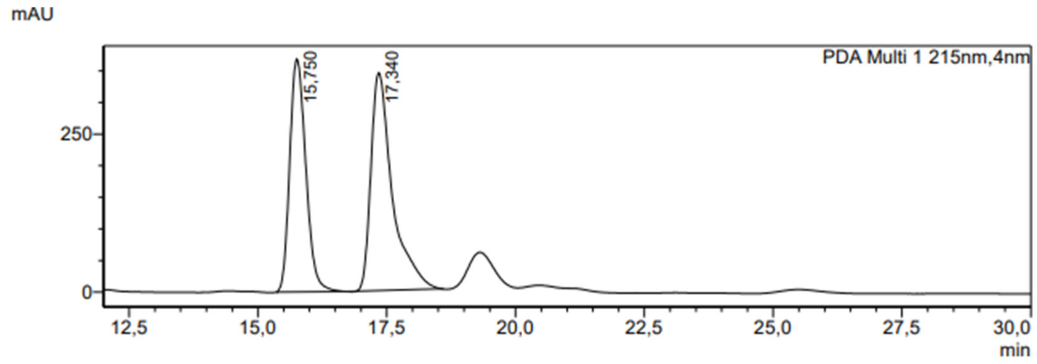

Figure S4. *rac*-4-Hydroxy-5-phenylpentan-2-one (*rac*-**3a**) analyzed on CSP-HPLC.

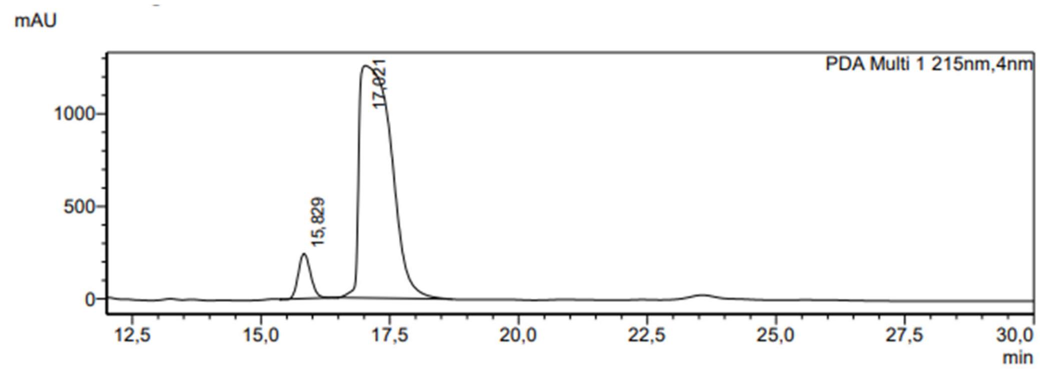

Figure S5. (*R*)-4-Hydroxy-5-phenylpentan-2-one **(3a)** analyzed on CSP-HPLC obtained with *Ec*FSA D6N.

3b

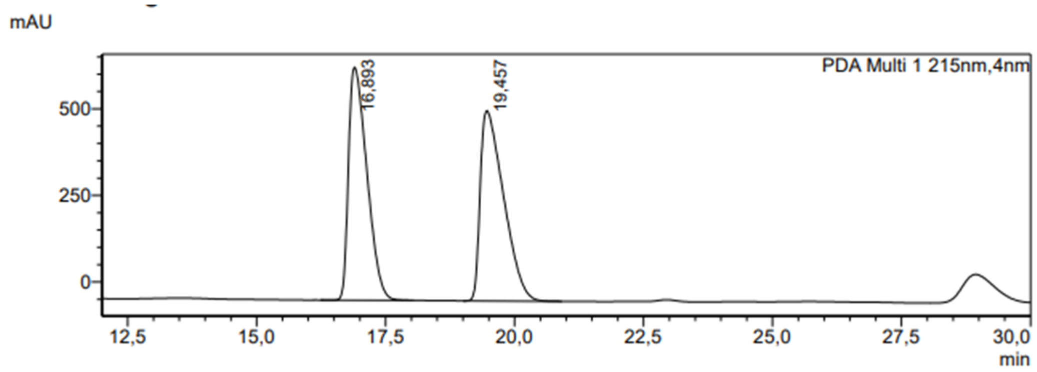

Figure S6. *rac*-5-(Benzyloxy)-4-hydroxypentan-2-one (*rac*-**3b**) analyzed on CSP-HPLC.

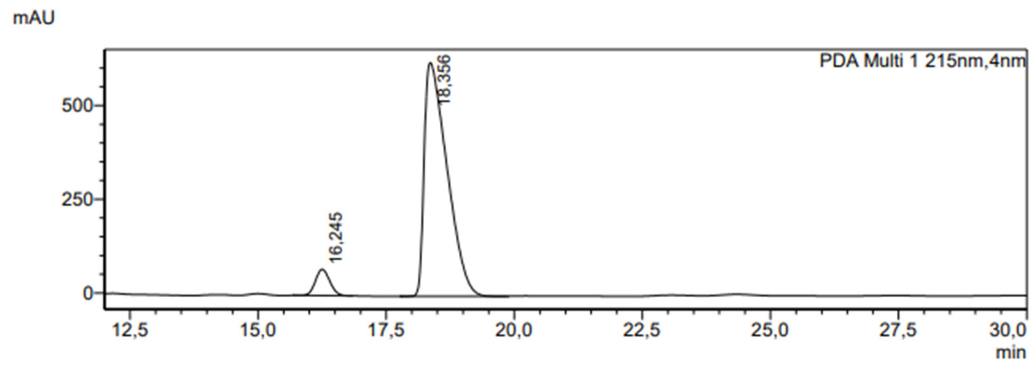

Figure S7. (*S*-5-(Benzyloxy)-4-hydroxypentan-2-one (**3b**) analyzed on CSP-HPLC obtained with *Ec*FSA D6N.

3c

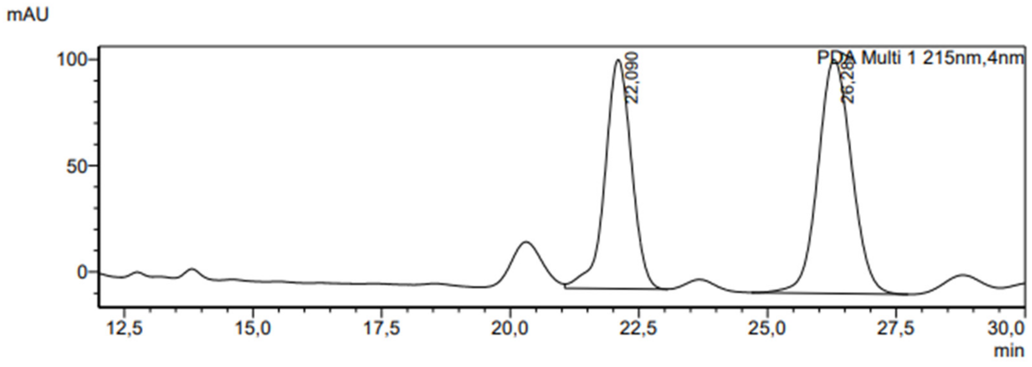

Figure S8. *rac*-*N*-Benzyloxycarbonyl-4-hydroxy-6-(amino)hexan-2-one (*rac*-**3c**) analyzed on CSP-HPLC.

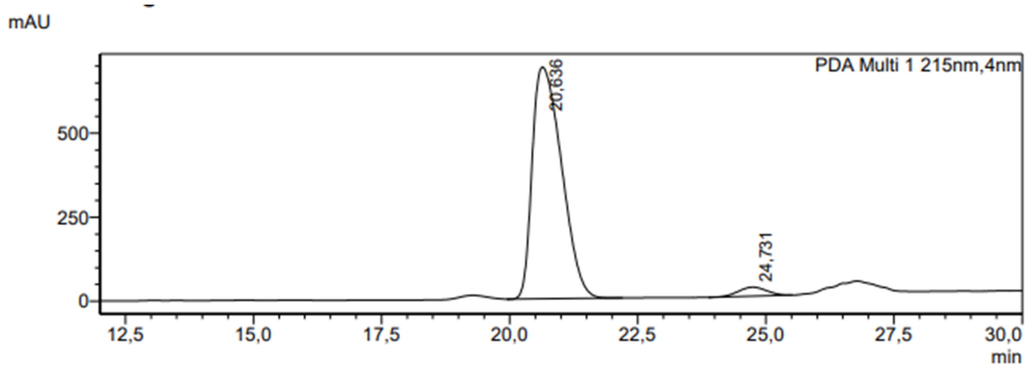

Figure S9. (*R*)-*N*-Benzyloxycarbonyl-4-hydroxy-6-(amino)hexan-2-one (**3c**)] analyzed on CSP-HPLC obtained with *Ec*FSA D6N.

2.4. Reaction monitoring HPLC chromatograms

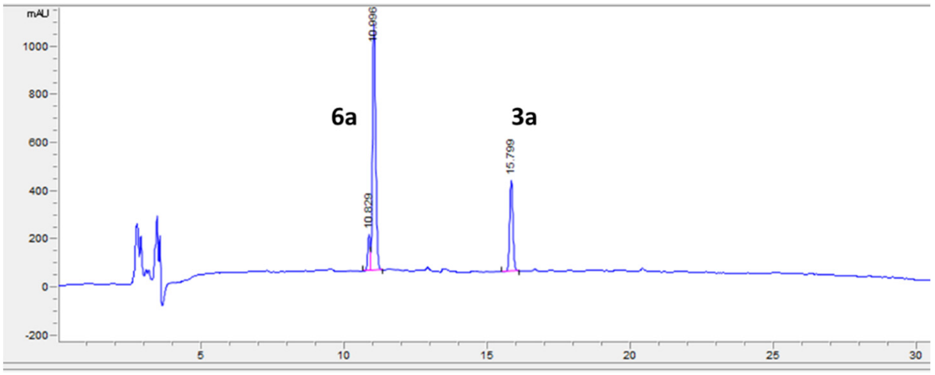

Figure S10. HPLC monitoring trace (210 nm) of transamination of (*R*)-4-hydroxy-5-phenylpentan-2-one (**3a**) obtained with TA166.

rac

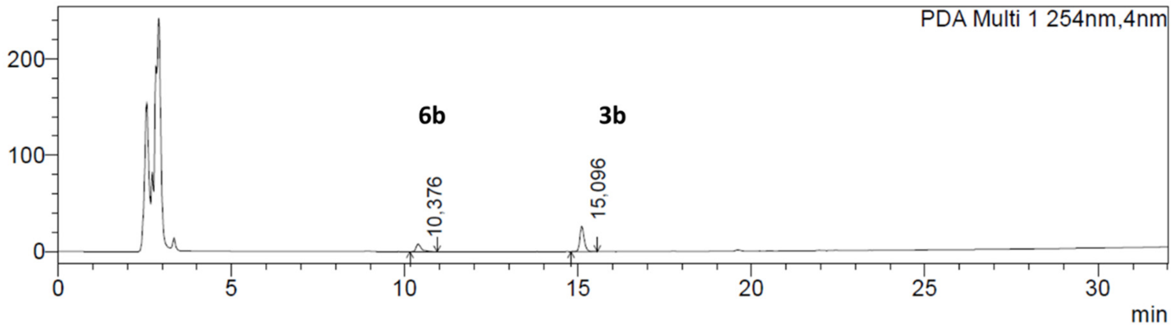

Figure S11. HPLC monitoring trace (254 nm) of transamination of (*S*)-5-(benzyloxy)-4-hydroxypentan-2-one (**3b**) obtained with TA192.

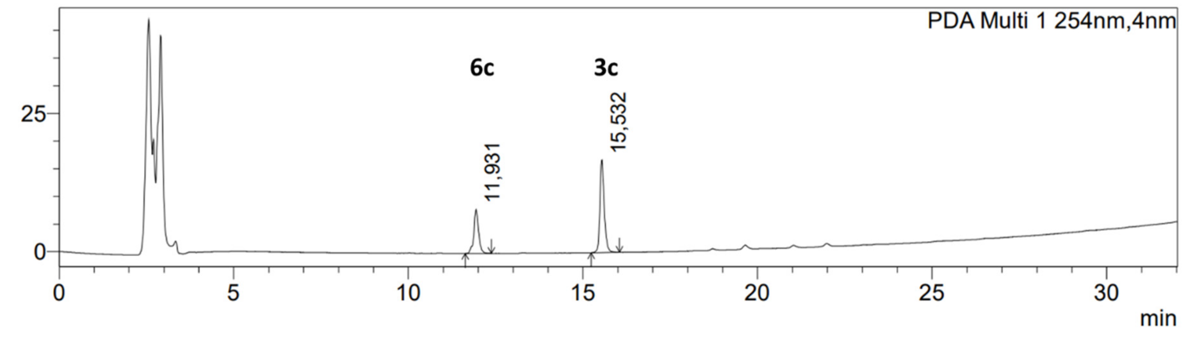

Figure S12. HPLC monitoring trace (254 nm) of transamination of (*R*)-*N*-benzyloxycarbonyl-4-hydroxy-6-(amino)hexan-2-one (**3c**) obtained with TA192.

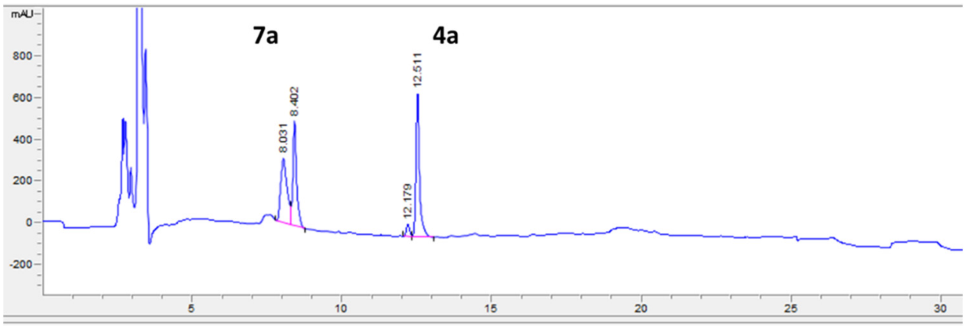

Figure S13. HPLC monitoring trace (210 nm) of transamination of (3*S*,4*R*)-3,4-dihydroxy-5-phenylpentan-2-one (**4a**) obtained with AtTA.

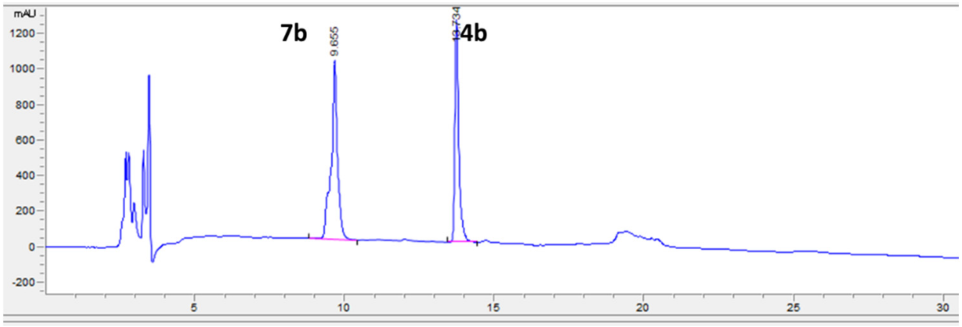

Figure S14. HPLC monitoring trace (210 nm) of transamination of (3*S*,4*R*)-5-(benzyloxy)-3,4-dihydroxypentan-2-one (**4b**) obtained with AtTA.

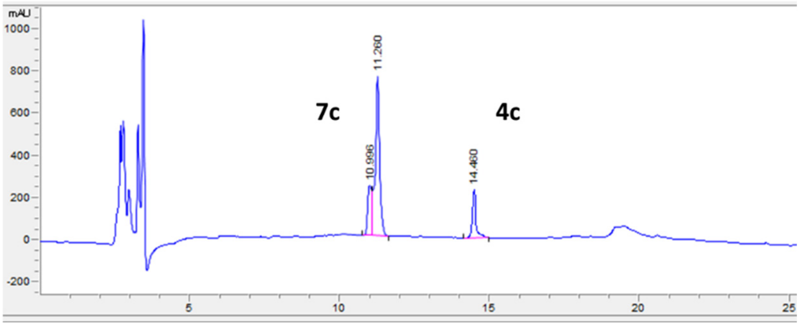

Figure S15. HPLC monitoring trace (210 nm) of transamination of (3*S*,4*R*)-N-benzyloxycarbonyl-3,4-dihydroxy-6-(amino)hexan-2-one (**4c**) obtained with AtTA.

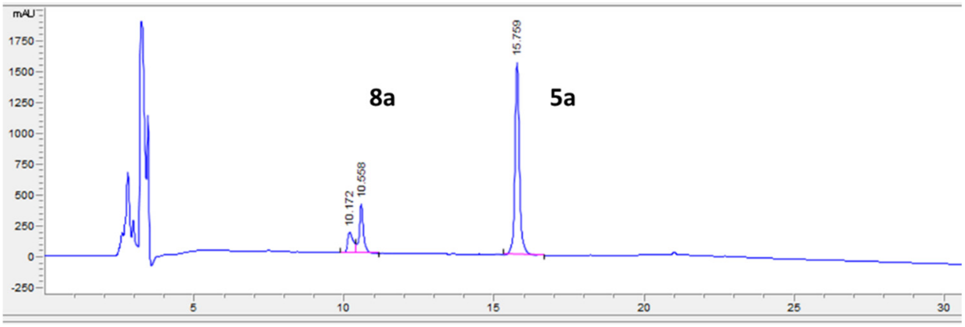

Figure S16. HPLC monitoring trace (210 nm) of transamination of (*R*)-2-((*R*)-1-hydroxy-2-phenylethyl)cyclobutan-1-one (**5a**) obtained with *At*TA.

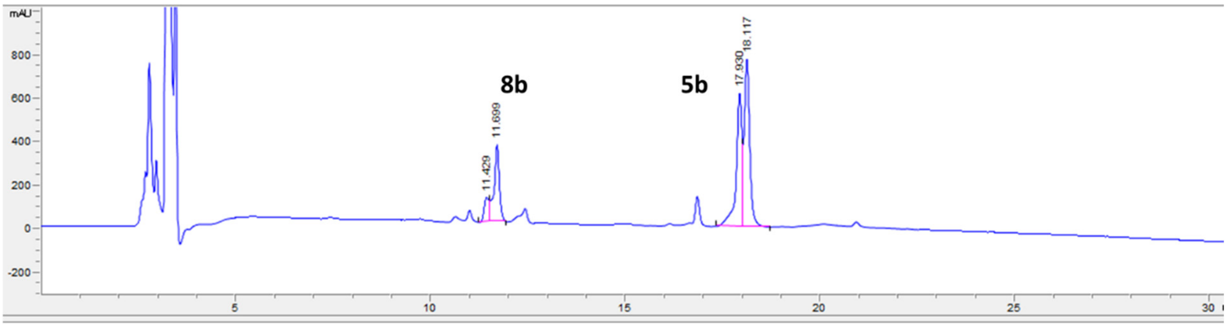

Figure S17. HPLC monitoring trace (210 nm) of transamination of (*R*)-2-((*S*)-2-(benzyloxy)-1-hydroxyethyl)cyclobutan-1-one (**5b)** obtained with *At*TA.

2.5. HPLC-UV Calibration

Table S3. Calibration data of the substrates with HPLC-UV.

	**3a**	**3b**	**3c**	**4a**	**4b**	**4c**	**5a**	**5b**
slope	536	471	238	861	800	606	686	905
intercept	25	200	195	162	1172	502	295	-80
RSQ	1	0.999	0.999	0.995	0.995	0.993	1	0.992

3. NMR

3.1. Identification of relative configurations

The relative configurations and thus the absolute configuration of the amine products was identified with the scalar coupling constants of the diastereotopic H-atoms at C3.[2] The diastereotopic C3 H-atoms split into a doublet of doublet of doublets, which can be seen in an excerpt of the ^{1}H-NMR spectrum of product *anti*-**6a** (Figure S18). The dihedral angle betweenthe coupled protons H2 and H4 determines the coupling constants. As the hydroxy-and amino-groups strongly hinder the free rotation, the presence of one major relative configuration can be safely assumed. The diastereotopic H-atoms show a geminal coupling constant of J = 14.3 Hz, whereas the vicinal coupling constants depend on the relative configurations of the molecule. In *anti*-**6a**, the diastereotopic protons each have a vicinal proton coupling partner in *cis*- and *trans*- configurations. The *trans* vicinal coupling yields a J value of 8.6 Hz, while the cis coupling constants are 3.1 and 3.5 Hz. In *syn*-**6a** the chemical shifts of the two diastereotopic protons H-3 are almost identical. Even at 700 MHz they are only barely separated yielding a strongly coupled signal, which does not allow a straightforward extraction of coupling constants. However, the signals can be simulated with the proper coupling constants. Figure S19 shows the experimental multiplet of the H-3 protons in a) and the calculated one in b). For the calculated spectrum the following coupling constants and chemical shifts were used: δ (^{1}H) H-3 pro*R*: 1.515 ppm δ (^{1}H) H-3 pro*S*: 1.525 ppm, ^{2}J (H3pro*R*-H3pro*S*): 14.2 Hz, ^{3}J (H2-H3pro*R*): 10.0 Hz, ^{3}J (H2-H3pro*S*): 3.0 Hz, ^{3}J (H4-H3 pro*R*): 6.6 Hz, ^{3}J (H4-H3pro*S*): 3.0 Hz. The strong coupling of the signals of the H-3 protons is also confirmed by a pure shift spectrum,[3] (Figure S19c) which yields only one broad signal.

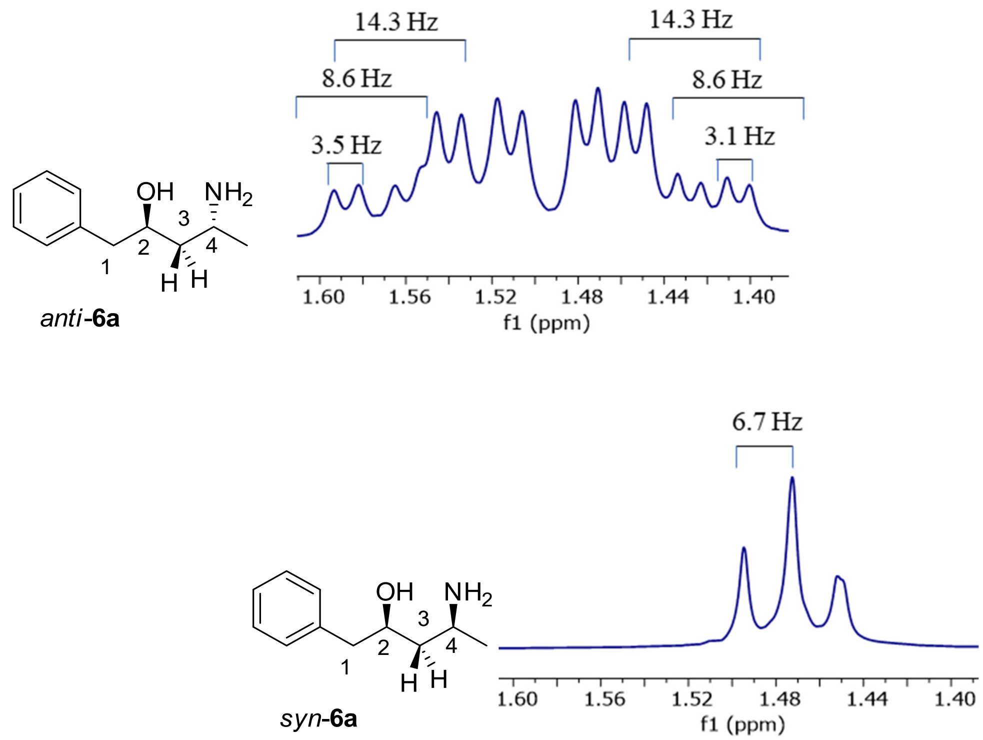

Figure S18. 1H-NMR spectra of the diastereotopic C3 H-atoms and scalar coupling constants of product *anti*-**6a** and *syn*-**6a**.

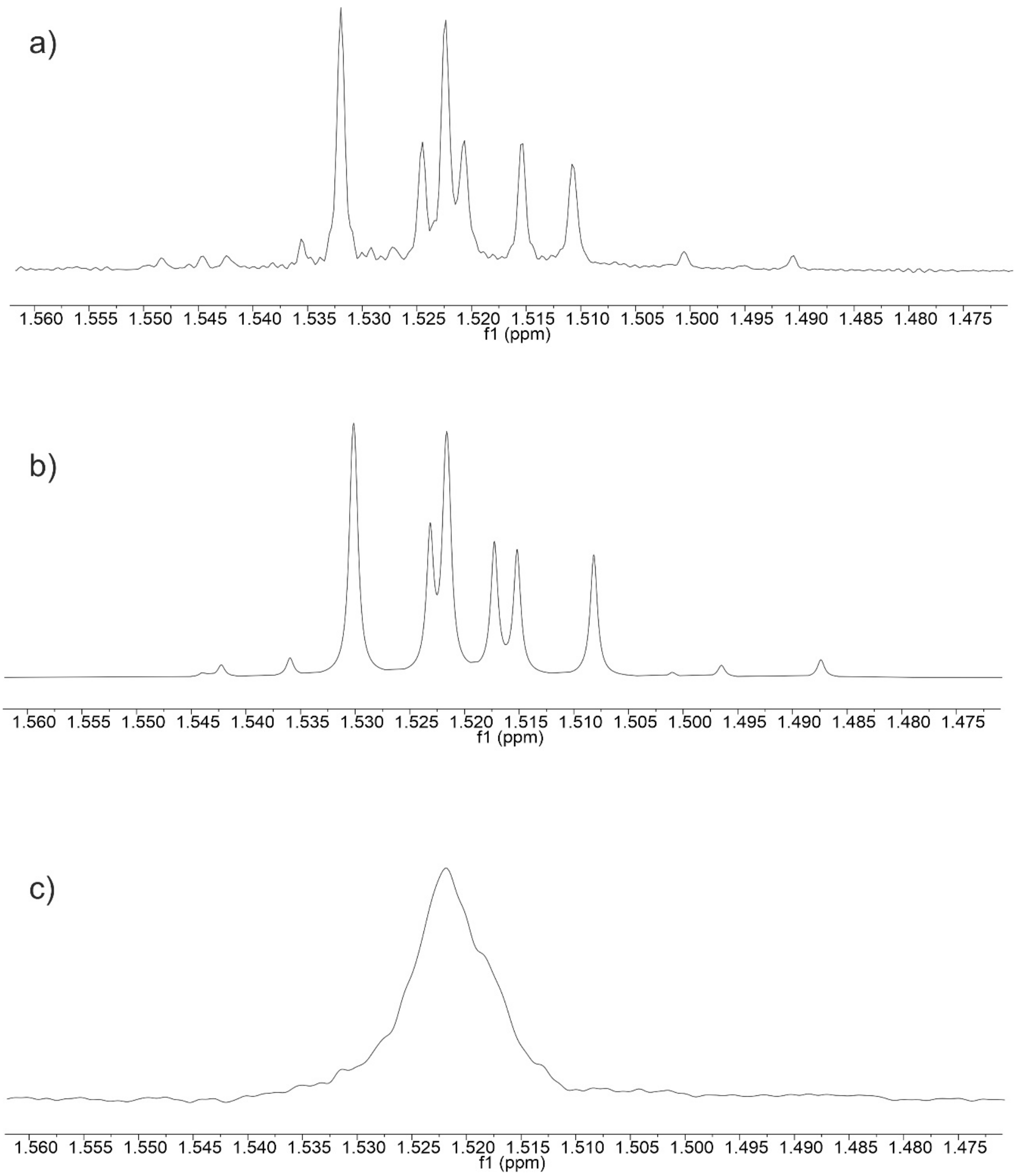

Figure S19. a) Experimental multiplet of the H-3 proton shifts in *syn*-**6a** at 700 MHz. b) Calculated multiplet of the H-3 protons shifts in *syn*-**6a**. c) Pure shift spectrum of H-3 proton shifts in *syn*-**6a**.

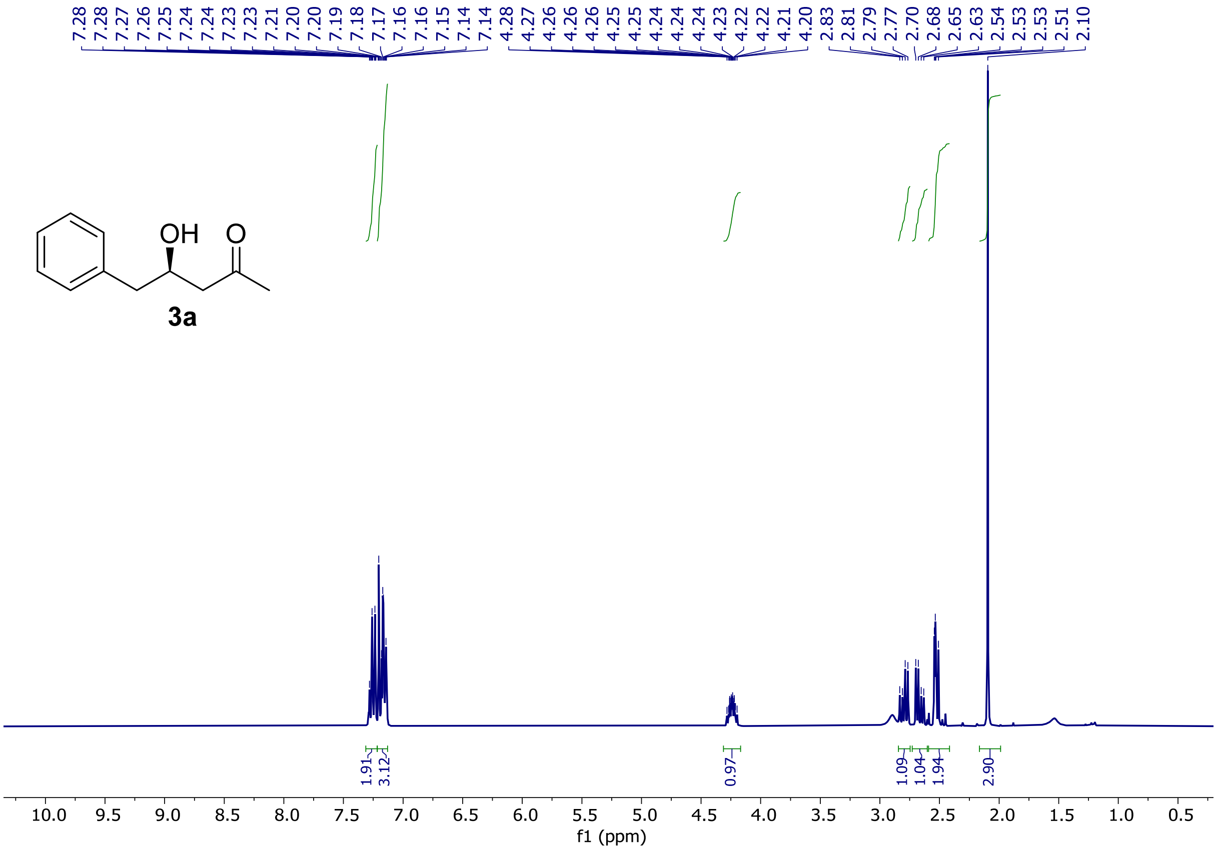

Figure S20. ^{1}H-NMR spectrum in d-$CHCl_3$ of aldol product **3a**.

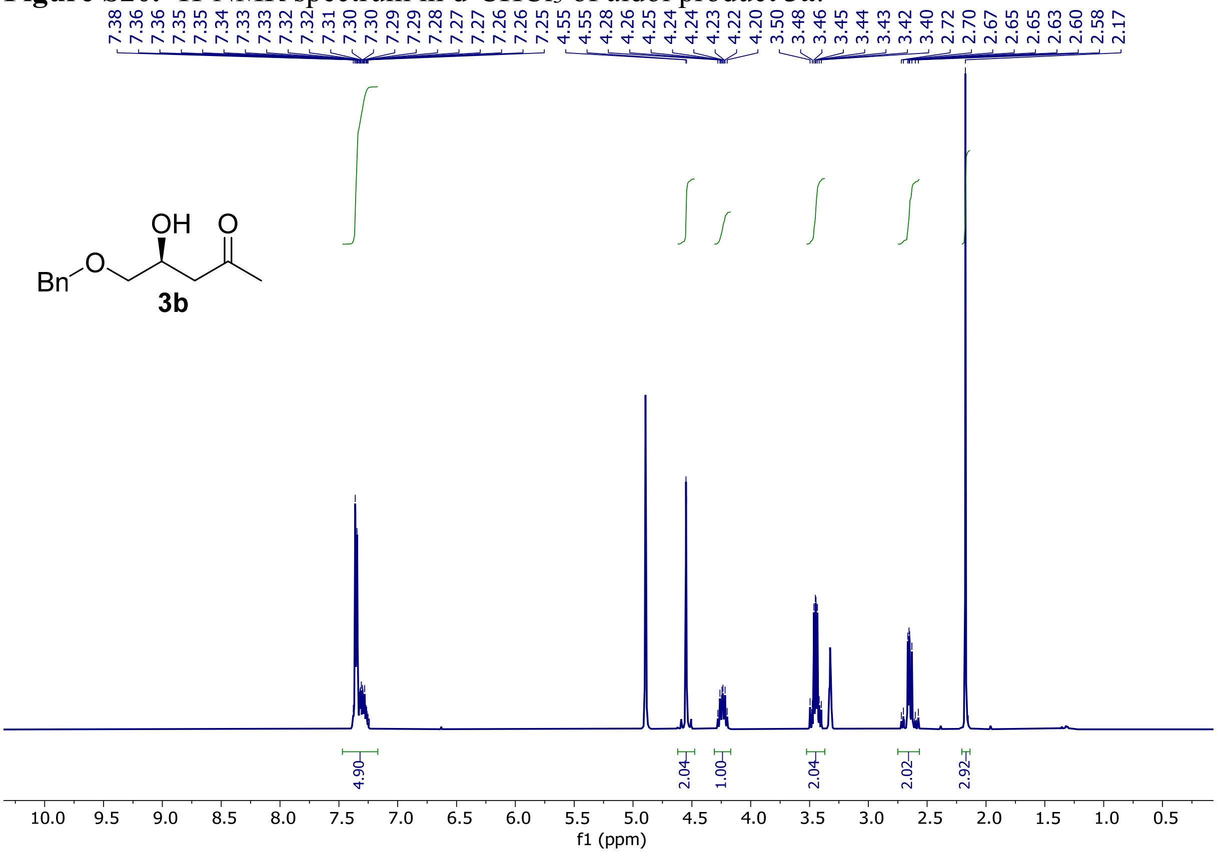

Figure S21. ^{1}H-NMR spectrum in d_4-MeOH of aldol product **3b**.

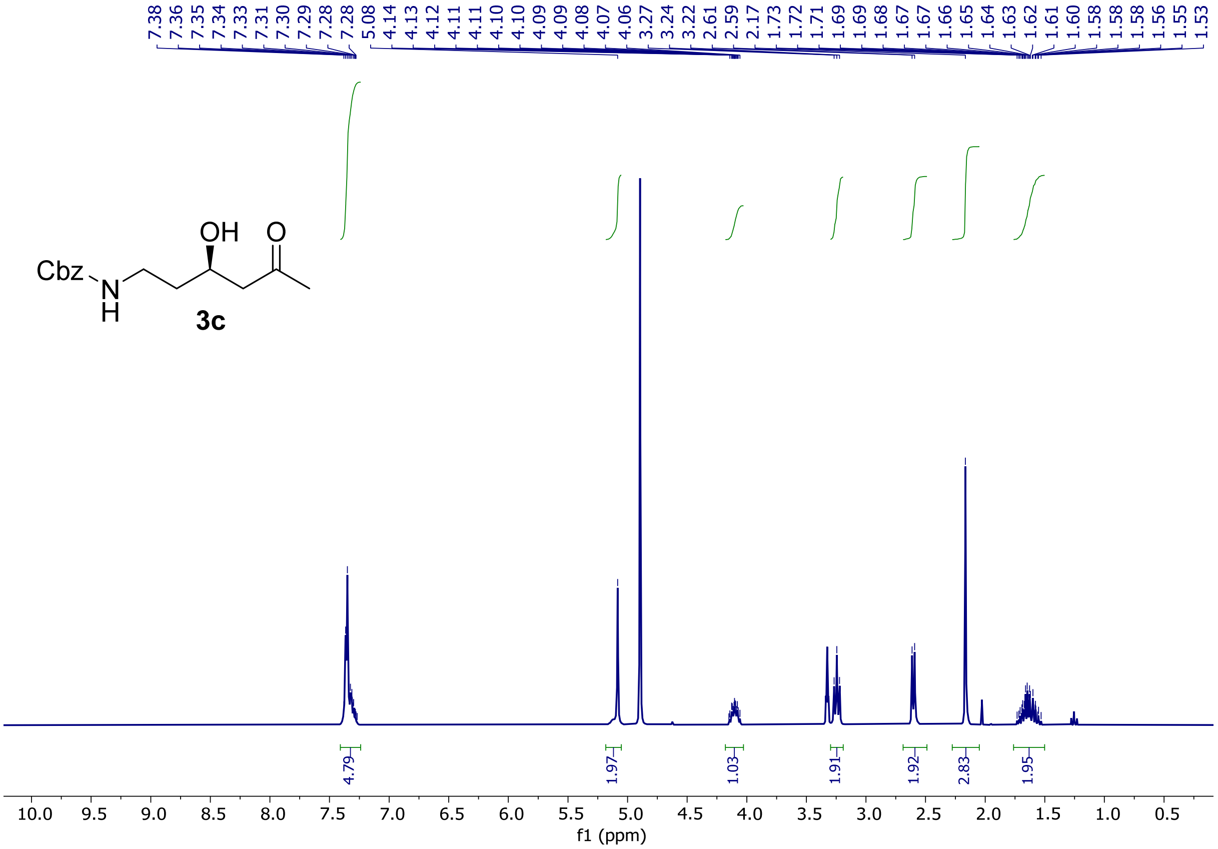

Figure S22. ^{1}H-NMR spectrum in d$_4$-MeOH of aldol product **3c**.

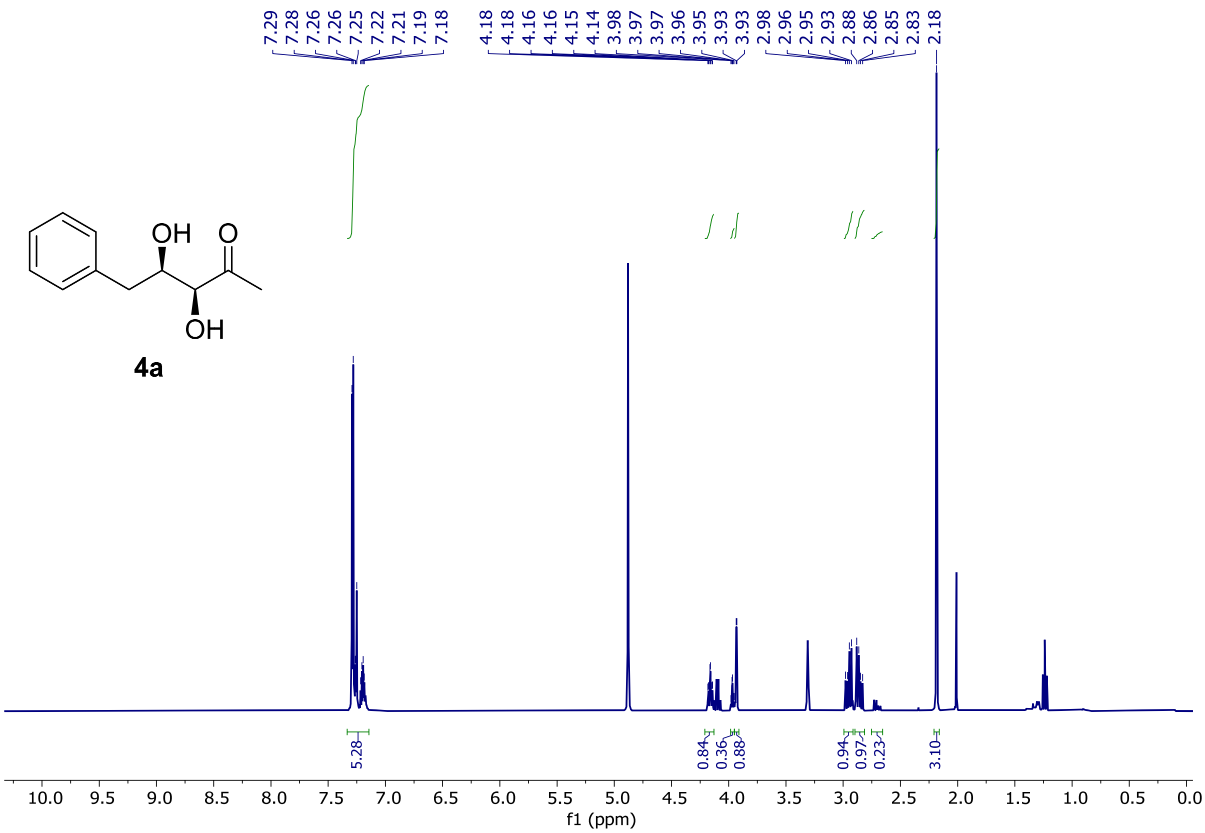

Figure S23. ^{1}H-NMR spectrum in d_4-MeOH of aldol product **4a**.

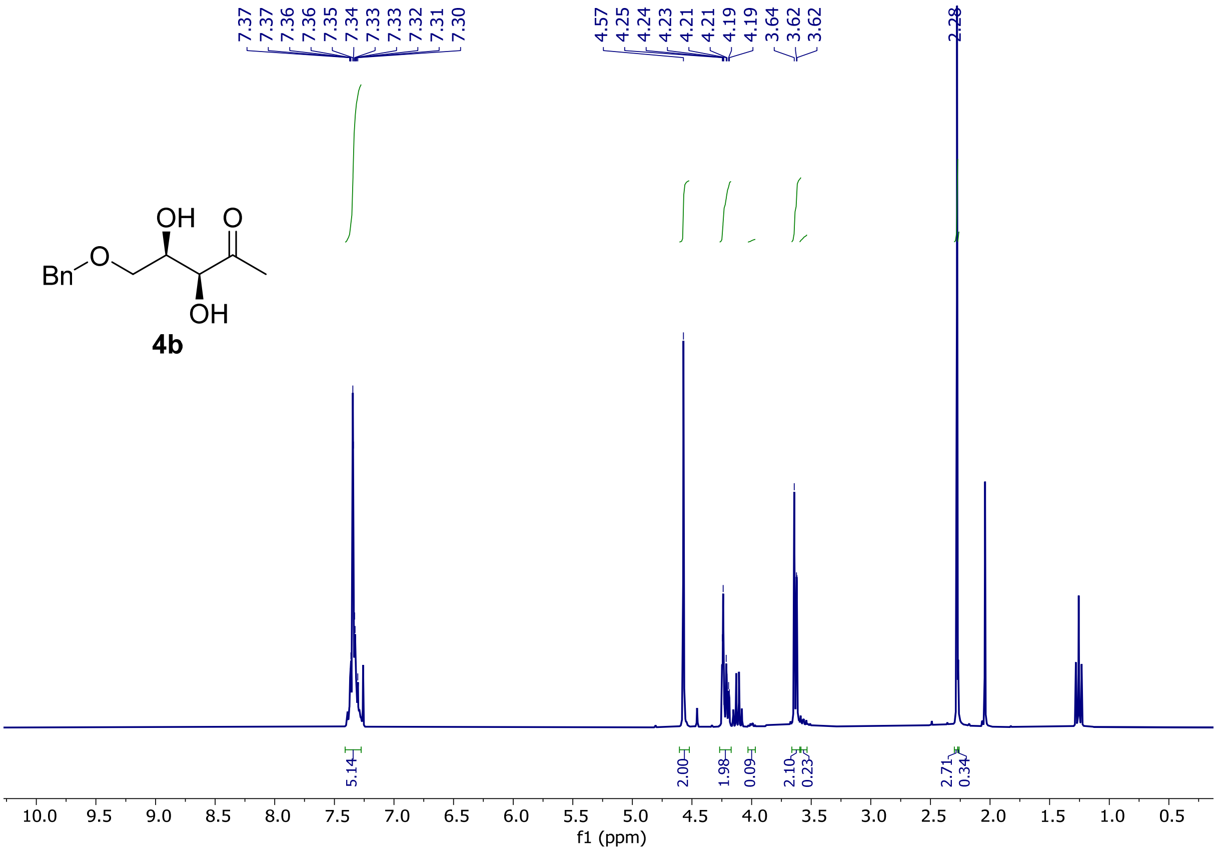

Figure S24. ^{1}H-NMR spectrum in d-$CHCl_3$ of aldol product **4b**.

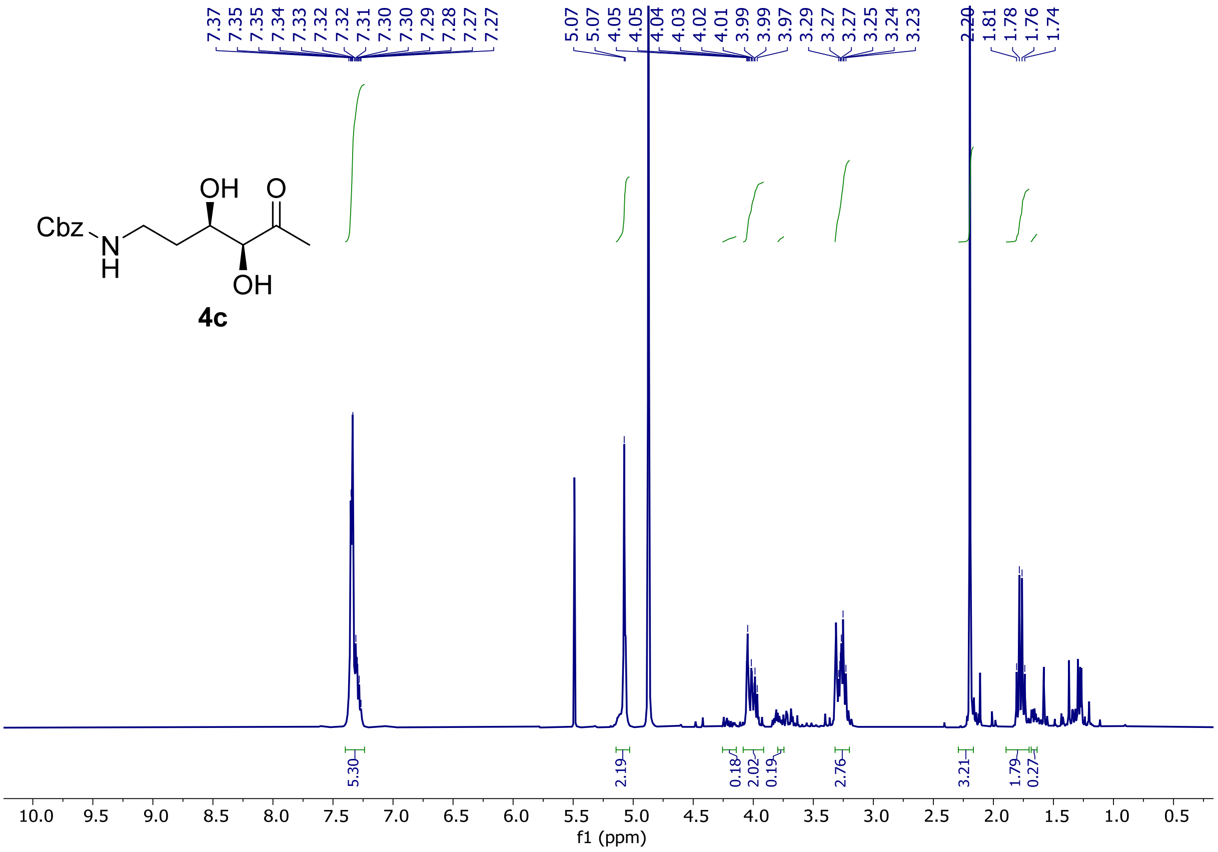

Figure S25. ^{1}H-NMR spectrum d4-MeOH of aldol product **4c**.

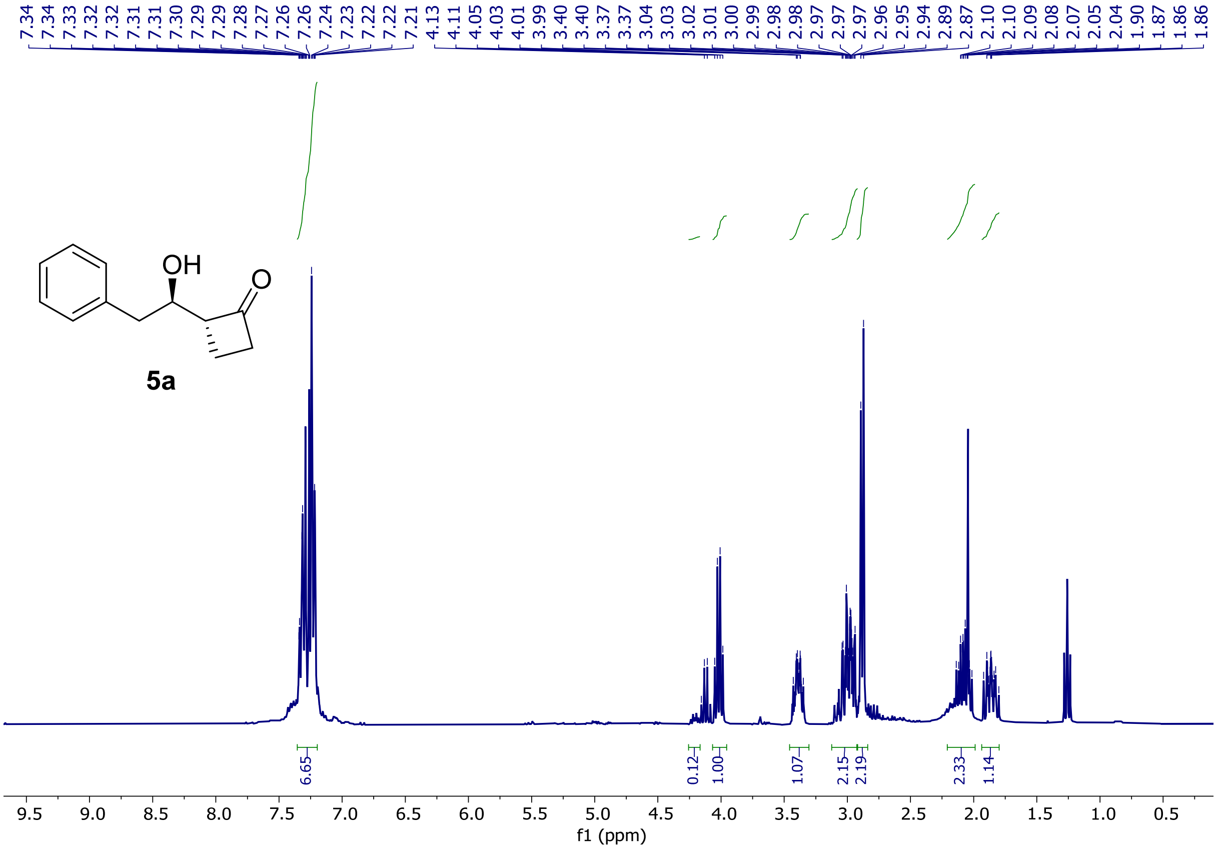

Figure S26. ^{1}H-NMR spectrum in d-CHCl3of aldol product **5a**.

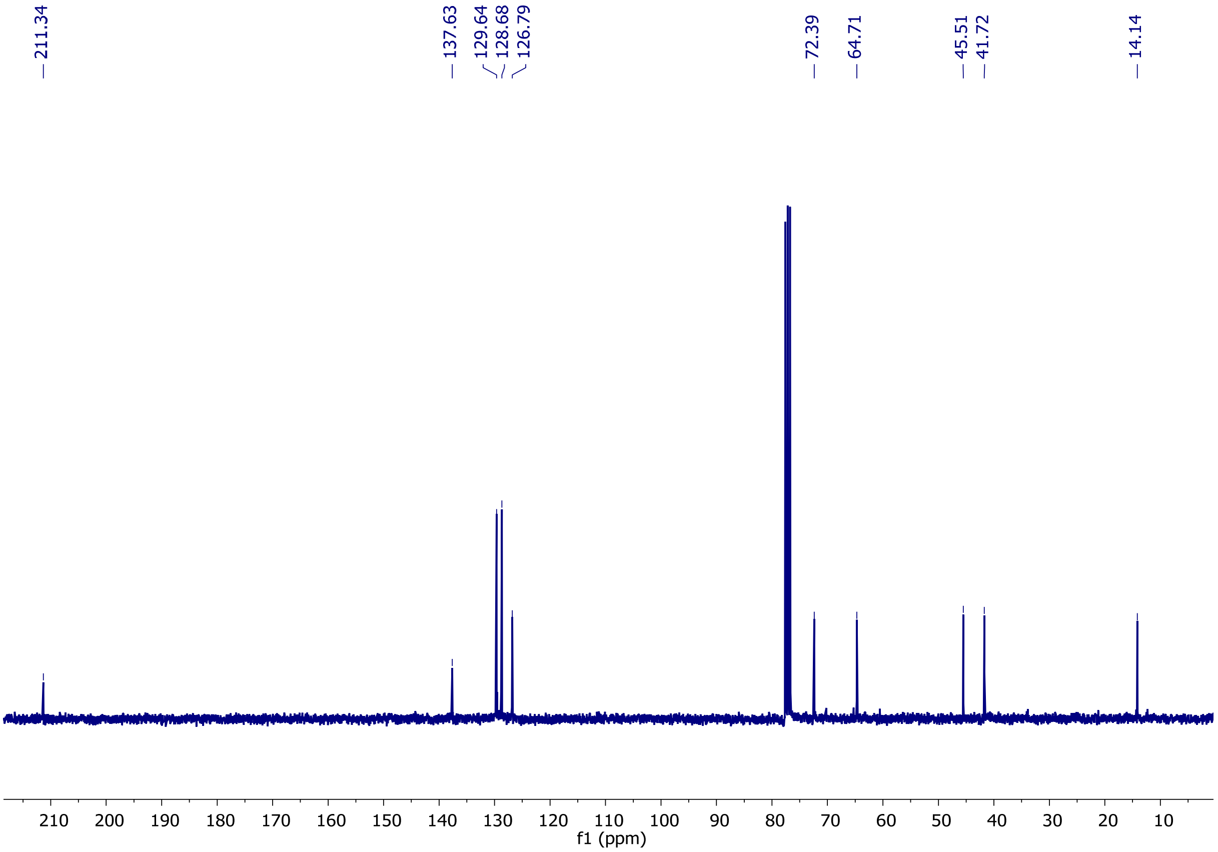

Figure S27. ^{13}C-NMR spectrum in d-$CHCl_3$ of aldol product **5a**.

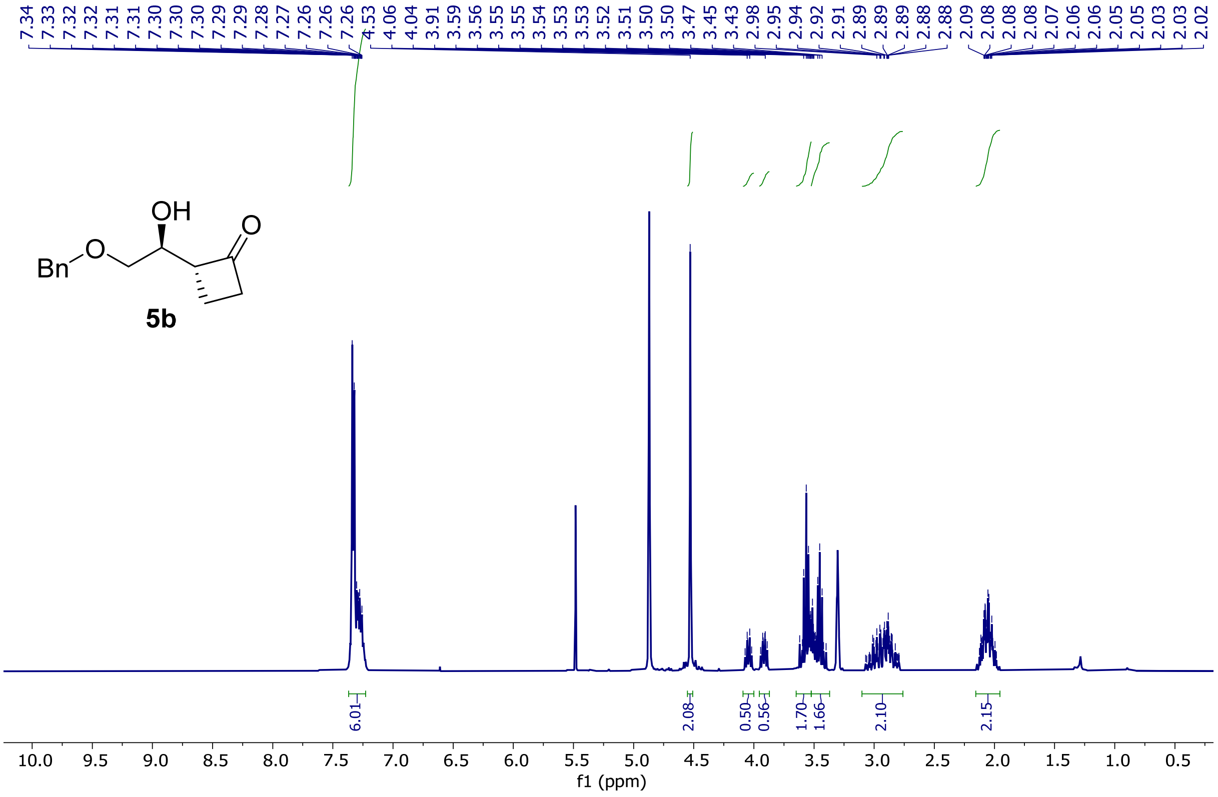

Figure S28. ^{1}H-NMR spectrum in d_4-MeOH of aldol product **5b**.

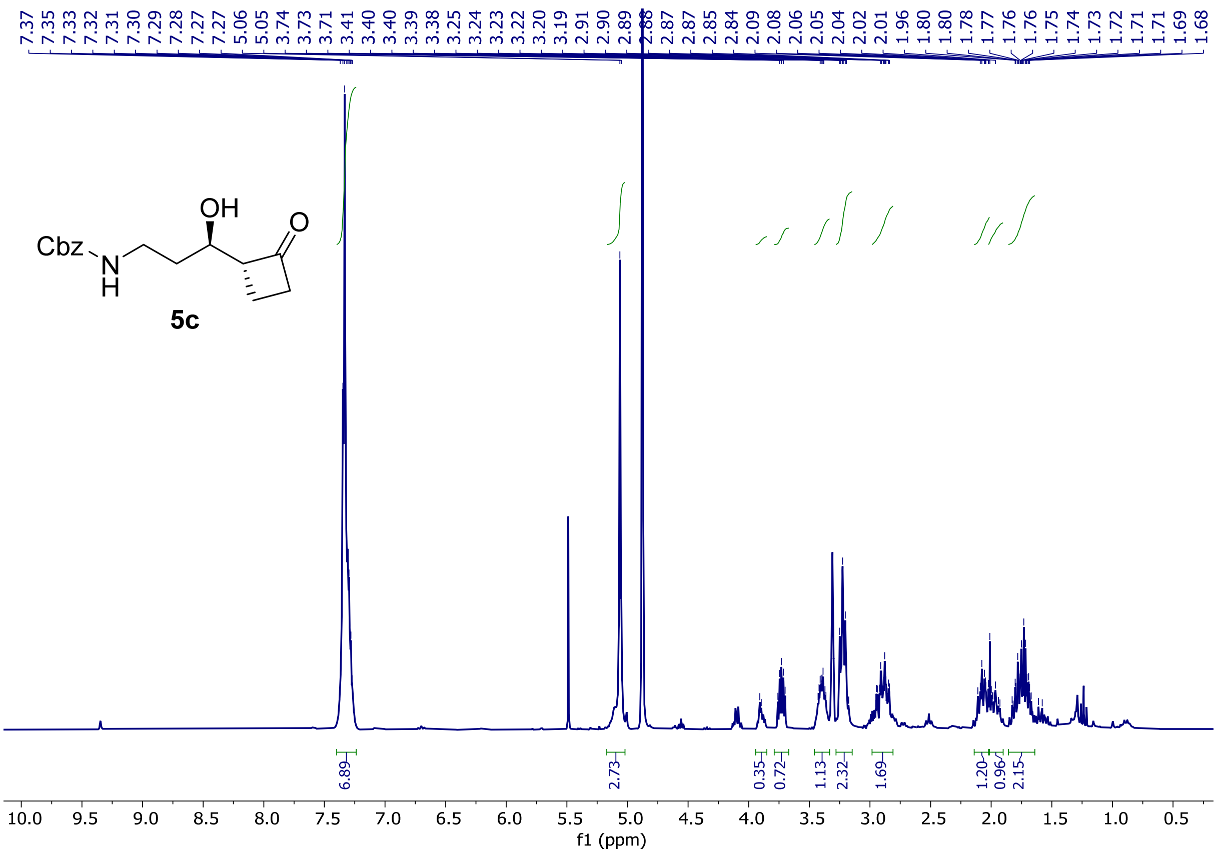

Figure S29. ^{1}H-NMR spectrum in d$_4$-MeOH of aldol product **5c**.

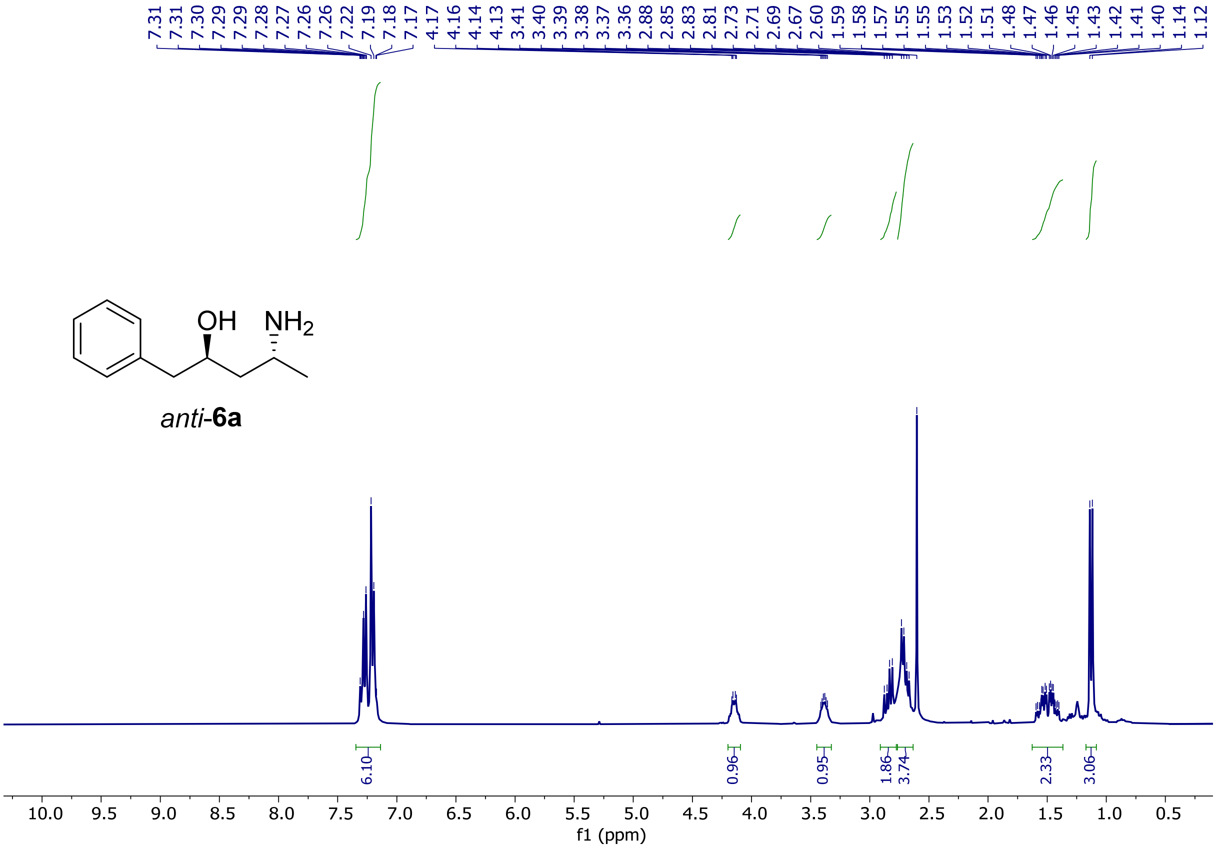

Figure S30. ^{1}H-NMR spectrum in d-$CHCl_3$ of product *anti*-**6a**.

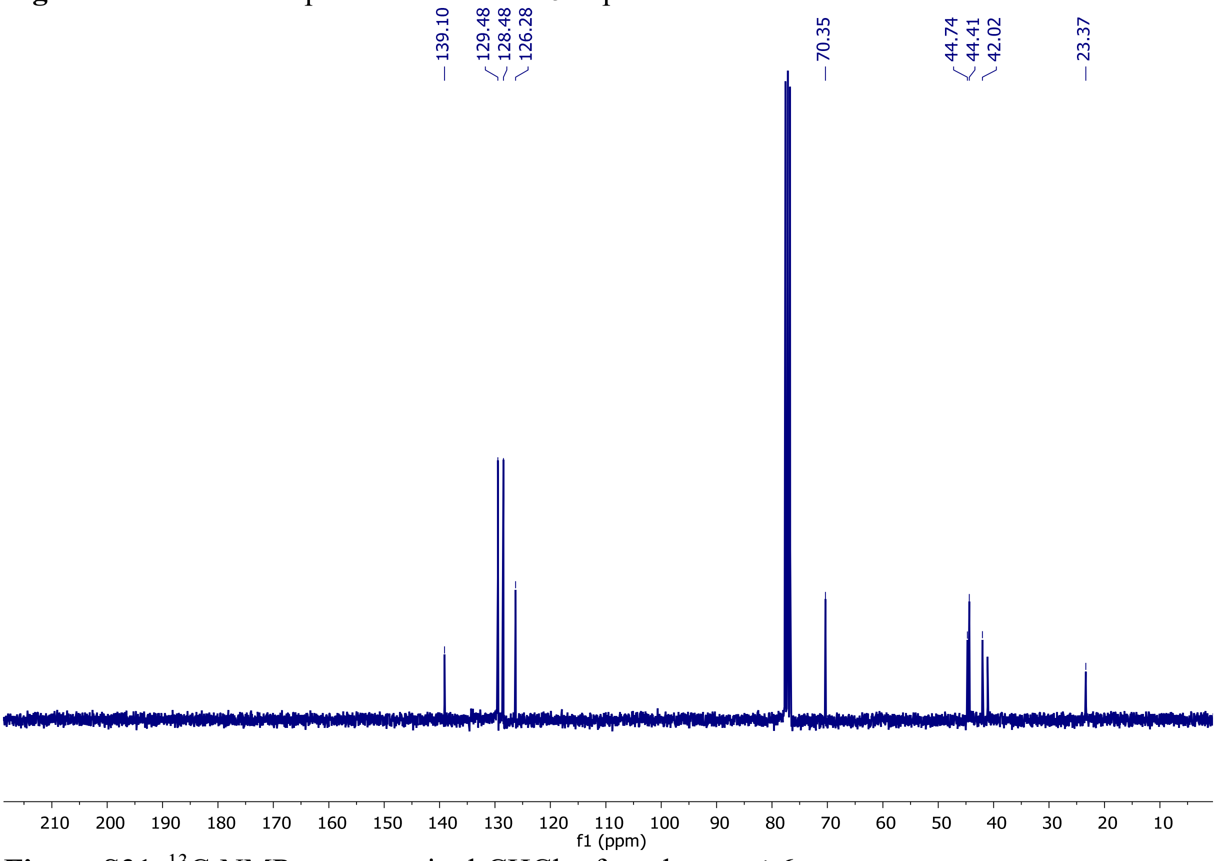

Figure S31. ^{13}C-NMR spectrum in d-$CHCl_3$ of product *anti*-**6a**.

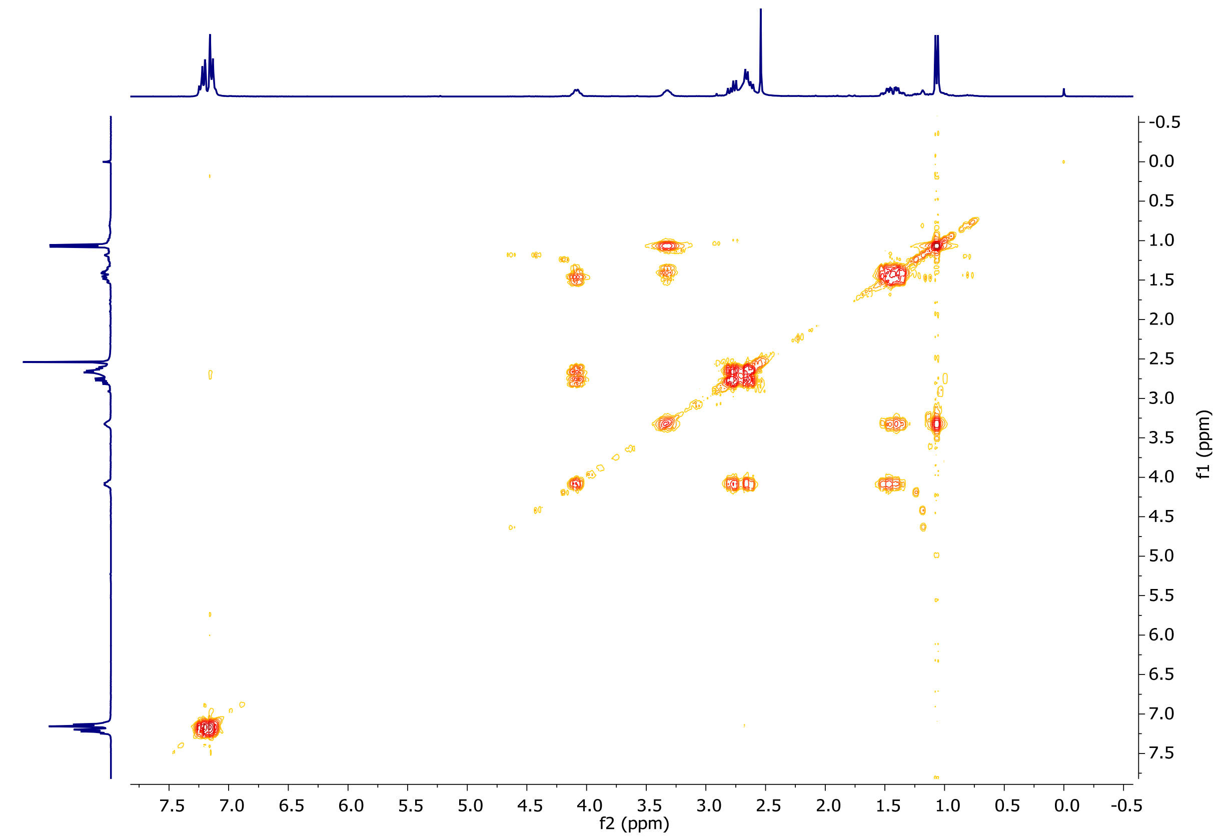

Figure S32. COSY spectrum in d-CHCl3 of product *anti*-**6a**.

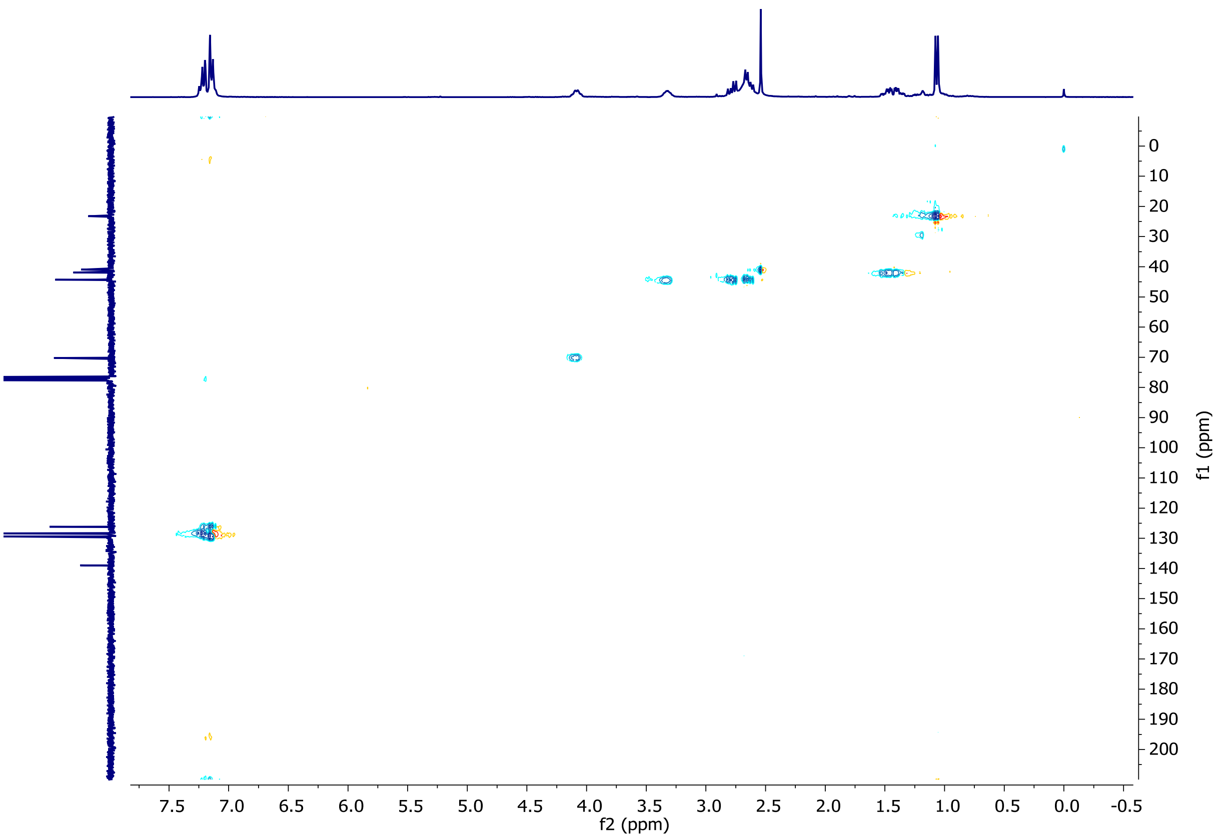

Figure S33. HSQC spectrum in in d-CHCl3 of product *anti*-**6a**.

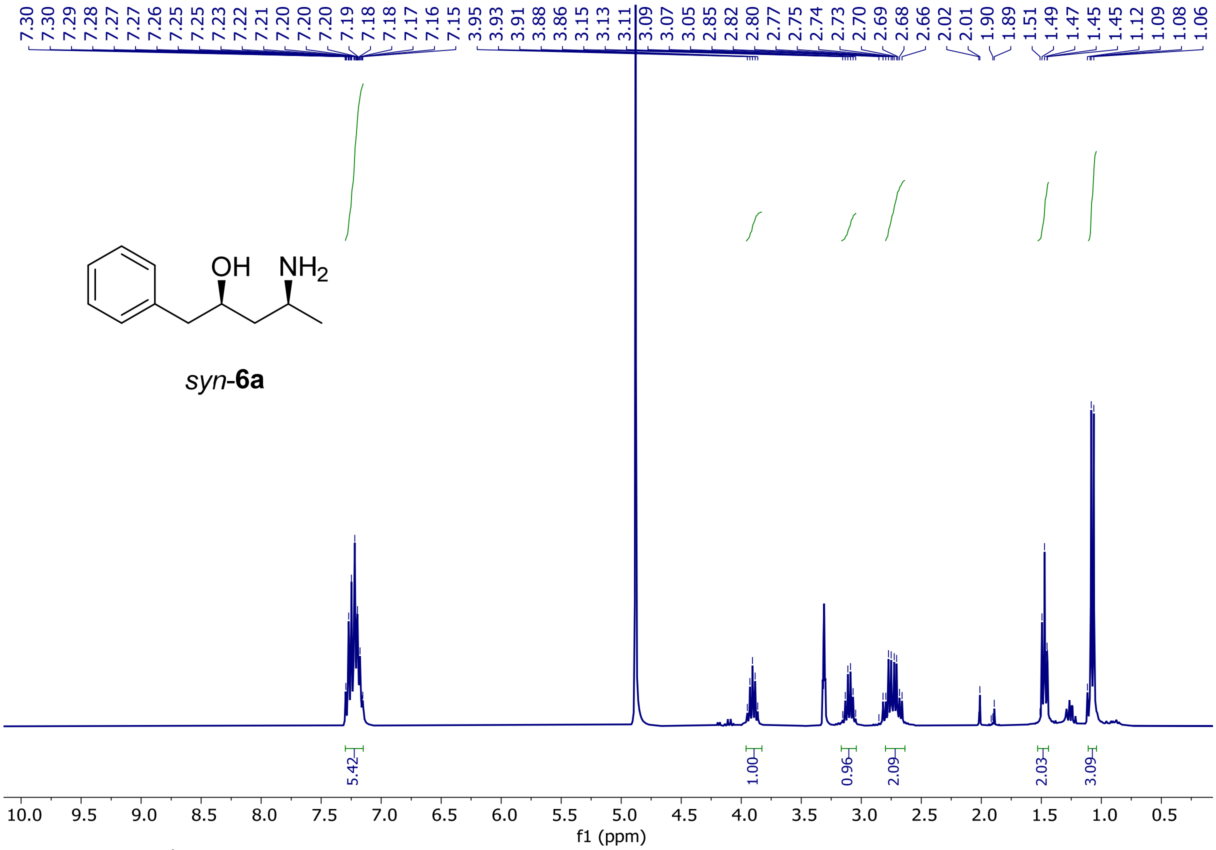

Figure S34. ^{1}H-NMR spectrum in d_4-MeOH of product *syn*-**6a**.

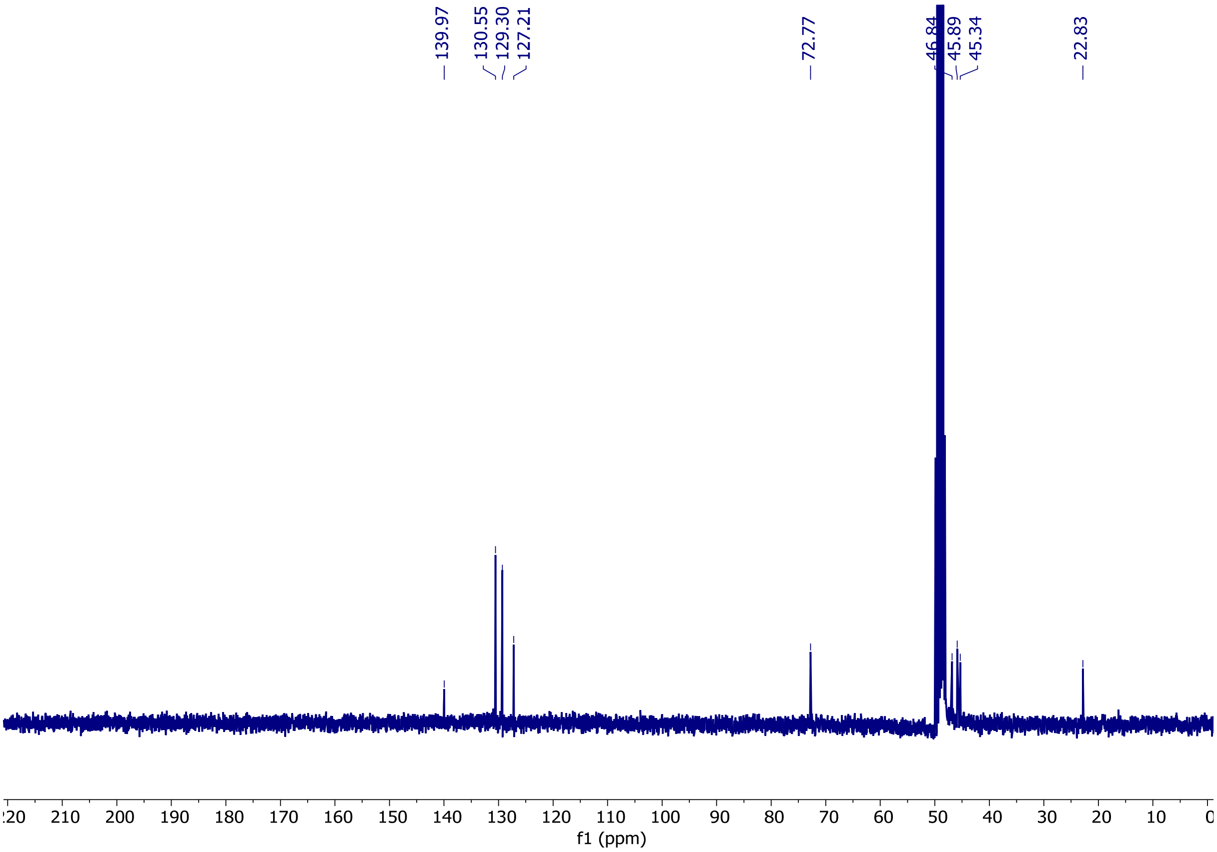

Figure S35. ^{13}C-NMR spectrum in d_4-MeOH of product *syn*-**6a**.

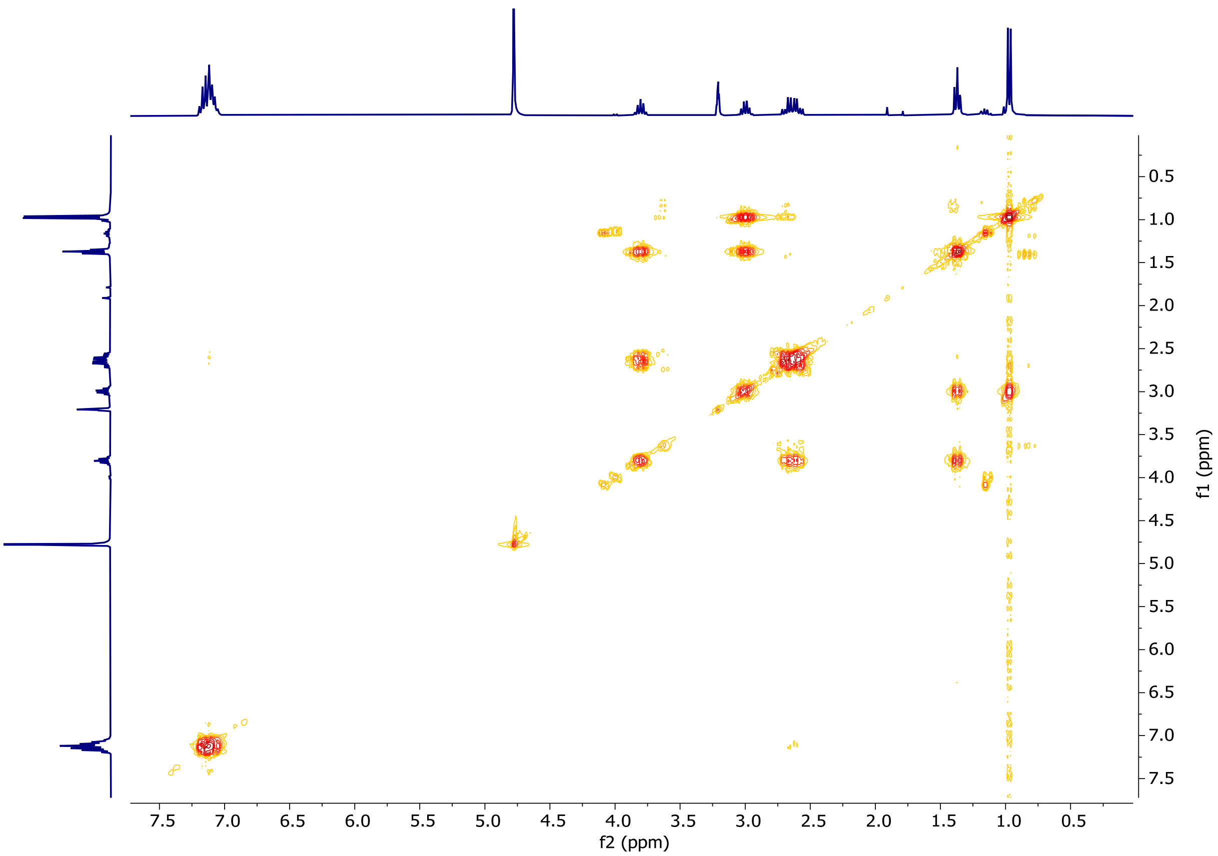

Figure S36. COSY spectrum in d$_4$-MeOH of product *syn*-**6a**.

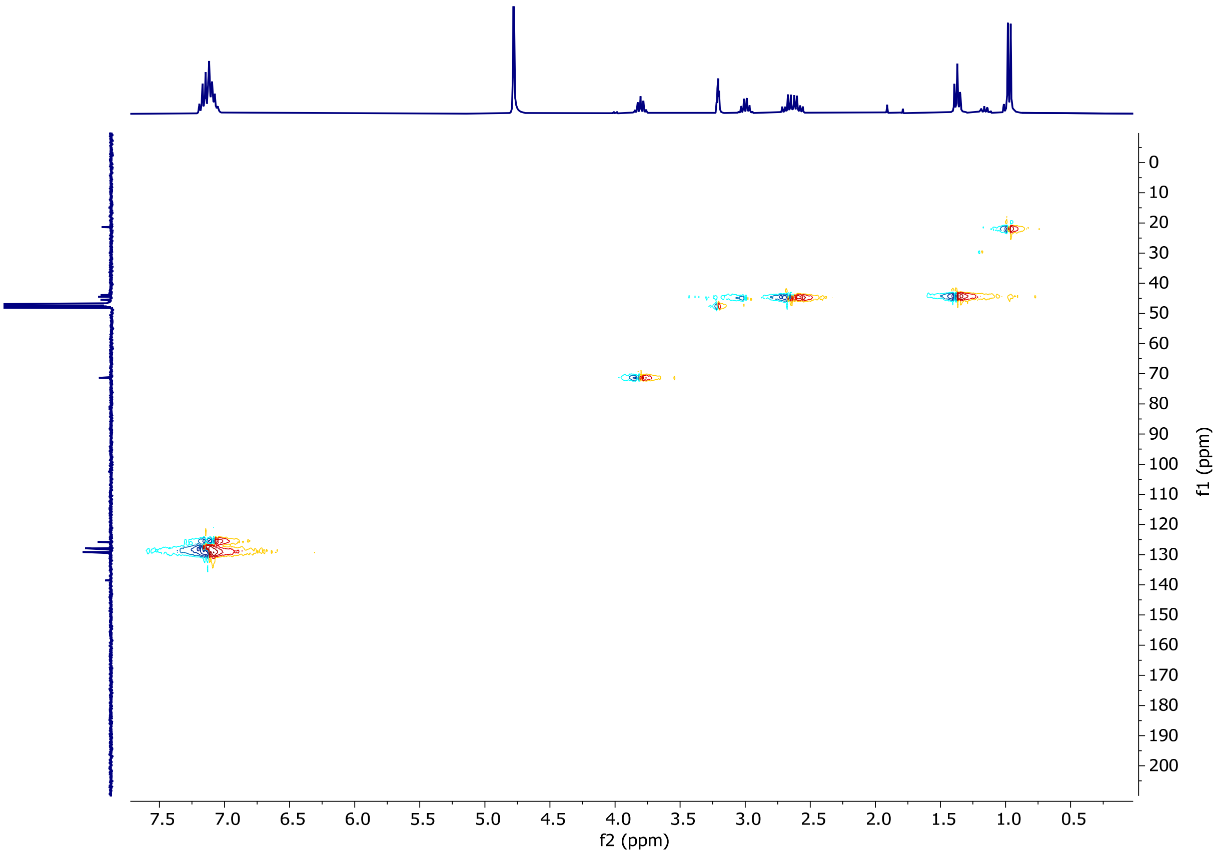

Figure S37. HSQC NMR spectrum in d$_4$-MeOH of product *syn*-**6a**.

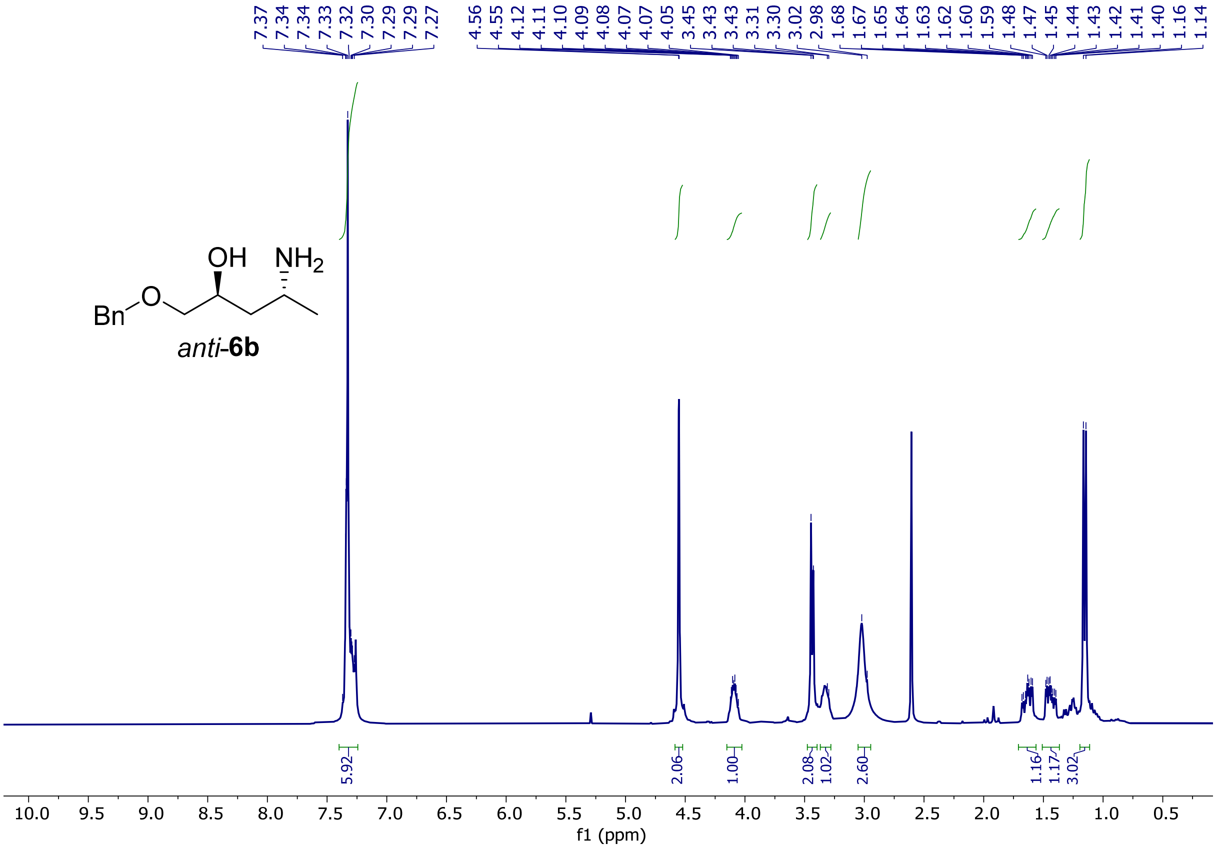

Figure S38. ^{1}H-NMR spectrum in d-$CHCl_3$ of product *anti*-**6b**.

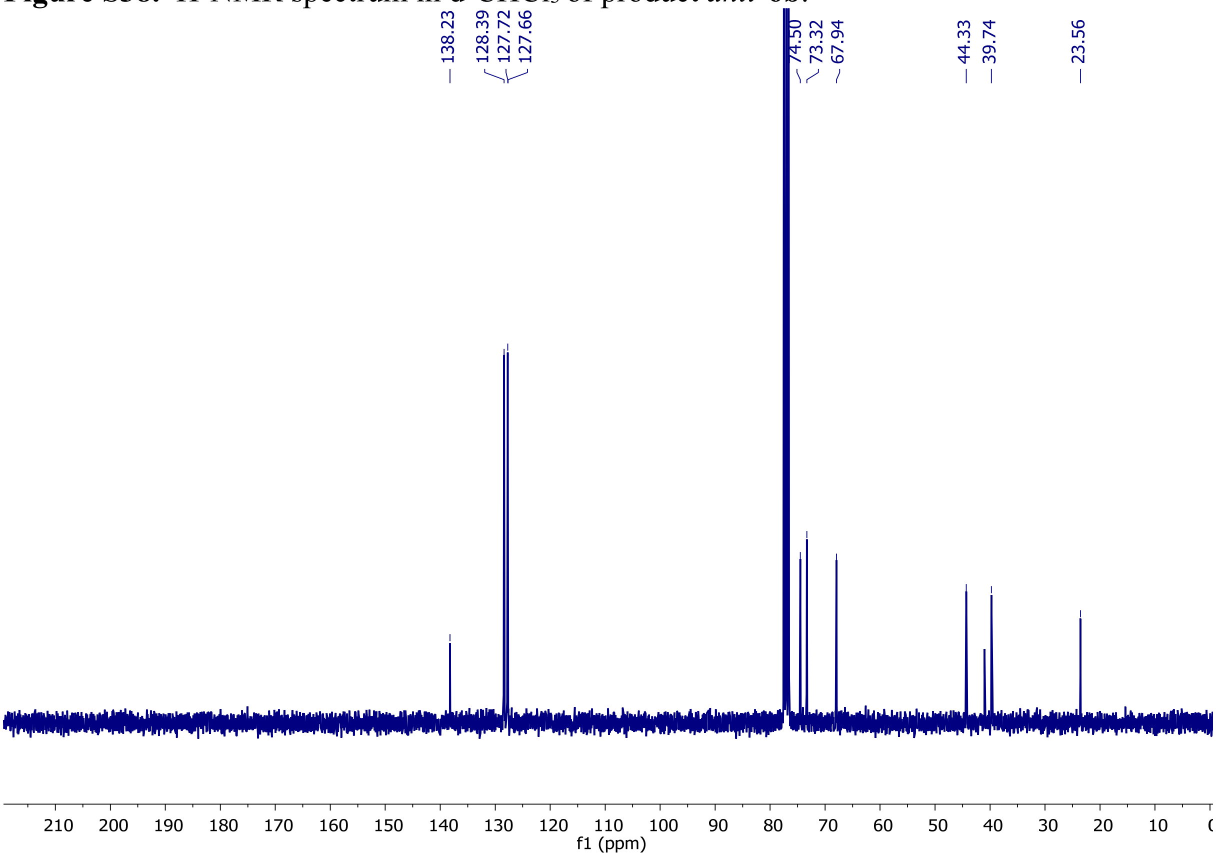

Figure S39. ^{13}C-NMR spectrum in d-$CHCl_3$ of product *anti*-**6b**.

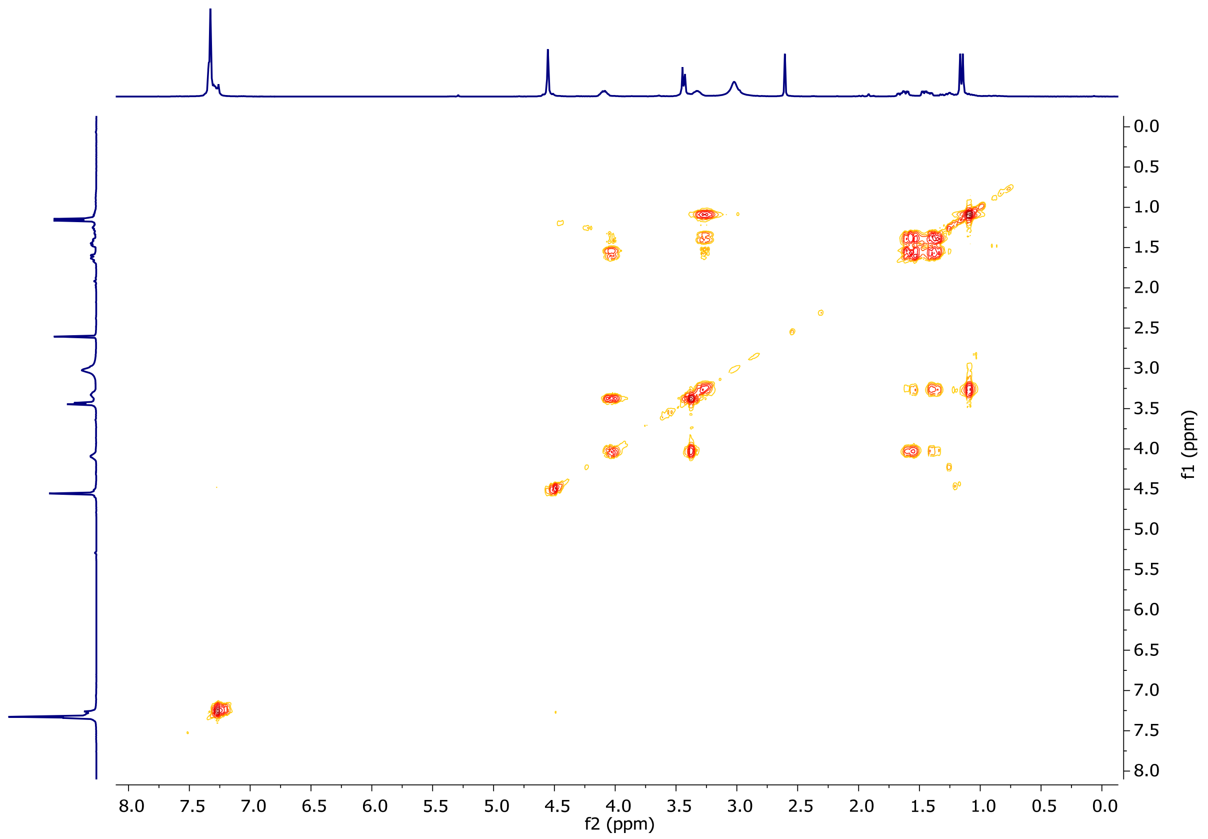

Figure S40. COSY spectrum in d-CHCl3 of product *anti*-**6b**.

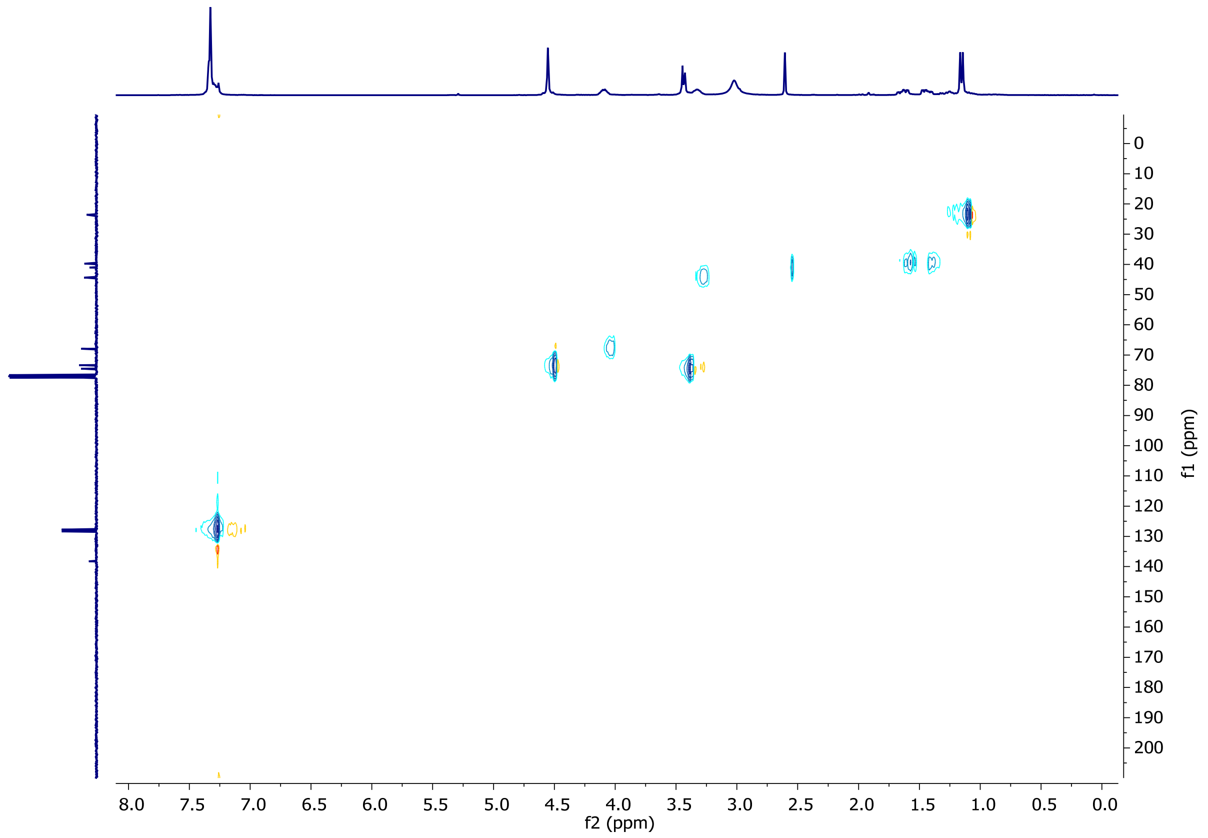

Figure S41. HSQC spectrum in d-CHCl3 of product *anti*-**6b**.

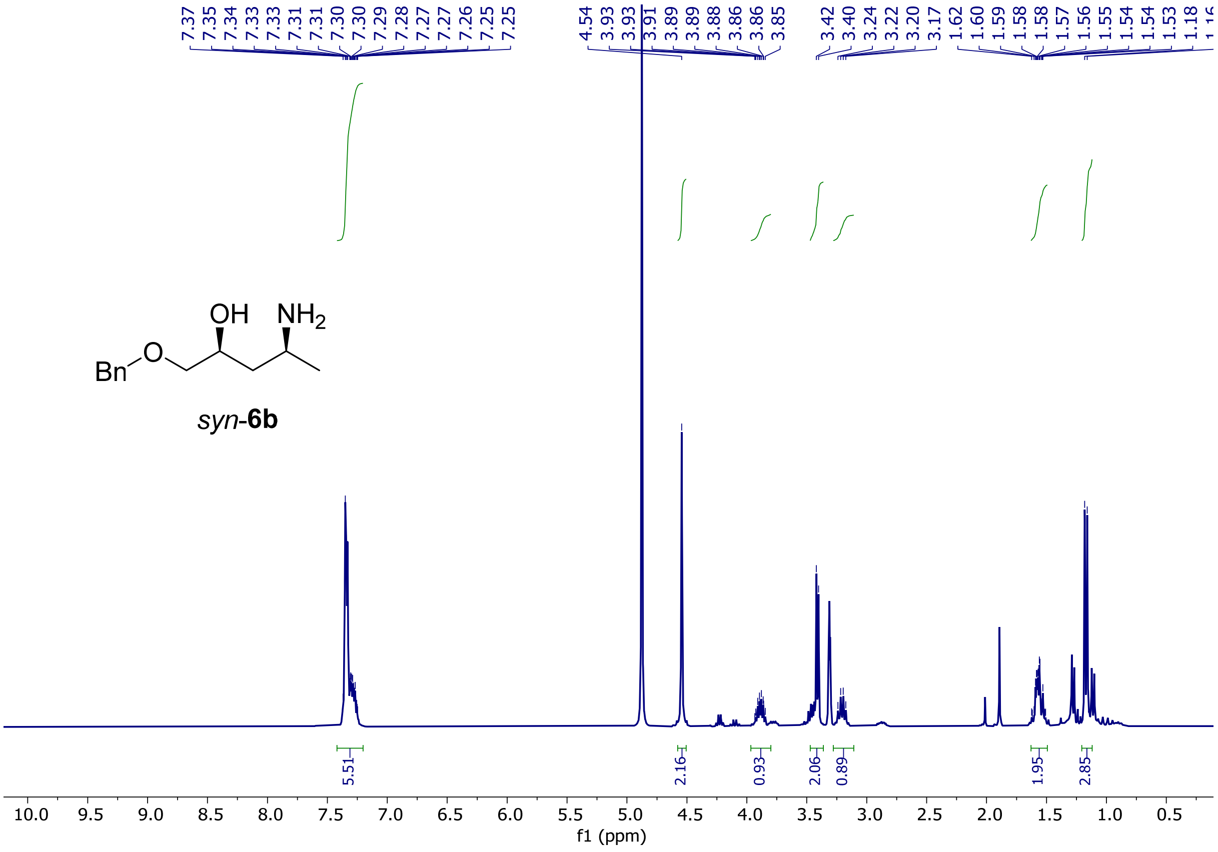

Figure S42. ^{1}H-NMR spectrum d_4-MeOH of product *syn*-**6b**.

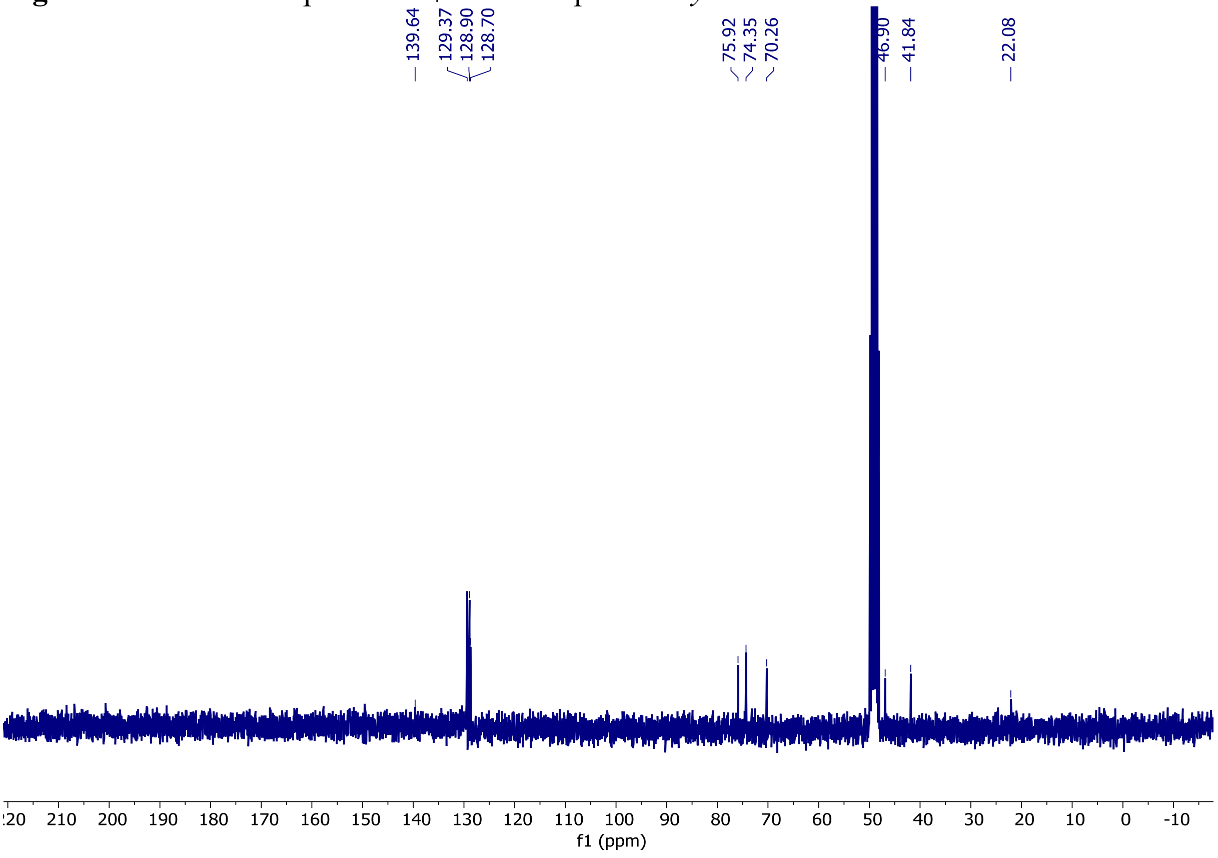

Figure S43. ^{13}C-NMR spectrum d_4-MeOH of product *syn*-**6b**.

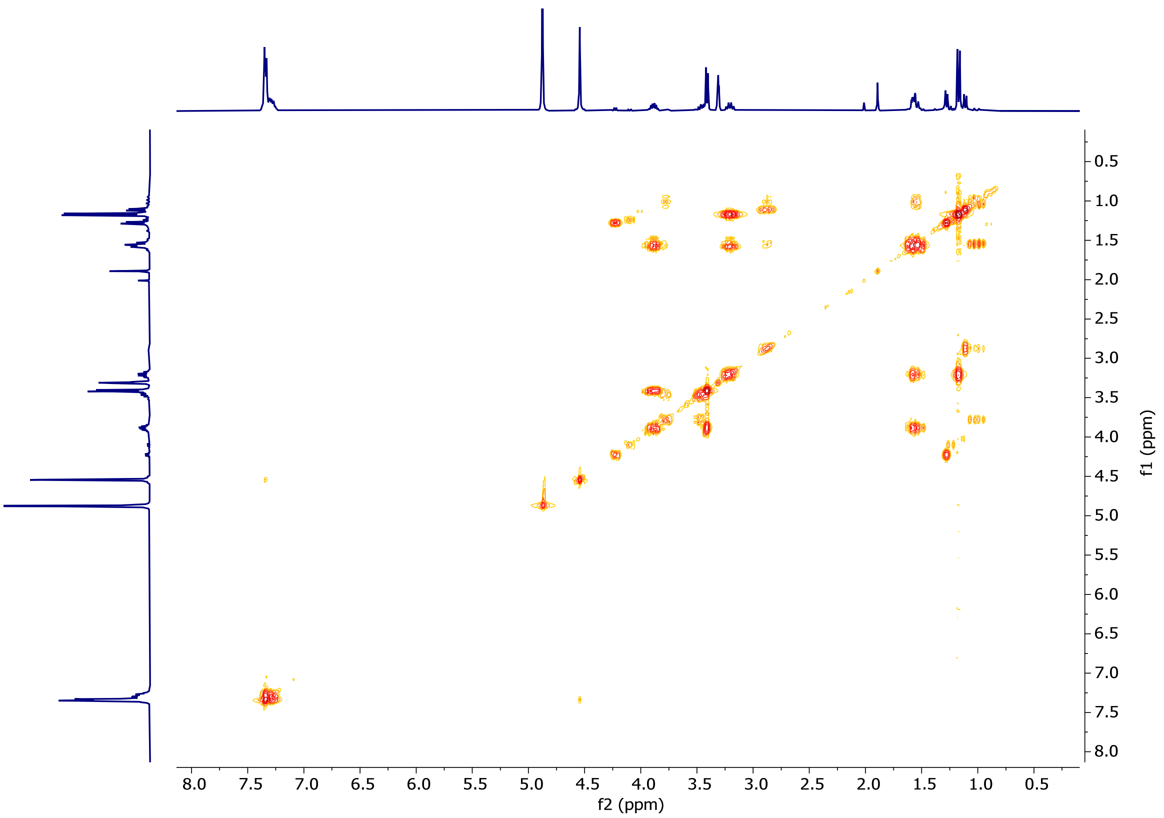

Figure S44. COSY NMR spectrum d$_4$-MeOH of product *syn*-**6b**.

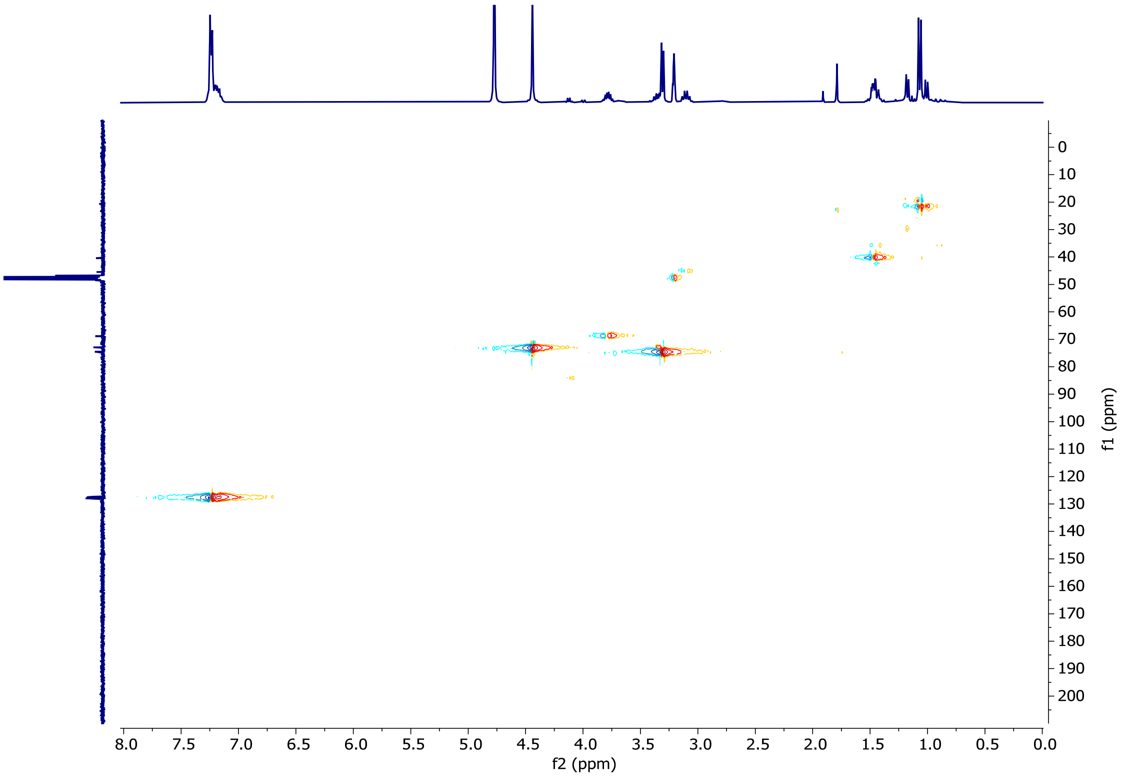

Figure S45. HSQC NMR spectrum d4-MeOH of product syn-**6b**.

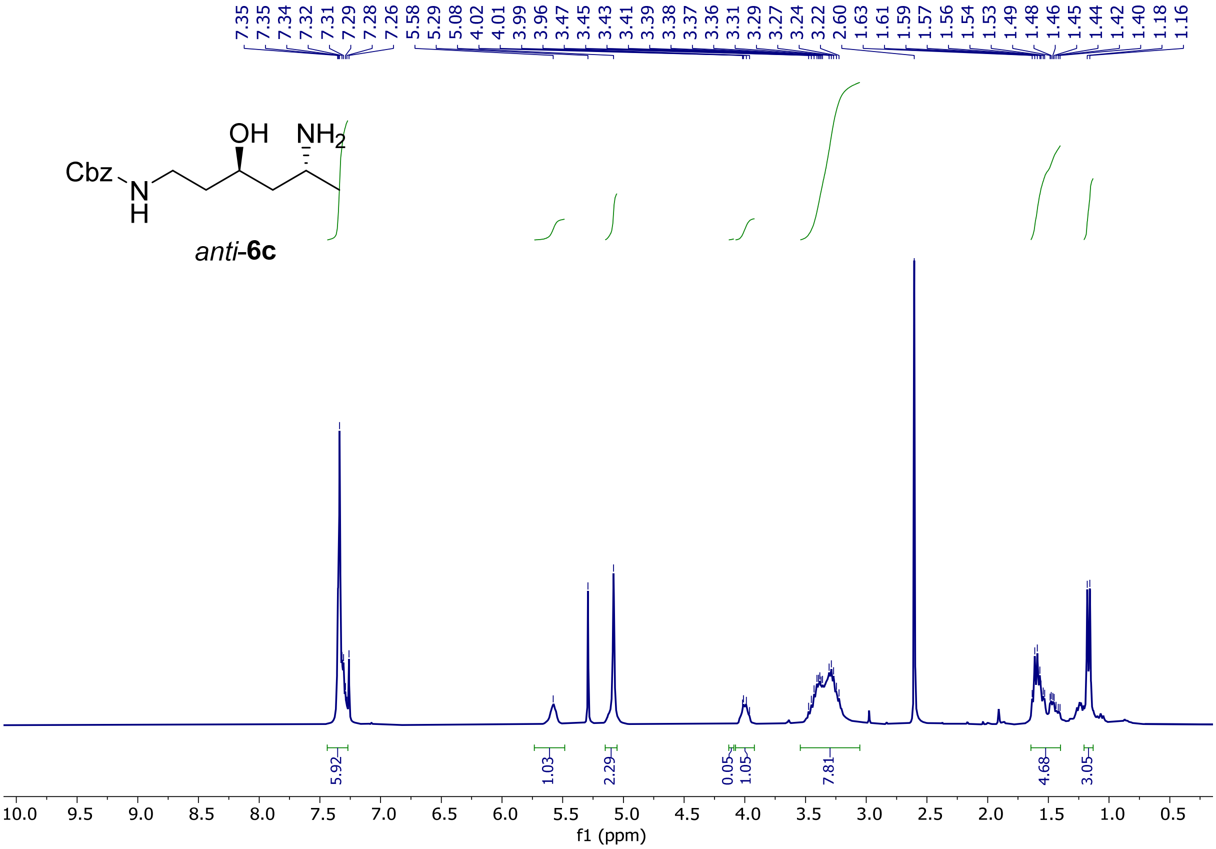

Figure S46. ^{1}H-NMR spectrum d-$CHCl_3$ of product *anti*-**6c**.

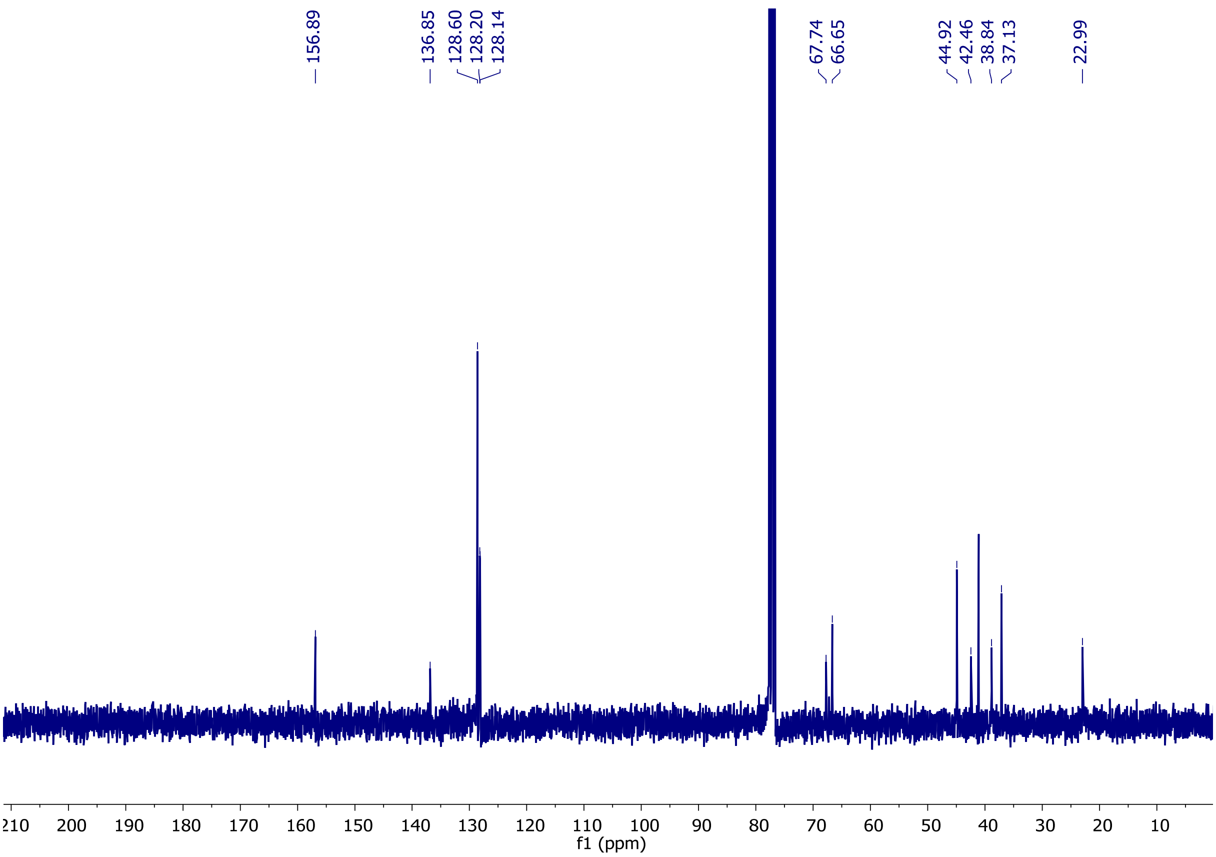

Figure S47. ^{13}C-NMR spectrum in d-$CHCl_3$ of product *anti*-**6c**.

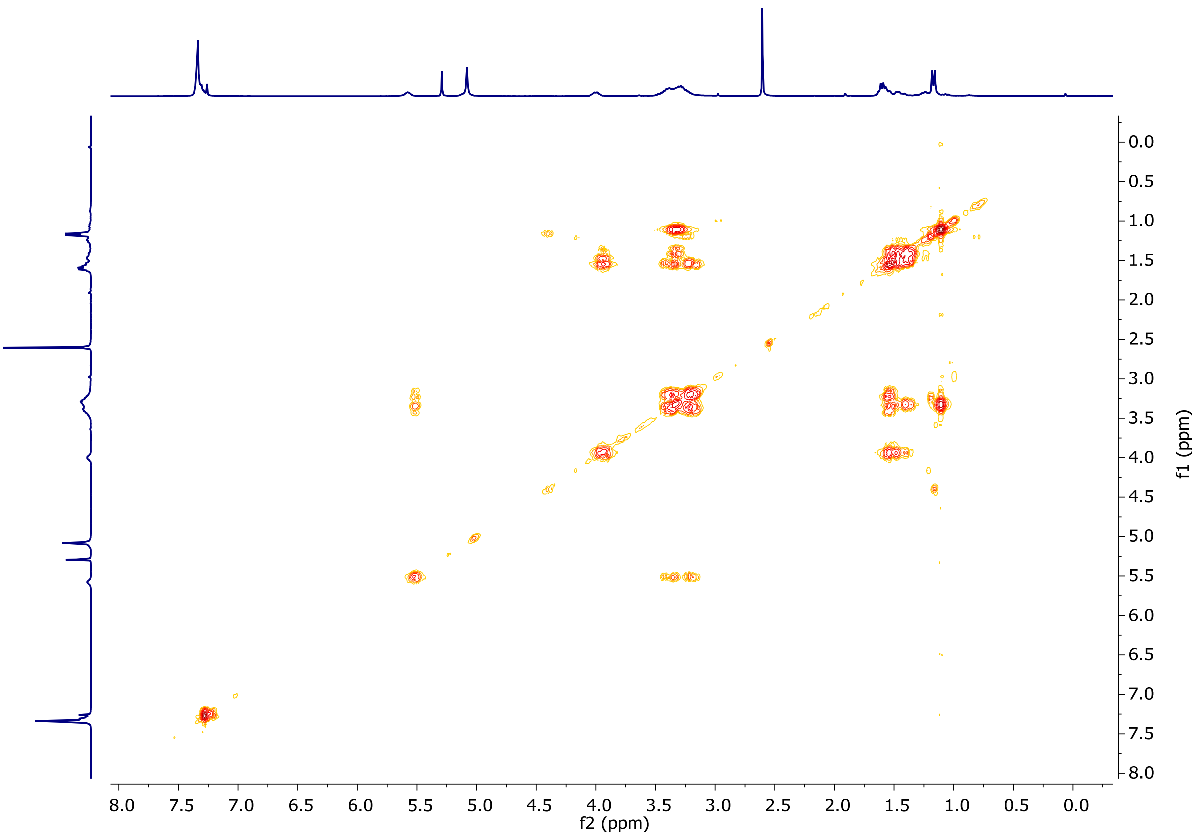

Figure S48. COSY spectrum d-$CHCl_3$ of product *anti*-**6c**.

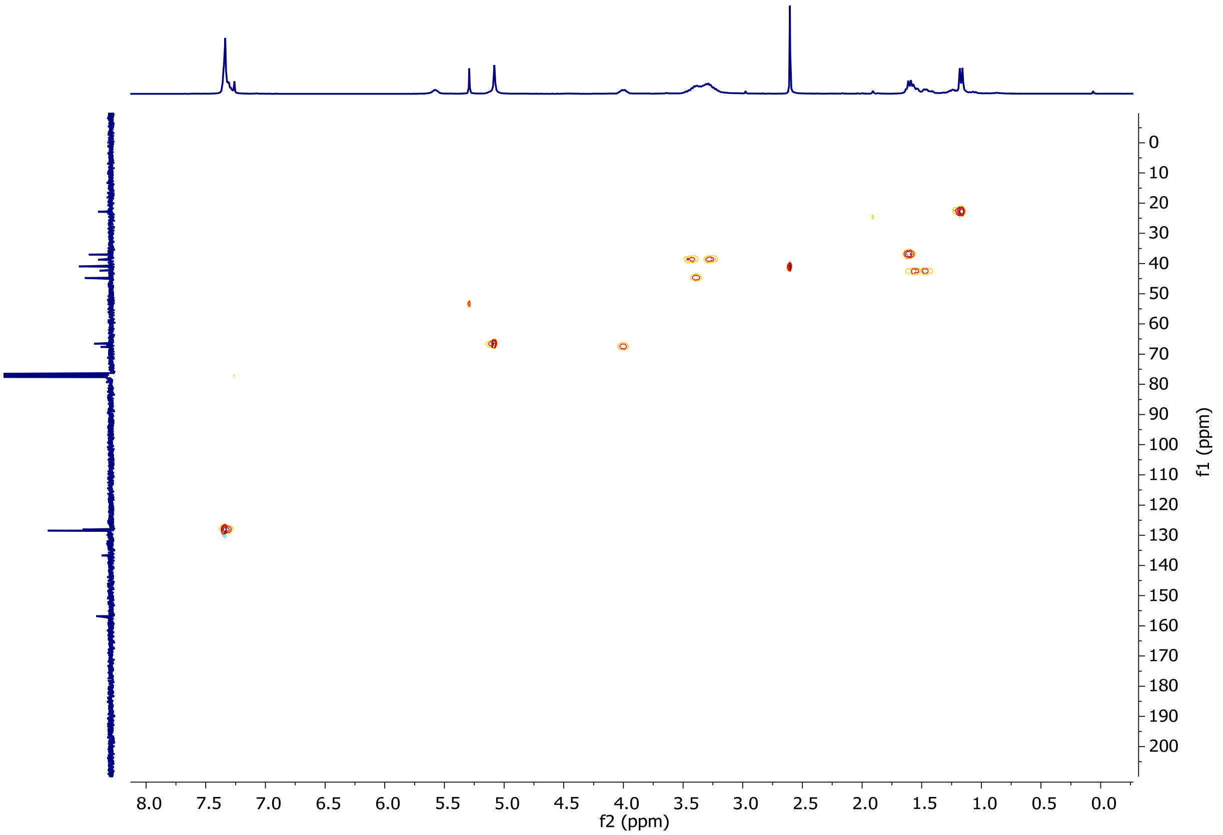

Figure S49. HSQC spectrum in d4-MeOH of product *anti*-**6c**.

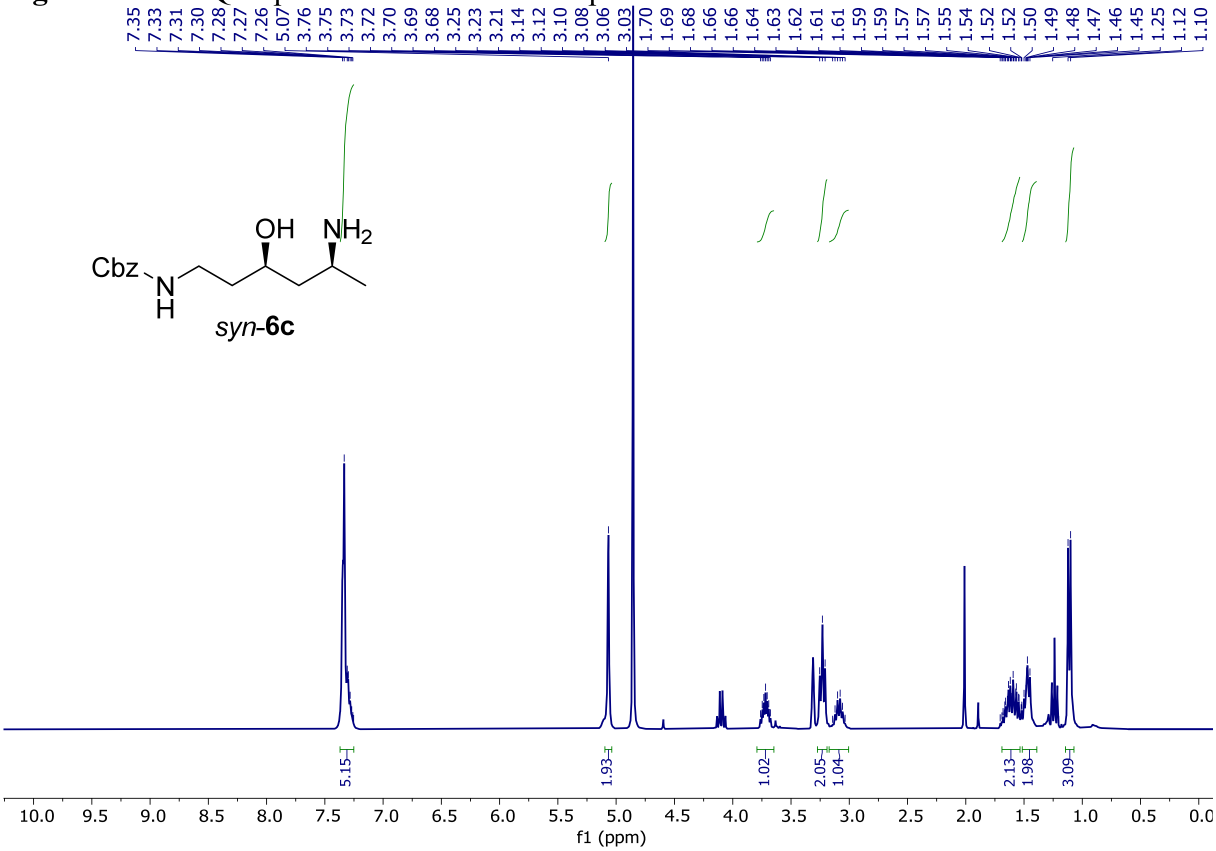

Figure S50. ^{1}H-NMR spectrum in d4-MeOH of product *syn*-**6c**.

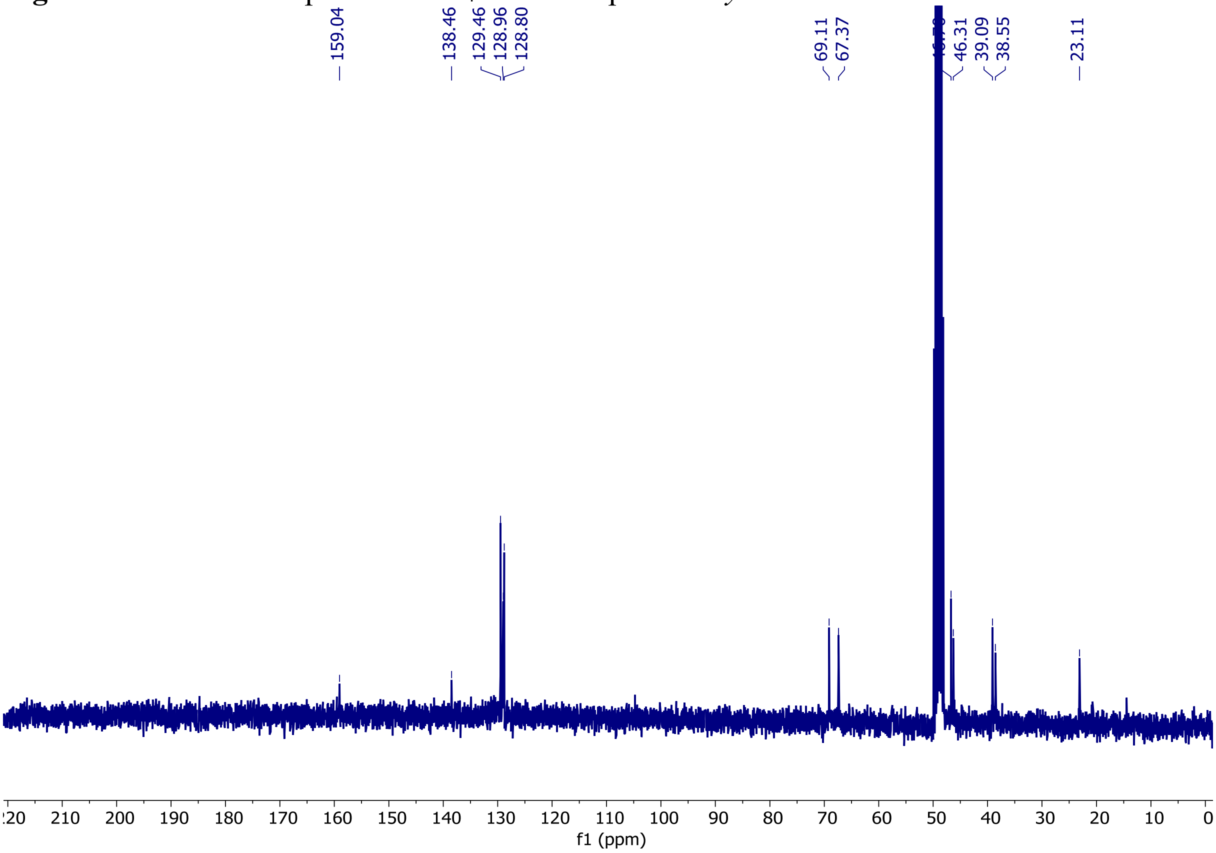

Figure S51. ^{13}C-NMR spectrum in d4-MeOH of product *syn*-**6c**.

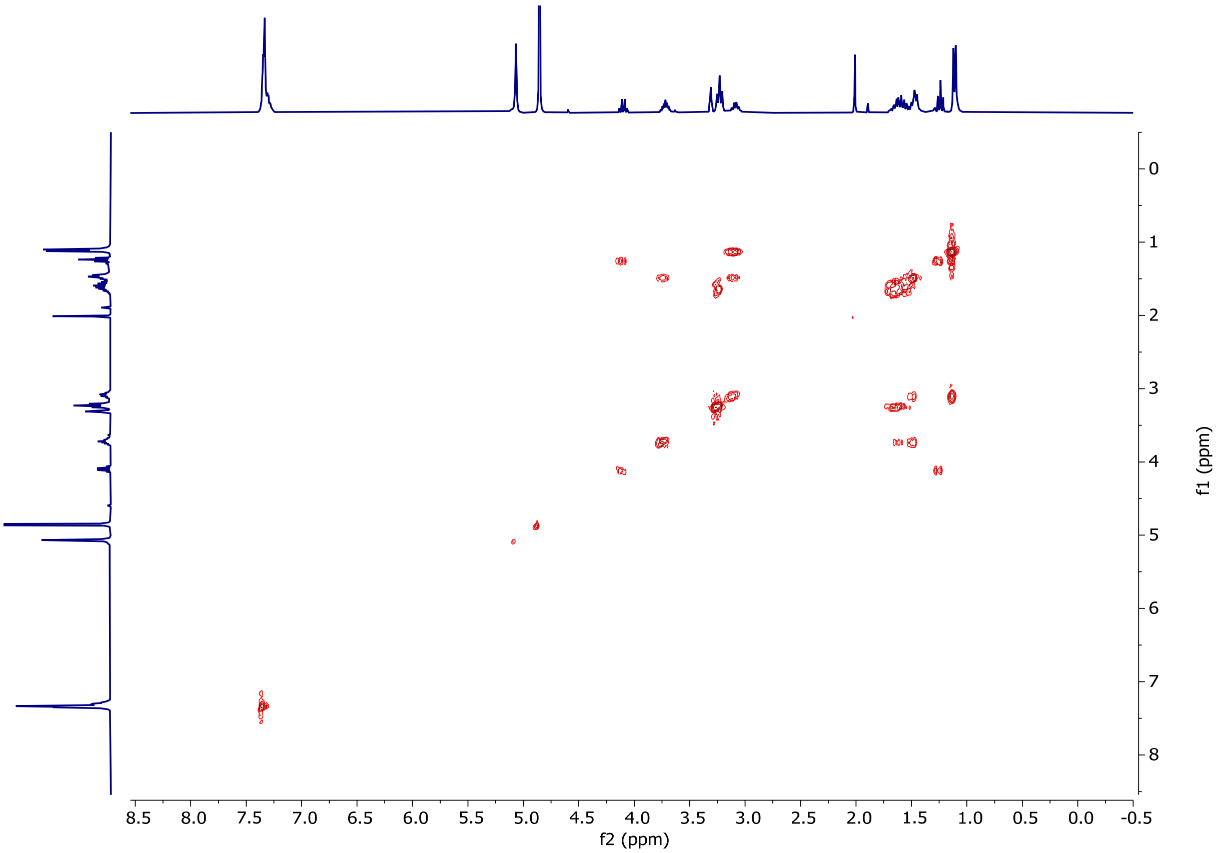

Figure S52. COSY spectrumin d$_4$-MeOH of product *syn*-**6c**.

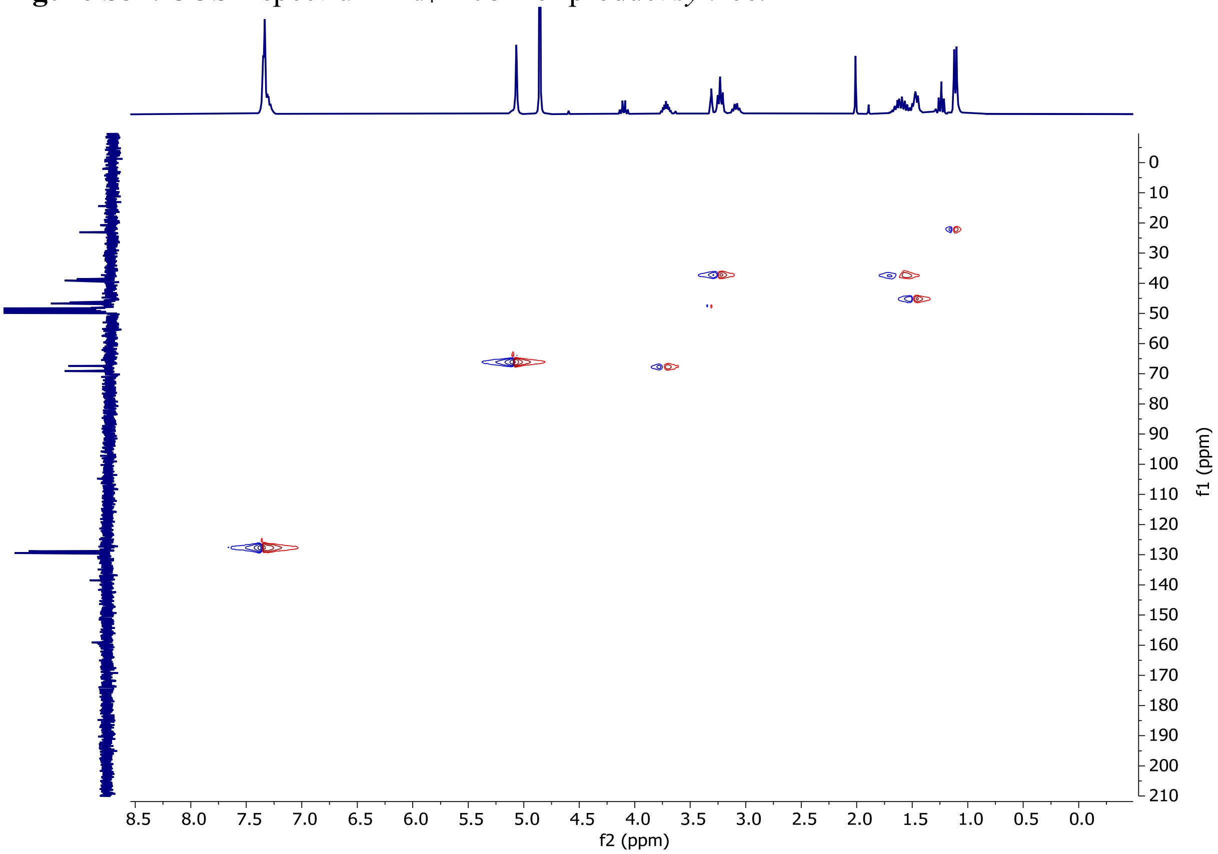

Figure S53. HSQC spectrum in d$_4$-MeOH of product *syn*-**6c**.

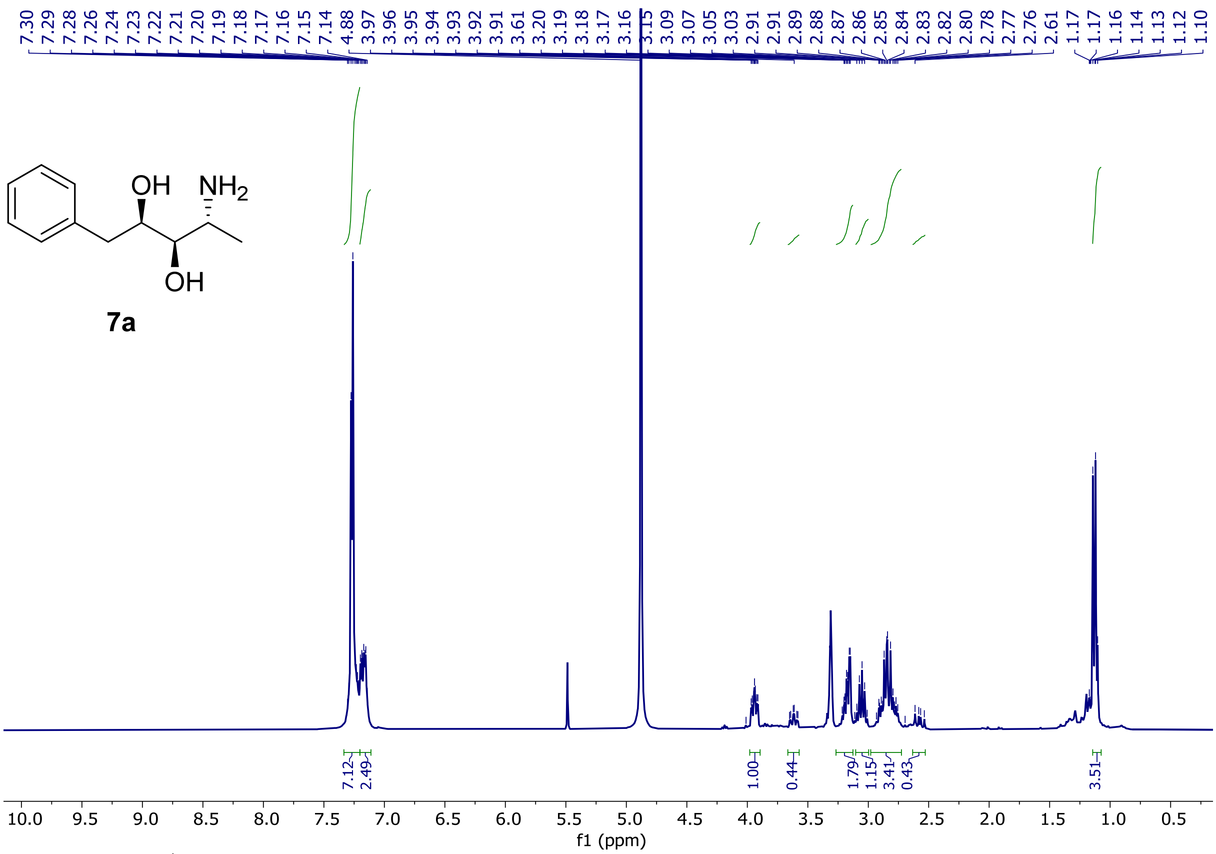

Figure S54. ^{1}H-NMR spectrum in d$_4$-MeOH of product **7a**.

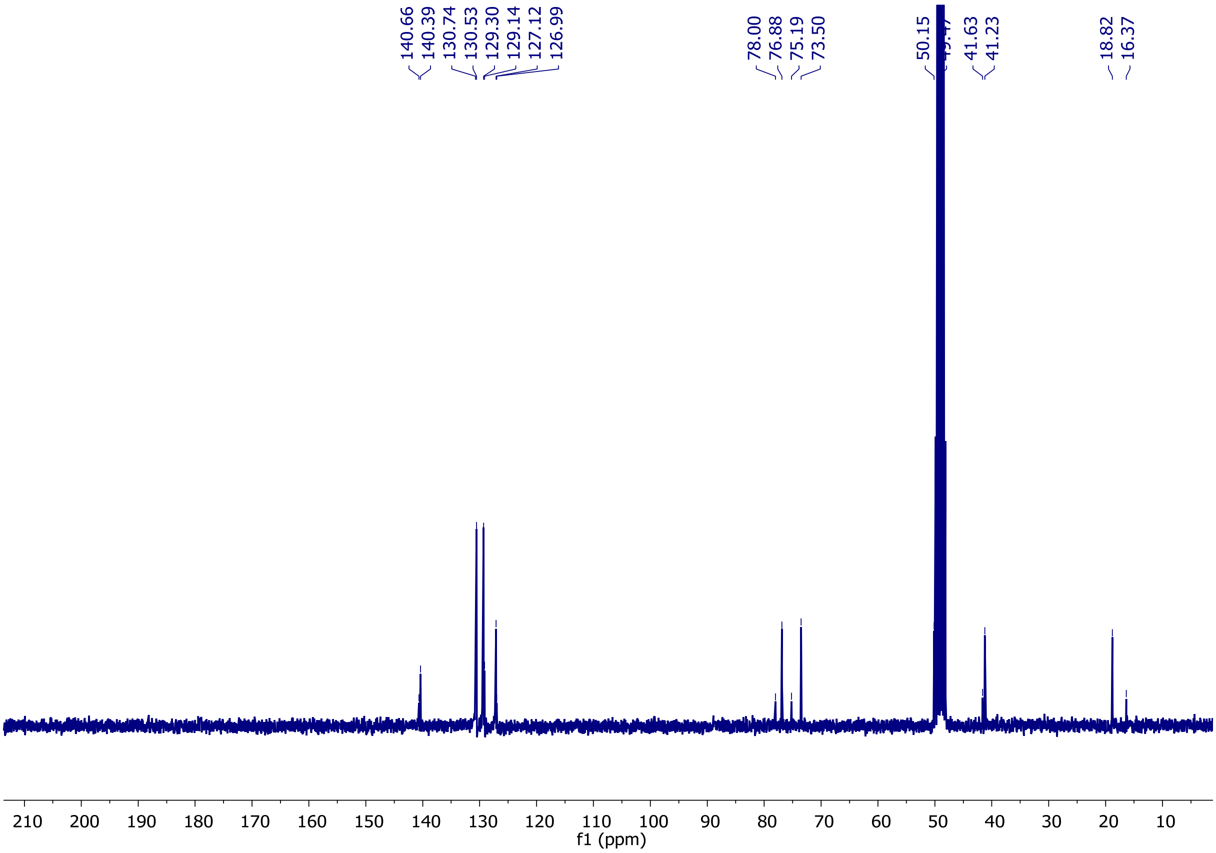

Figure S55. ^{13}C-NMR spectrum in d$_4$-MeOH of product **7a**.

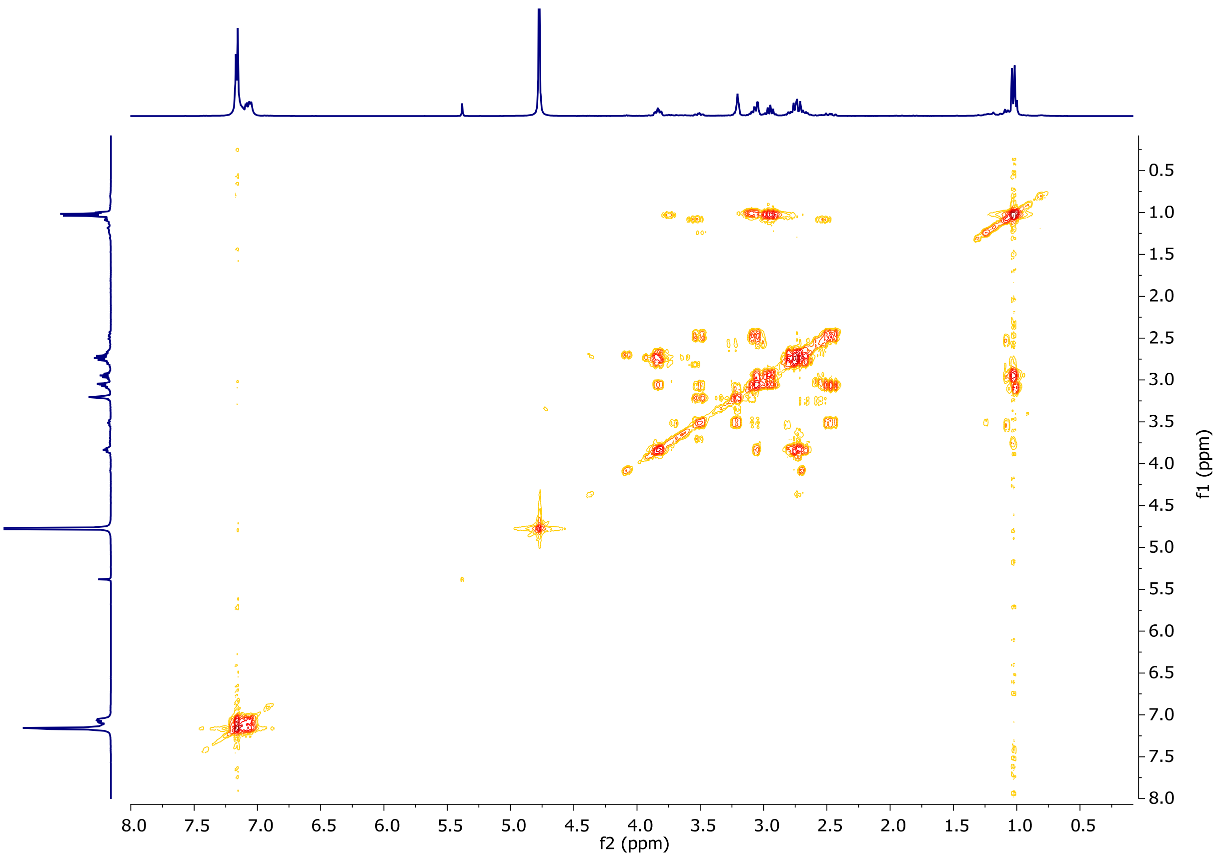

Figure S56. COSY spectrum in d4-MeOH of product **7a**.

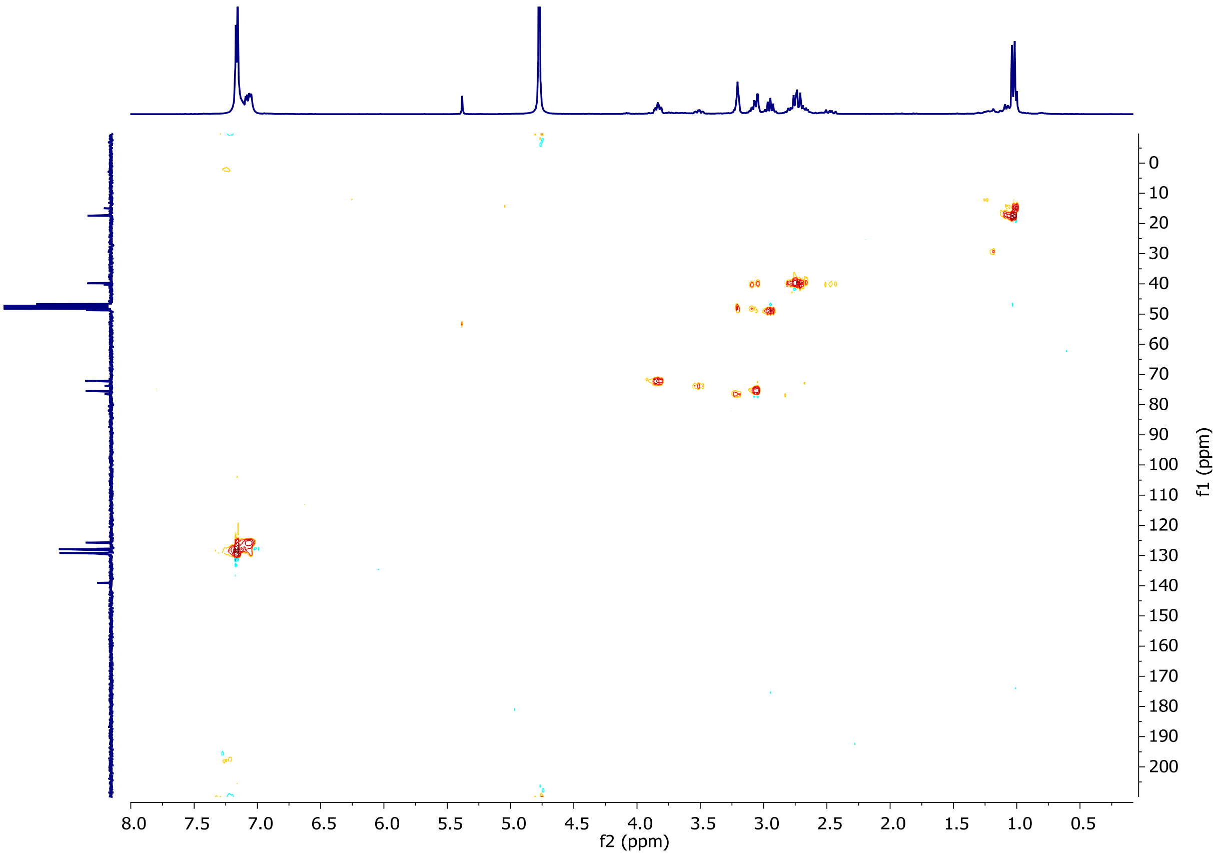

Figure S57. HSQC spectrum in d4-MeOH of product **7a**.

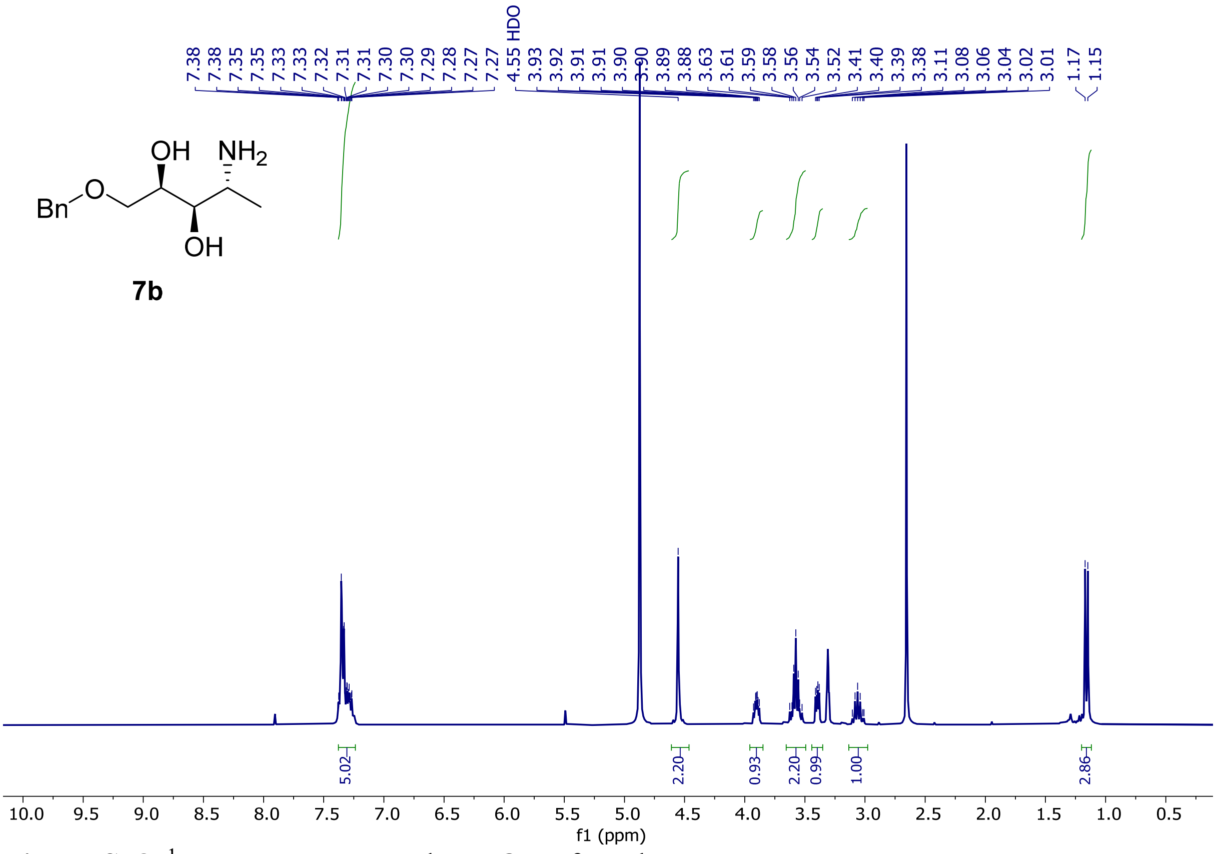

Figure S58. ^{1}H-NMR spectrum d4-MeOH of product **7b**.

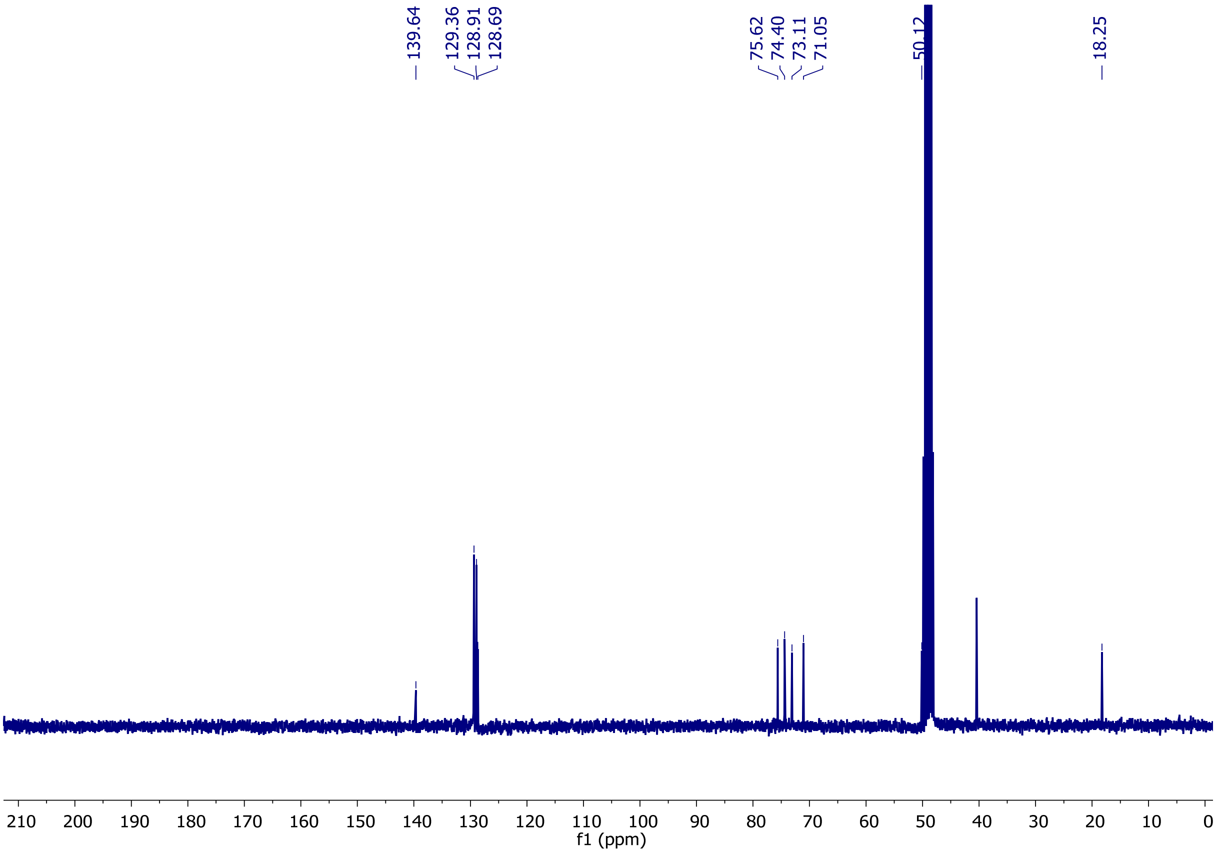

Figure S59. ^{13}C-NMR spectrum d4-MeOH of product **7b**.

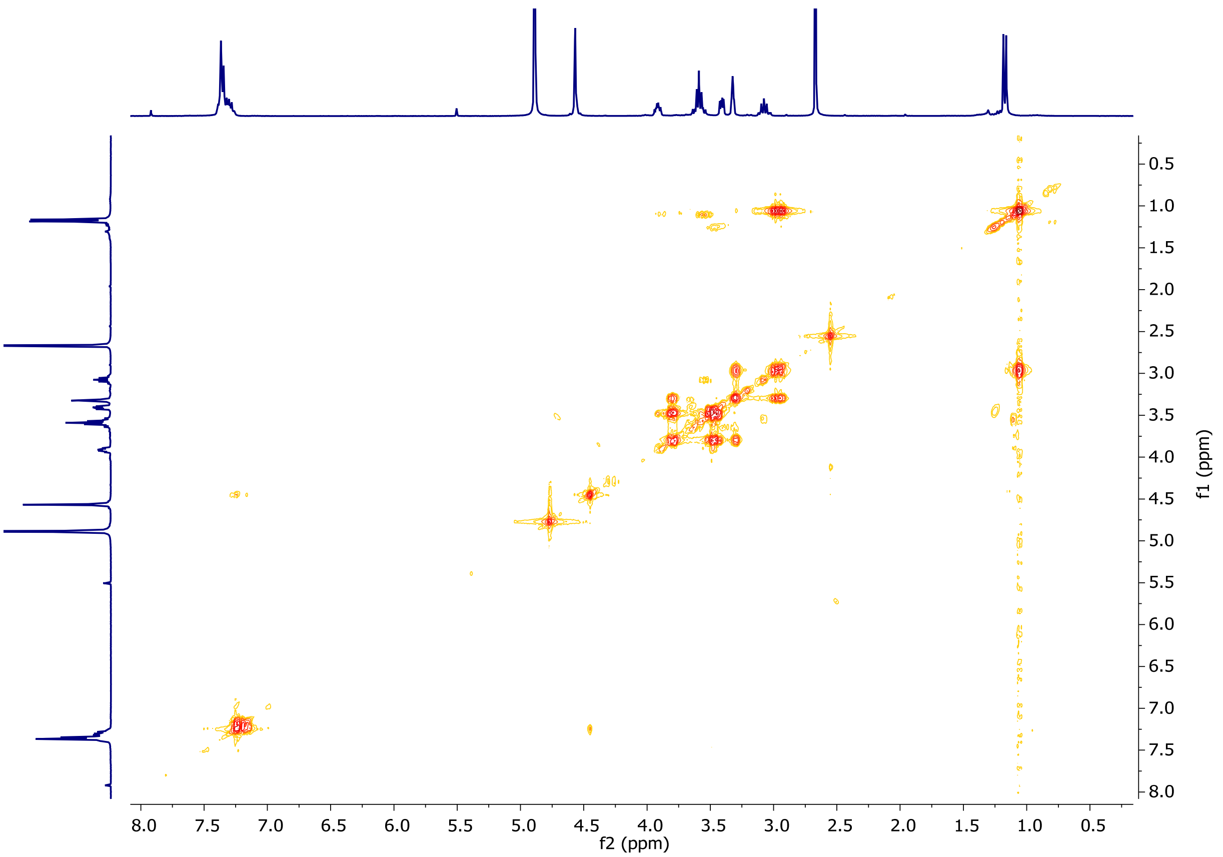

Figure S60. COSY spectrum in d4-MeOH of product **7b**.

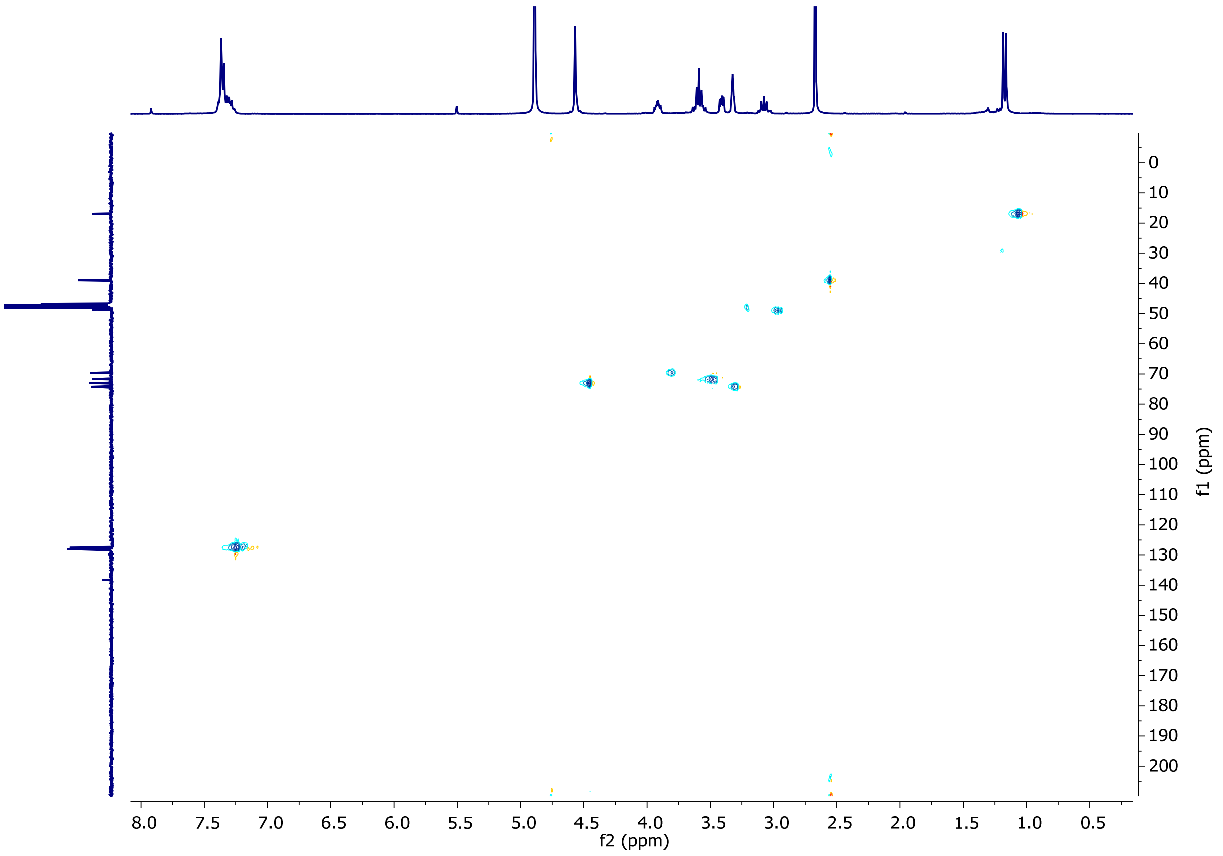

Figure S61. HSQC spectrum in d4-MeOH of product **7b**.

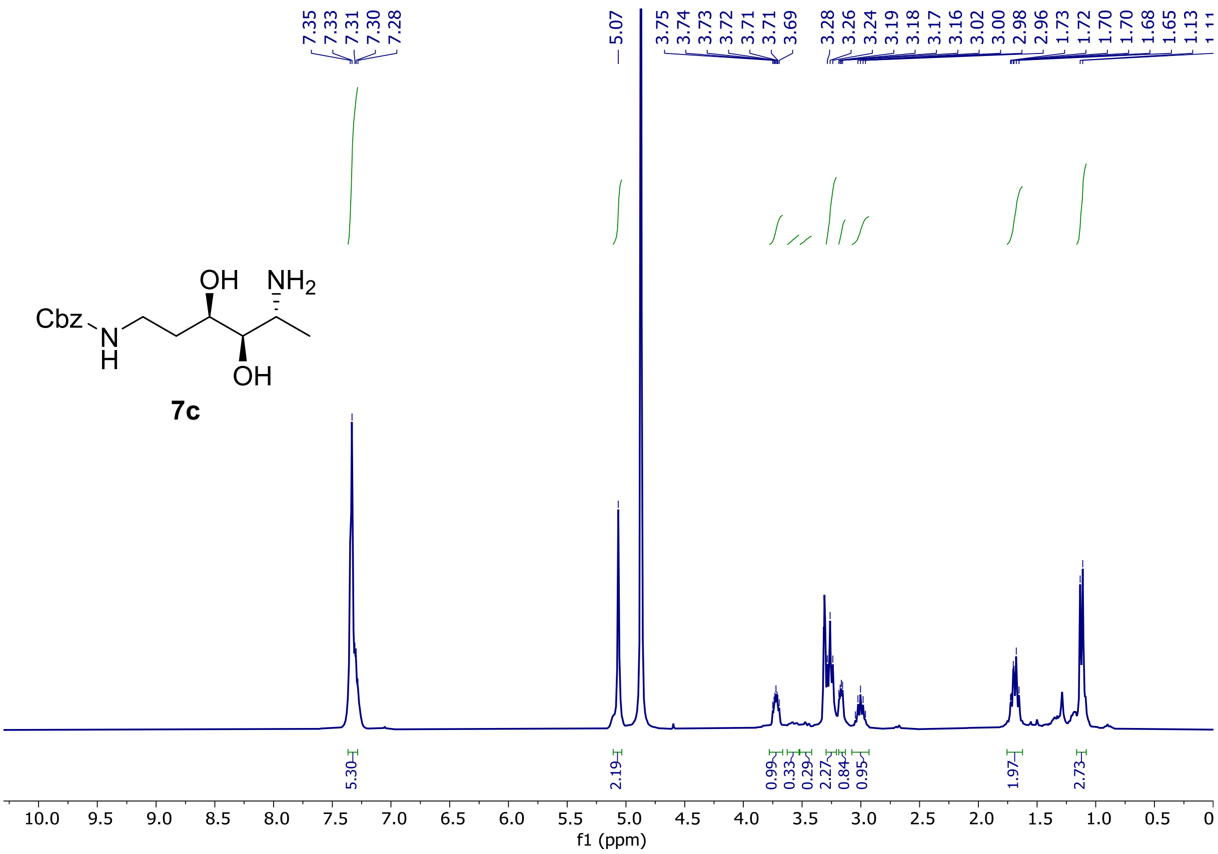

Figure S62. ^{1}H-NMR spectrum in d_4-MeOH of product **7c**.

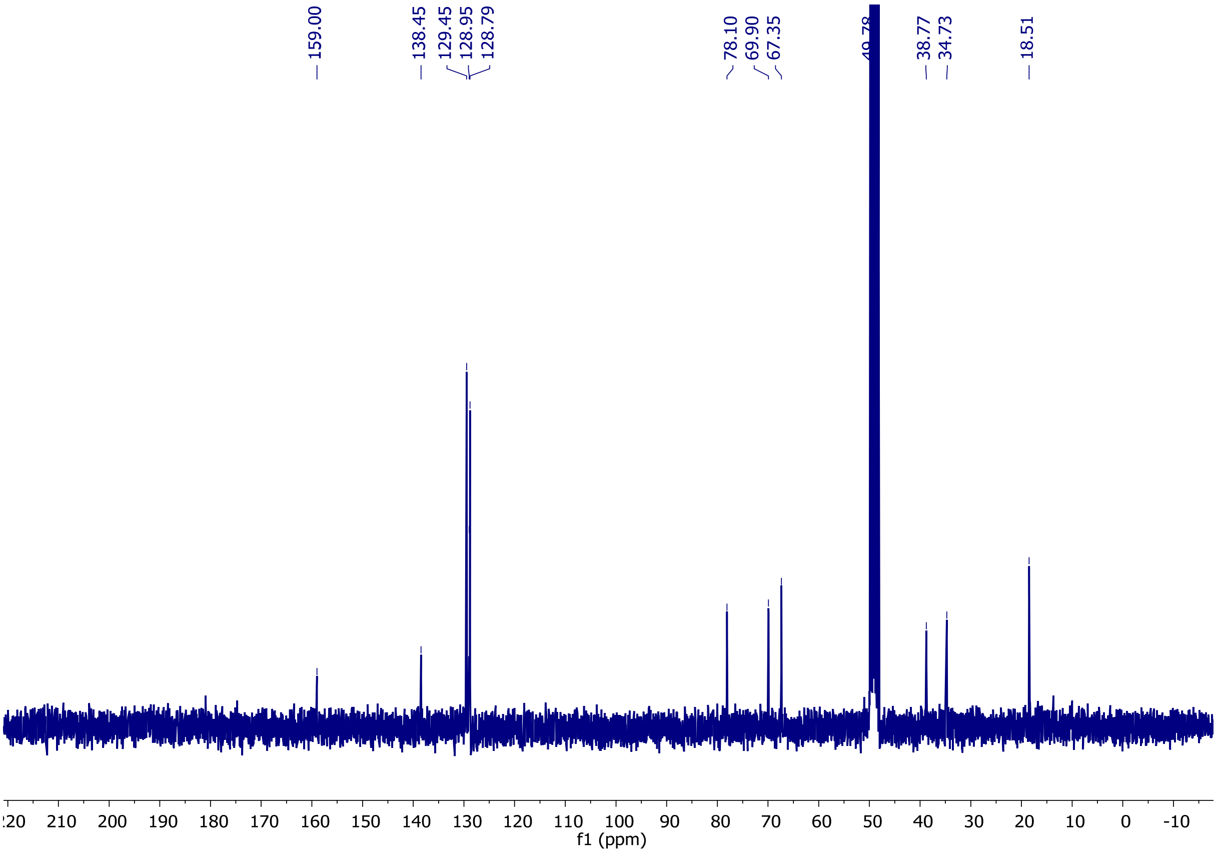

Figure S63. ^{13}C-NMR spectrum d_4-MeOH of product **7c**.

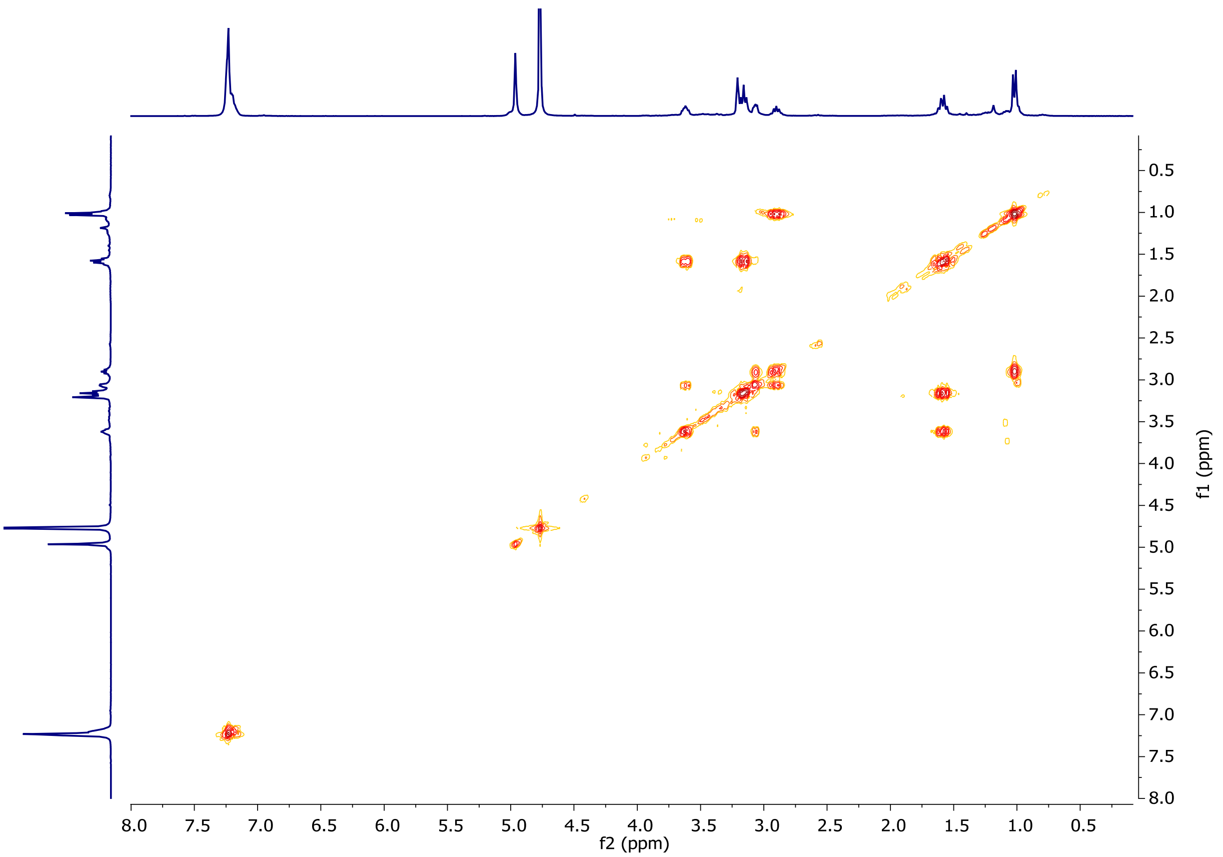

Figure S64. COSY spectrum d4-MeOH of product **7c**.

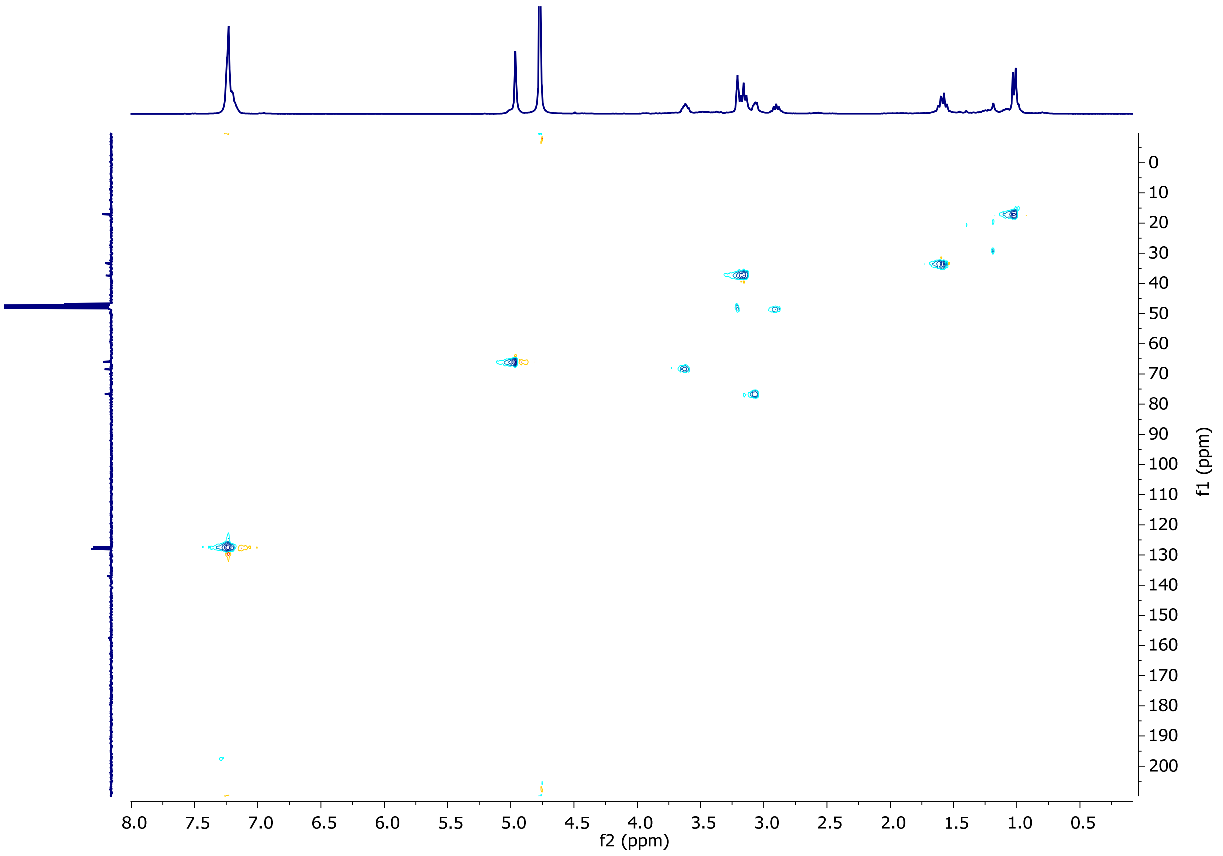

Figure S65. HSQC spectrum in d4-MeOH of product **7c**.

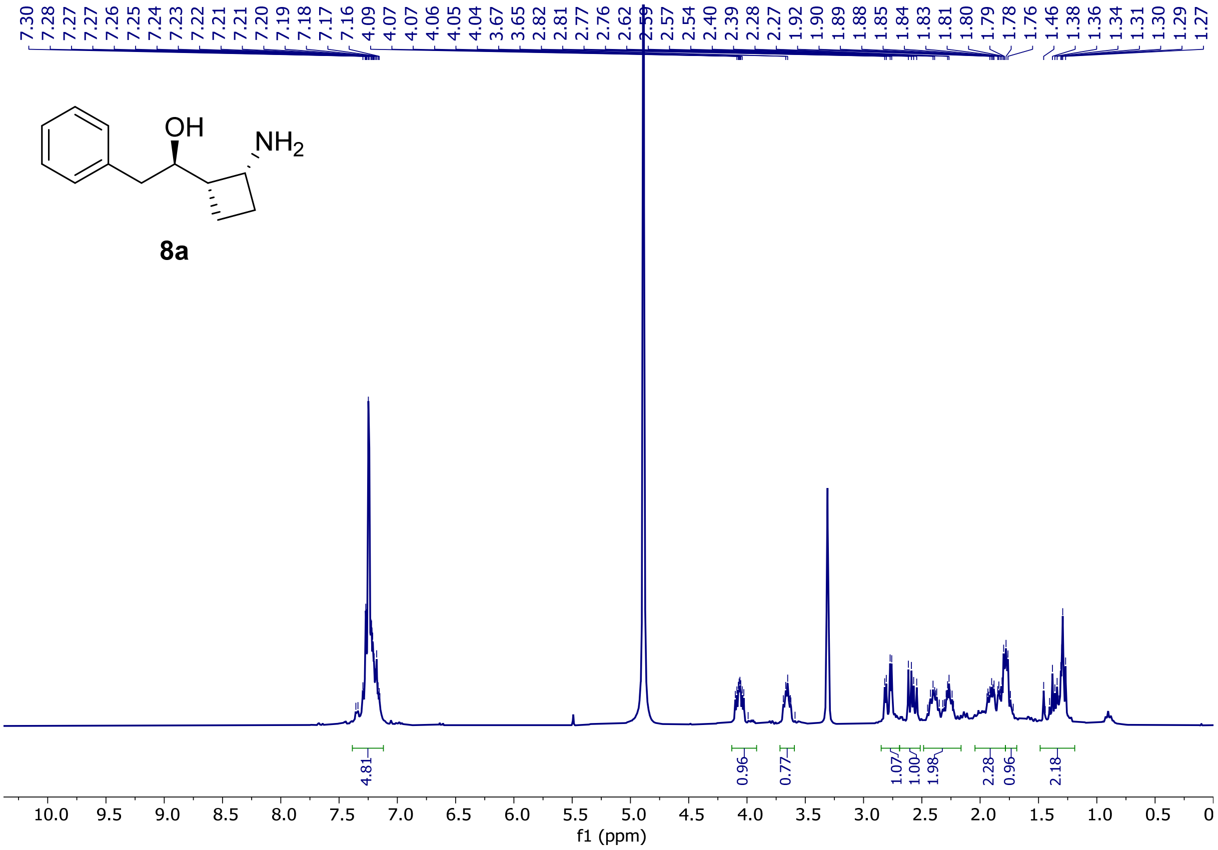

Figure S66. ^{1}H-NMR spectrum in d4-MeOH of product **8a**.

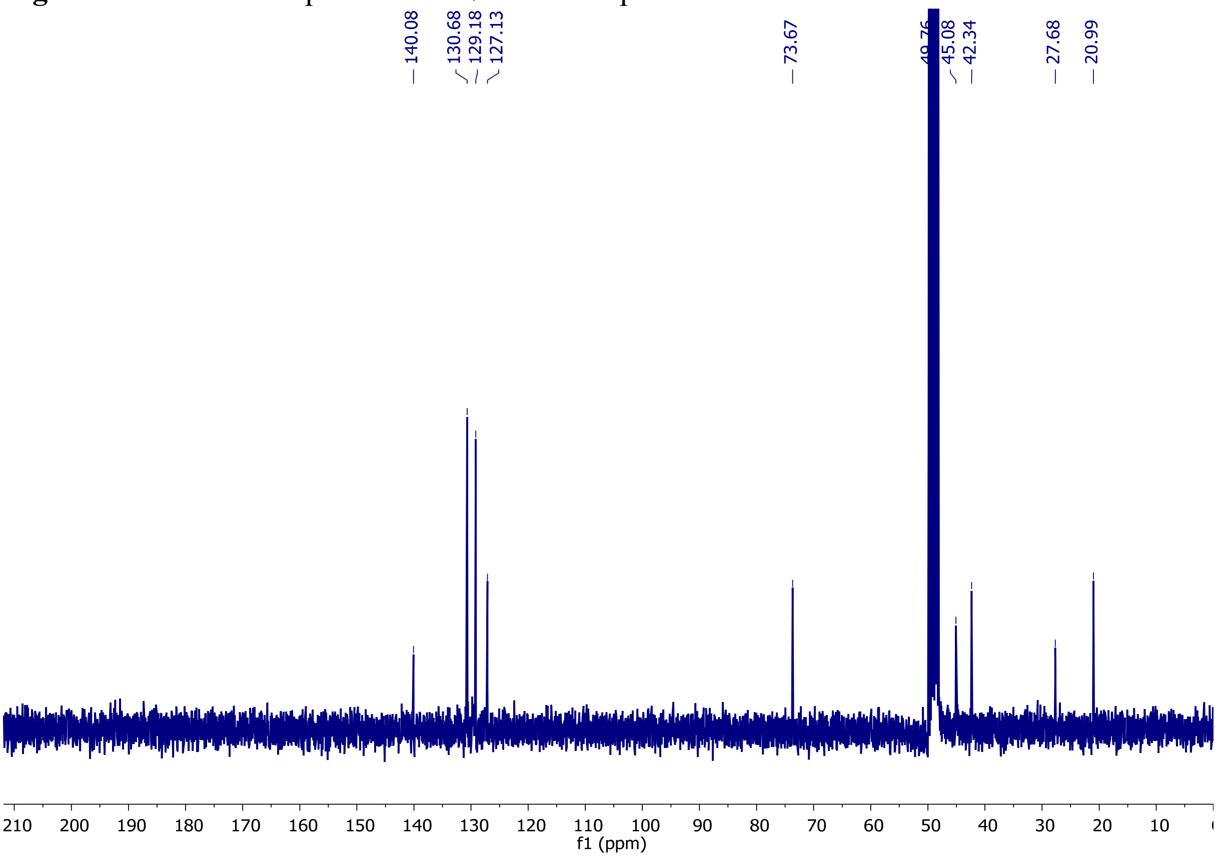

Figure S67. ^{13}C-NMR spectrum d4-MeOH of product **8a**.

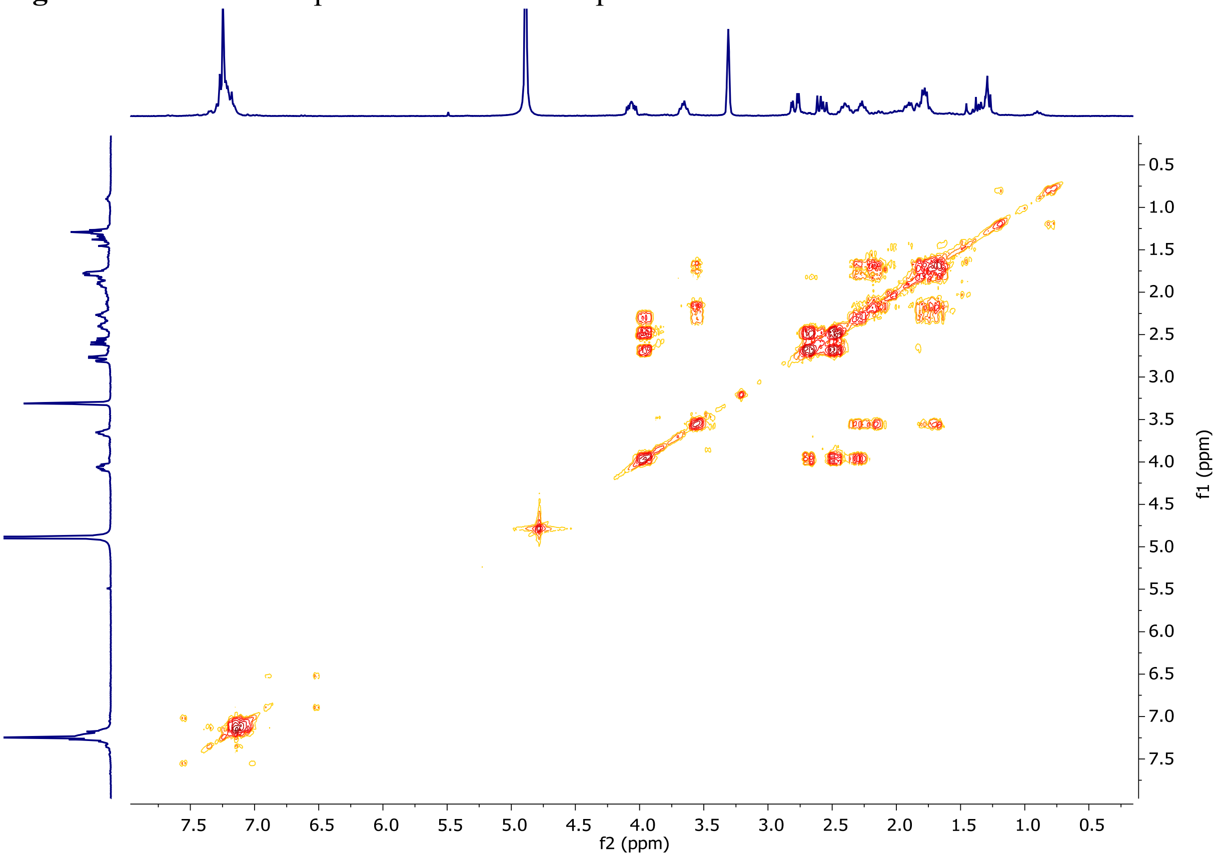

Figure S68. COSY spectrum in d4-MeOH of product **8a**.

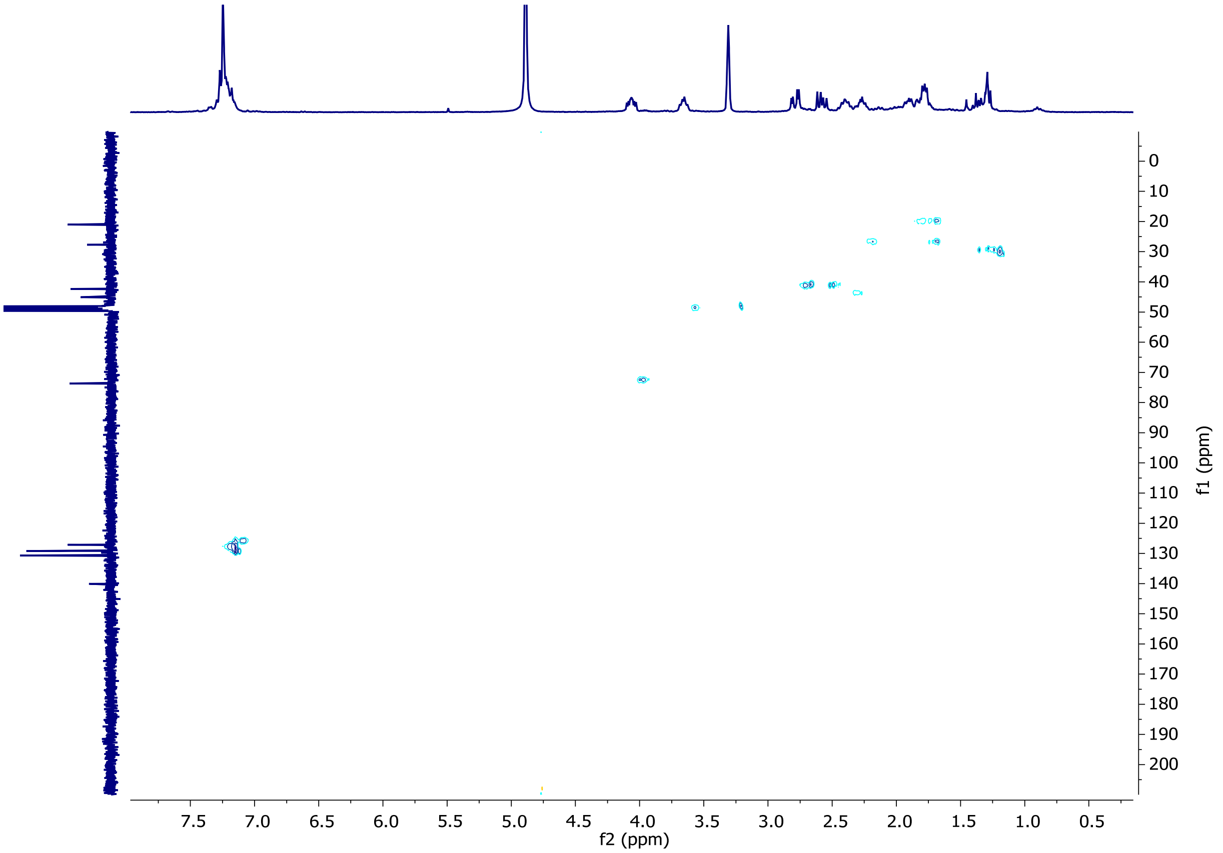

Figure S69. HSQC spectrum in d4-MeOH of product **8a**.

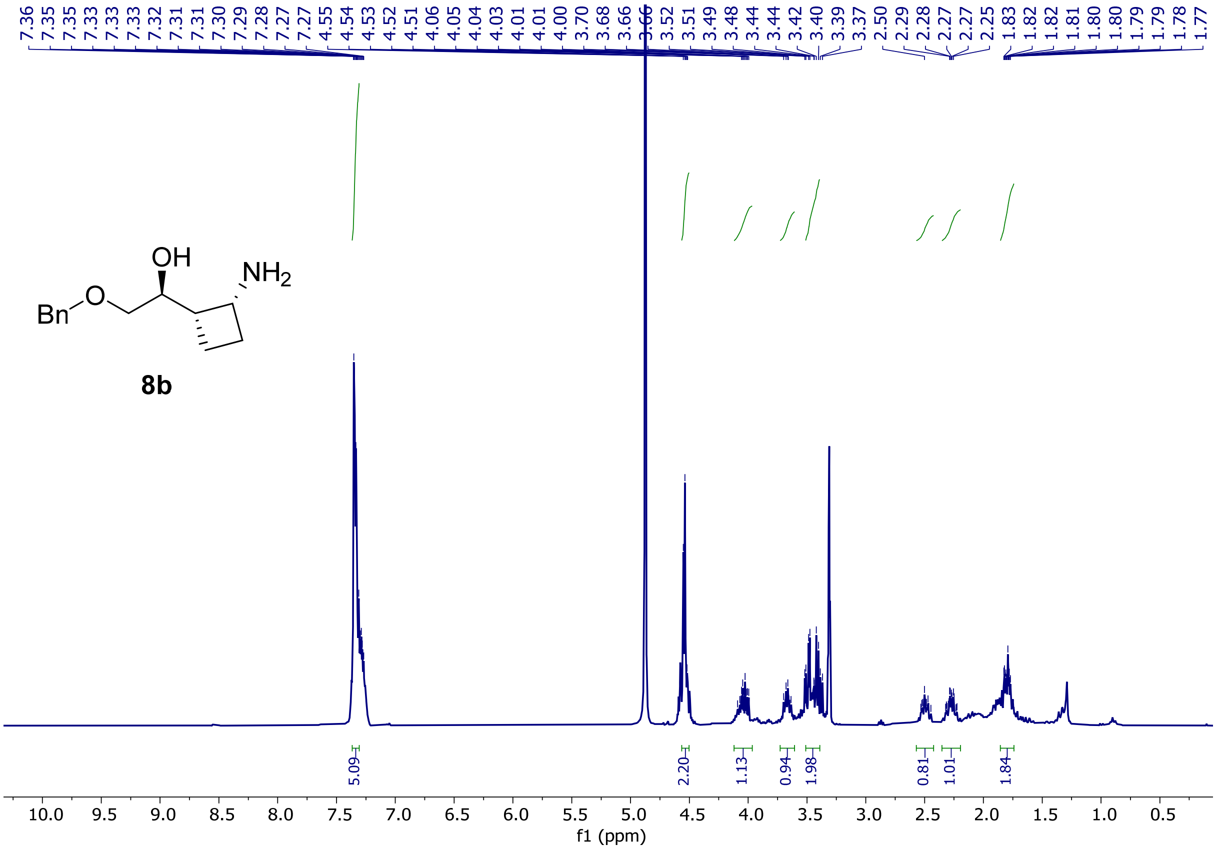

Figure S70. ^{1}H-NMR spectrum in d_4-MeOH of product **8b**.

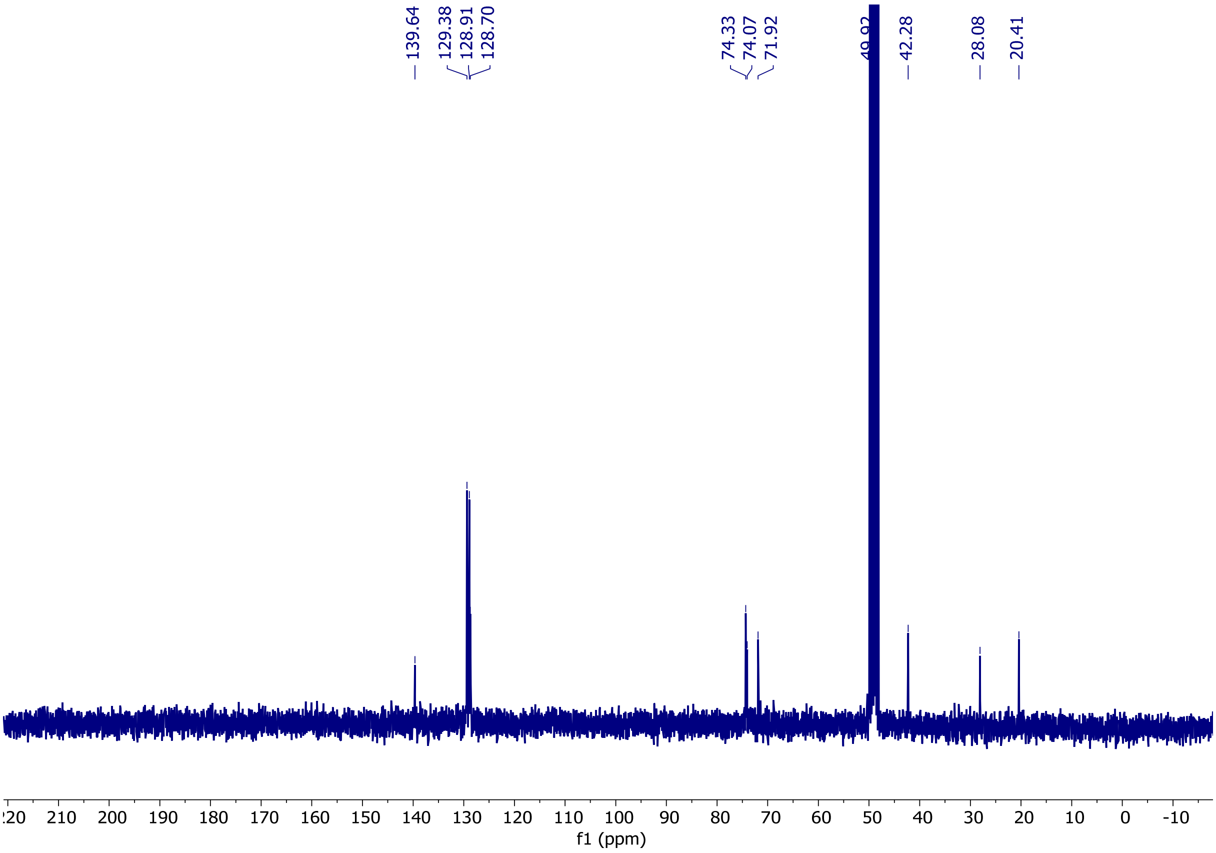

Figure S71. ^{13}C-NMR spectrum in d_4-MeOH of product **8b**.

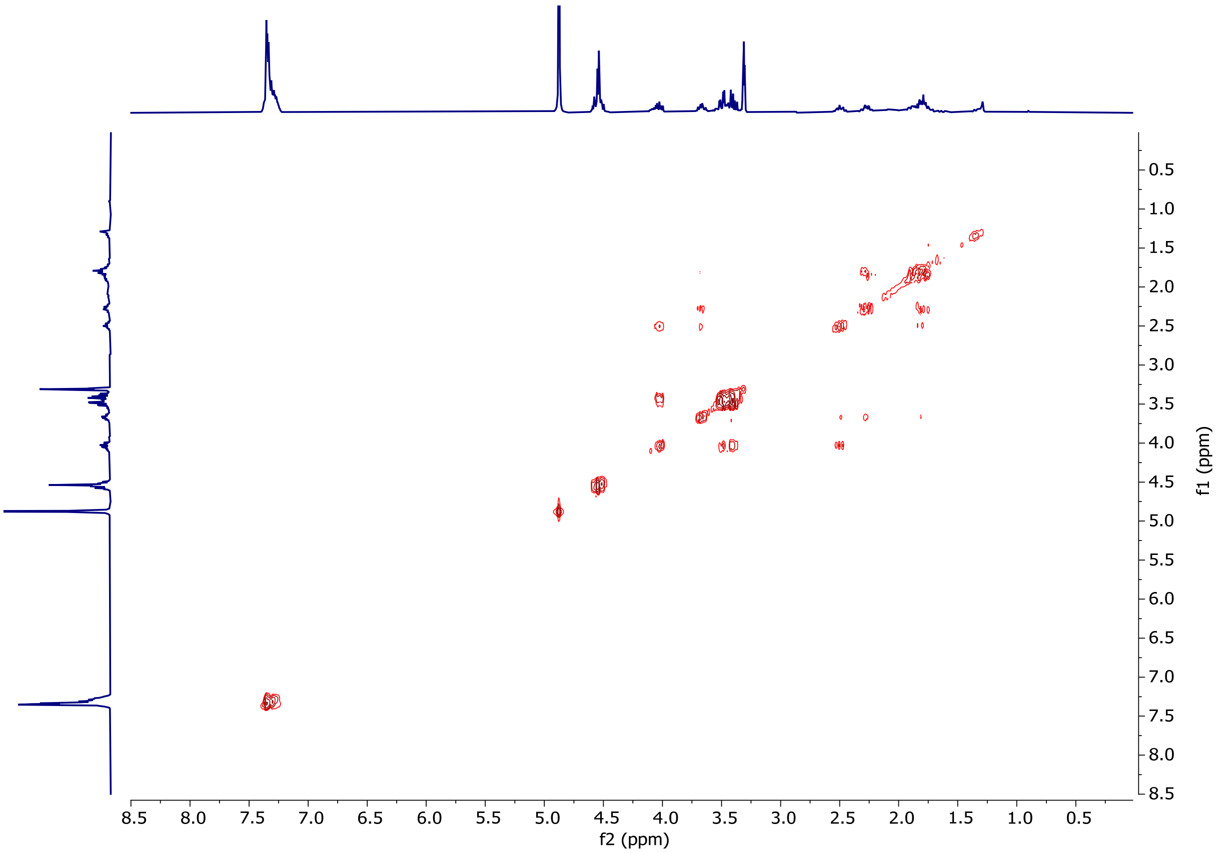

Figure S72. COSY spectrum in d4-MeOH of product **8b**.

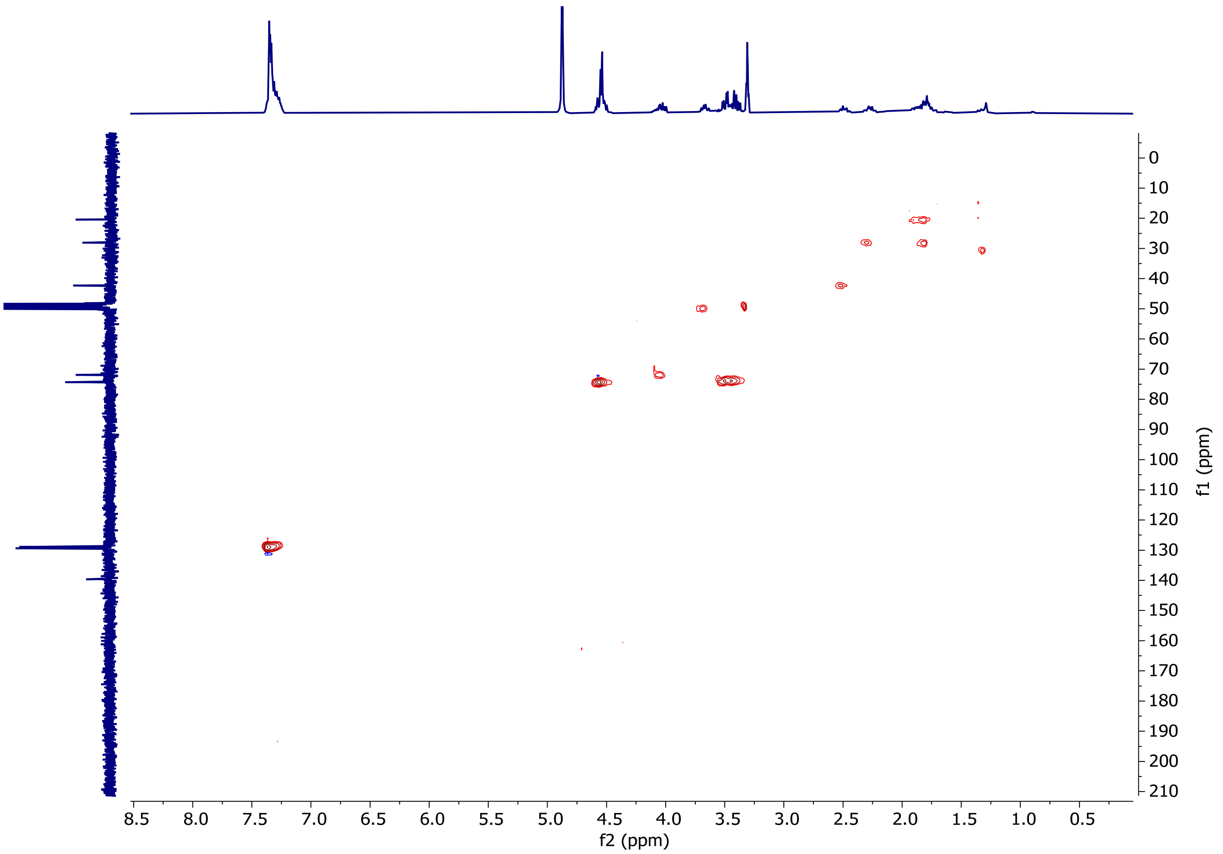

Figure S73. HSQC spectrum in d4-MeOH of product **8b**.

4. References

[1] R. Roldán, K. Hernandez, J. Joglar, J. Bujons, T. Parella, I. Sánchez-Moreno, V. Hélaine, M. Lemaire, C. Guérard-Hélaine, W. D. Fessner, P. Clapés, *ACS Catal* **2018**, *8*, 8804–8809.

[2] H. Kohls, M. Anderson, J. Dickerhoff, K. Weisz, A. Cordova, P. Berglund, H. Brundiek, U. T. Bornscheuer, M. Höhne, *Adv. Synth. Catal.* **2015**, *357*, 1808–1814.

[3] N. H. Meyer, K. Zangger, *Angew. Chem. Int. Ed.* **2013**, *52*, 7143–7146.
